# A DNA microarray survey of gene expression in normal human tissues

**DOI:** 10.1186/gb-2005-6-3-r22

**Published:** 2005-02-14

**Authors:** Radha Shyamsundar, Young H Kim, John P Higgins, Kelli Montgomery, Michelle Jorden, Anand Sethuraman, Matt van de Rijn, David Botstein, Patrick O Brown, Jonathan R Pollack

**Affiliations:** 1 Department of Pathology, Stanford University School of Medicine, 269 Campus Drive, CCSR 3245A, Stanford, CA 94305-5176, USA; 2 Department of Biochemistry, Stanford University School of Medicine, 279 Campus Drive, Stanford, CA 94305-5307, USA; 3 Department of Genetics, Stanford University, Stanford, CA 94305, USA; 4 Howard Hughes Medical Institute, Stanford University School of Medicine, 279 Campus Drive, Stanford, CA 94305-5307, USA; 5 Lewis-Sigler Institute for Integrative Genomics, Princeton University, Princeton, NJ 80544, USA

## Abstract

**Background:**

Numerous studies have used DNA microarrays to survey gene expression in cancer and other disease states. Comparatively little is known about the genes expressed across the gamut of normal human tissues. Systematic studies of global gene-expression patterns, by linking variation in the expression of specific genes to phenotypic variation in the cells or tissues in which they are expressed, provide clues to the molecular organization of diverse cells and to the potential roles of the genes.

**Results:**

Here we describe a systematic survey of gene expression in 115 human tissue samples representing 35 different tissue types, using cDNA microarrays representing approximately 26,000 different human genes. Unsupervised hierarchical cluster analysis of the gene-expression patterns in these tissues identified clusters of genes with related biological functions and grouped the tissue specimens in a pattern that reflected their anatomic locations, cellular compositions or physiologic functions. In unsupervised and supervised analyses, tissue-specific patterns of gene expression were readily discernable. By comparative hybridization to normal genomic DNA, we were also able to estimate transcript abundances for expressed genes.

**Conclusions:**

Our dataset provides a baseline for comparison to diseased tissues, and will aid in the identification of tissue-specific functions. In addition, our analysis identifies potential molecular markers for detection of injury to specific organs and tissues, and provides a foundation for selection of potential targets for selective anticancer therapy.

## Abstract 2

A systematic survey of gene expression in 115 human tissue samples using cDNA microarrays provides a dataset that can be used as a baseline for comparison with expression in diseased tissue.

## Background

DNA microarrays [1,2] have been used to profile gene expression in cancer and other diseases. In cancer, for example, microarray profiling has been applied to classify tumors according to their sites of origin [3-5], to discover previously unrecognized subtypes of cancer [6-11], to predict clinical outcome [12-14] and to suggest targets for therapy [15,16]. However, the identification of improved markers for diagnosis and molecular targets for therapy will depend on knowledge not only of the genes expressed in the diseased tissues of interest, but also on detailed information about the expression of the corresponding genes across the gamut of normal human tissues.

At present there is relatively little data on gene expression across the diversity of normal human tissues [17-20]. Here we report a DNA microarray-based survey of gene expression in a diverse collection of normal human tissues and also present an empirical method for estimating transcript abundance from DNA microarray data.

## Results

### Hierarchical clustering of gene expression in normal tissues

**Figure 1:**
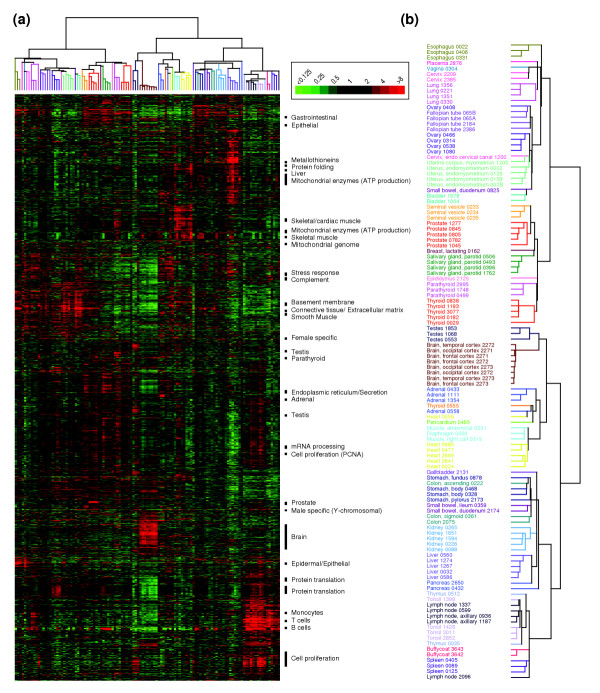
Hierarchical cluster analysis of normal tissue specimens. **(a) **Thumbnail overview of the two-way hierarchical cluster of 115 normal tissue specimens (columns) and 5,592 variably-expressed genes (rows). Mean-centered gene expression ratios are depicted by a log_2 _pseudocolor scale (ratio fold-change indicated); gray denotes poorly-measured data. Selected gene-expression clusters are annotated. The dataset represented here is available as Additional data file 2. (**b) **Enlarged view of the sample dendrogram. Terminal branches for samples are color-coded by tissue type.

To survey gene expression across normal human tissues, we analyzed 115 normal tissue specimens representing 35 different human tissue types, using cDNA microarray representing 26,260 different genes (see Materials and methods). To explore the relationship among samples and underlying features of gene expression, we applied an unsupervised two-way (that is, genes against samples) hierarchical clustering method using the 5,592 cDNAs (representing 3,960 different UniGene clusters [21]) whose expression varied most across samples (Figure 1a; also see Additional data file 2). Overall, tissue samples clustered in large part according to their anatomic locations, cellular compositions or physiologic functions (Figure 1b). For example, lymphoid tissues (lymph node, tonsil, thymus, buffy coat and spleen) clustered together, as did gastrointestinal tissues (stomach, gall bladder, liver, pancreas, small bowel and colon), muscular tissues (heart and skeletal muscle), secretory tissues (parathyroid, thyroid, prostate, seminal vesicle and salivary gland), and female genitourinary tissues (ovary, fallopian tube, uterus, cervix and bladder). Brain and testis were also found to cluster together, largely because genes encoding ribosomal proteins and lymphoid-specific genes were expressed at particularly low levels in both tissues, the latter possibly reflecting immunological privilege [22].

The two-way unsupervised analysis also identified clusters of coexpressed genes (annotated in Figure 1), which represented both tissue-specific structures and systems (discussed further below) and coordinately regulated cellular processes. For example, on the basis of the shared characteristics of well annotated genes in the clusters, we identified clusters representing cell proliferation [23], mitochondrial ATP production, mRNA processing, protein translation and endoplasmic reticulum-associated protein modification and secretion. Interestingly, proliferation, mitochondrial ATP production and protein translation were each represented by two distinct clusters of genes, suggesting that subsets of these functions might be differentially regulated among different tissues. One gene cluster corresponded to sequences on the mitochondrial chromosome [24]; we interpret this feature to reflect the relative abundance of mitochondria in each tissue sample.

### Identifying tissue-specific gene expression

**Figure 2:**
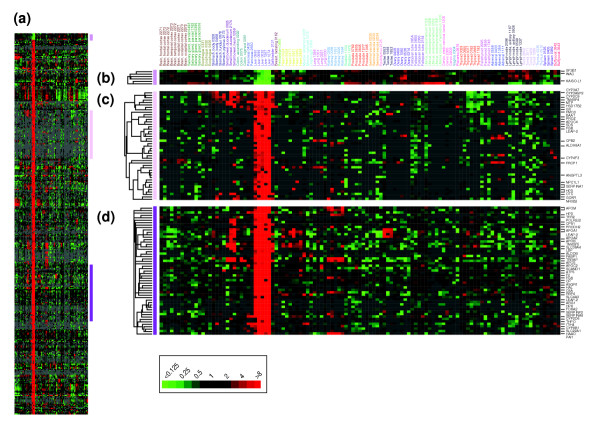
Liver-specific gene expression. **(a) **Thumbnail overview of a hierarchical cluster of 115 normal tissue specimens and 353 variably expressed genes identified using the SAM method (see Materials and methods) as selectively expressed in liver (false discovery rate = 0.12%). Genes are hierarchically clustered, while samples are grouped by tissue type and ordered according to anatomical location/function. Mean-centered gene-expression ratios are depicted by a log_2 _pseudocolor scale (indicated); samples are color-coded by tissue type. **(b-d) **Selected gene-expression clusters (locations indicated by vertical colored bars). Because of space limitations, only named genes (and not expressed sequence tags (ESTs)) are indicated. Tissue-specific genes identified for other tissues are available as Additional data files 3 and 6.

While tissue-specific gene expression features were apparent in the hierarchical cluster, in order to identify tissue-specific genes more systematically we performed supervised analyses using the significance analysis of microarrays (SAM) method ([25], see Materials and methods). Tissue-specific genes were identified for all tissues analyzed, and included named genes with known tissue-specific functions, as well as named genes and anonymous expressed sequence tags (ESTs) that had not been previously characterized as having tissue-specific functions. For example, while the set of liver-specific genes (Figure 2) included, as expected, genes encoding blood-clotting factors (for example, *F2*, *F7*), complement components (*C1R*, *C2*), lipid (*APOB*, *APOE*) and metal transport proteins (*TF*, *CP*), and proteins for detoxification (*CYP2D6*, *CYP3A7*), amino acid metabolism (*PAH*, *HAL*) and carbohydrate metabolism (*G6PT1*, *GYS2*), other intriguing genes, for example, *WRNIP1 *(Werner helicase interacting protein 1), *BIRC5 *(survivin), *ANGPTL3 *(angiopoietin-like 3), and *CNTNAP1 *(contactin associated protein 1), were also identified as selectively expressed in liver. The new connections these results might make between our knowledge of the gene and its product on the one hand, and our knowledge of the physiological functions, cellular characteristics and pathologies of a specific organ, on the other, are a step towards better understanding of both the genes and the organs. Interestingly, we also identified a smaller number of genes displaying selectively decreased expression in some organs, for example, splicing factor *SF3B1 *in the liver (Figure 2b): we speculate that the decreased expression of such genes may have a role in regulating cellular/tissue differentiation. Tissue-specific genes characteristically expressed in each of the tissues we examined are viewable in Additional data file 6 (see also Additional data file 3).

**Figure 3:**
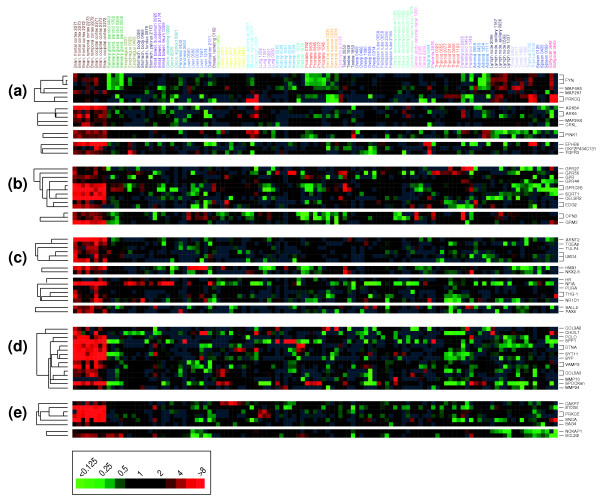
Brain-selective expression of functionally annotated gene sets. Hierarchical cluster of 115 normal tissue specimens and annotated gene sets representing the following examples of **(a-c) **specific molecular functions (a) tyrosine kinase, (b) G-protein-coupled receptor, (c) transcription factor, **(d) **cellular components (extracellular matrix) or **(e) **biological processes (programmed cell death). Samples are ordered as in Figure 2. Genes are ordered by hierarchical clustering. For gene selection, we considered genes that were well measured in at least 50% of samples; no ratio-fold cutoff was applied. Only features representing brain-specific expression are shown here; the complete clusters are available as Additional data files 4 and 7.

Recent efforts by the Gene Ontology (GO) Consortium have resulted in the systematic annotation of genes, ascribing genes to specific biological processes, cellular components and molecular functions [26]. This annotation system, while rudimentary, facilitates the systematic exploration of the expression of genes reflecting specific biological processes, cellular components and molecular functions in these normal tissues. For example, the gene sets encoding tyrosine kinase, G-protein-coupled receptor and transcription factor functions, as well as components of the extracellular matrix and the process of programmed cell death, each demonstrate tissue-specific patterns of expression (Figure 3; see also Additional data files 4 and 7).

### Estimating transcript abundance

DNA microarray experiments are often performed as comparative two-color hybridizations, permitting precise quantification of the ratio of each gene's expression between two samples. In the experiments reported here, each tissue sample was compared by hybridization to the same 'common reference' mRNA (see Materials and methods), a standard experimental design permitting the comparison of expression across all samples [27]. Therefore, the primary measurements give us a precise picture of the variation in relative levels of each gene's expression among the samples. While this information is sufficient for many purposes, a quantitative comparison of the expression levels of transcripts of different genes is also of interest, for example in selecting especially highly expressed genes for potential diagnostic markers or therapeutic targets. Single-channel fluorescence intensities can provide a crude estimate of the relative transcript abundance of different genes, but do not control for the variable quantities of spotted DNA.

**Figure 4:**
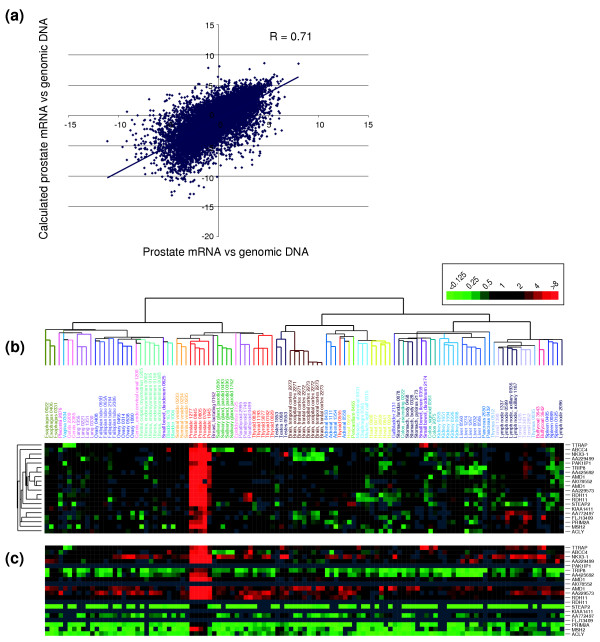
Estimating relative transcript abundance. **(a) **Comparison of transcript levels estimated either directly by hybridization of prostate sample mRNA versus normal female genomic DNA, or indirectly by multiplying the ratio of prostate sample mRNA vs common reference mRNA by the ratio of common reference mRNA vs normal female genomic DNA. The correlation value (R) is indicated. **(b) **Prostate-specific gene-expression cluster, extracted from the hierarchical cluster shown in Figure 1a, is displayed as mean-centered relative gene expression (ratio-fold change scale indicated). **(c) **The same gene-expression feature as in (b), is now displayed as transcript abundance (relative to the average transcript level for all expressed genes), calculated indirectly using the common reference mRNA versus normal female genomic DNA hybridization data.

To estimate transcript levels for our dataset, we used microarray hybridization to compare the common reference mRNA against normal female genomic DNA. We reasoned that, for each gene on the microarray, the ratio of mRNA to genomic DNA should reflect the relative level of transcript in the common reference compared to normal genomic DNA (for which each gene is present in two copies per cell). For each tissue sample in our study, the ratio of expression for each gene in that sample versus common reference mRNA, multiplied by the ratio for that gene in common reference mRNA versus normal genomic DNA, would then approximate transcript abundance. To test our approach, we compared our estimates of transcript levels for a single prostate specimen, calculated either indirectly using the common reference mRNA versus genomic DNA ratios, or calculated through a direct hybridization comparison of prostate sample mRNA versus normal female genomic DNA. Our results show high concordance for the prostate sample (Figure 4a); comparable results were obtained in a similar analysis using liver, breast, heart and kidney specimens (data not shown).

The utility of this approach is illustrated for the cluster of prostate-specific genes (derived from the hierarchical cluster in Figure 1), and is evident on comparing results depicting the relative level of each gene's expression in different samples (Figure 4b), and the relative levels of transcripts for different genes (Figure 4c). While all genes within the prostate-specific cluster were expressed at relatively increased levels in prostate compared with other tissues, estimates of transcript abundance indicated that only a subset of these genes was highly expressed in the prostate (Figure 4c). For example, *RDH11 *was highly expressed in prostate and was expressed at lower levels in other tissues, while *STEAP2 *was expressed at low levels in prostate and displayed very little or no expression in other tissues. For each of the tissue types, transcripts identified as both highly abundant and tissue specific are displayed in Additional data files 5 and 8 (for the transcript levels of all variably expressed genes, see Additional data file 2).

## Discussion

The main objective of our study was to survey variation in gene expression across a diverse set of normal human tissue types. We have reported here a cDNA microarray gene-expression dataset profiling approximately 26,000 human genes across 115 human tissue specimens representing 35 different tissue types. An unsupervised, two-way hierarchical clustering of the genes whose expression varied most across samples showed that at the level of gene expression, the relationship among tissues was in large part based on their anatomic locations, cellular compositions and physiologic functions. Tissue-specific features of gene expression were readily discernable in the hierarchical cluster, as were gene-expression features related to specific cellular processes (as inferred from the named genes within these features). Of particular importance, the function of uncharacterized ESTs might be deduced by virtue of their inclusion in one of these clusters. Supervised analysis also identified genes selectively expressed in each of the tissues types studied, and the analysis of functionally annotated gene sets provided information on the tissue distribution of specific biological processes, cellular components and molecular functions.

We have also reported here the application of mRNA versus genomic DNA hybridizations for estimating transcript abundances for expressed genes. Knowledge of transcript abundance should prove useful in prioritizing candidate genes for use as diagnostic markers or therapeutic targets, for which more highly expressed genes might be more tenable candidates. It is worth pointing out that our approach for estimating absolute transcript levels should be applicable to any cDNA microarray study incorporating a common reference mRNA.

While many investigators have been using DNA microarrays to profile gene expression in cancer and other human diseases, scant data exist on profiles of gene expression across the diversity of normal human tissues. Our cDNA-microarray-based survey of gene expression in normal human tissues provides a publicly accessible dataset which can be used in future analyses aimed at better understanding the physiology of various normal tissues; developing a baseline for comparison to diseased tissues, including cancer; identifying tissue-specific diagnostic markers that signify tissue injury; discovering tissue-specific therapeutic targets (for example, for treatment of prostate cancer); and identifying tumor-specific diagnostic markers and therapeutic targets, for which minimal expression in the collection of normal adult human tissues is desirable.

## Conclusions

We have used cDNA microarrays to survey gene expression across a diverse set of normal human tissues. Using unsupervised and supervised analyses, we have identified tissue-specific patterns of gene expression. Furthermore, by comparative hybridization to normal genomic DNA, we were able to estimate transcript abundances and identify the subsets of abundantly expressed tissue-specific genes. Our dataset provides a baseline for comparison to diseased tissues, as well as a basis for identifying molecular markers of injury to specific organs and tissues, and for anticancer therapy.

## Materials and methods

### Tissue specimens

Normal human tissue specimens were obtained from surgery (for example, the uninvolved regions of resected tumors) or from autopsy, with institutional review board approval. Specimens were frozen on dry ice within 30 minutes of surgical removal or procurement and stored at -80°C. Histological evaluations were performed by H&E staining of frozen sections, and a pathologist (J.H. and/or M.vdR.) reviewed all slides to confirm the anatomical site of origin and histological normalcy (that is, to rule out inflammation, infection, necrosis, malignancy). In total, we selected for study 115 tissue samples representing 35 different human tissues (Additional data file 1). Total RNA was isolated from tissues using TRIzol Reagent (Invitrogen) according to the manufacturer's instruction, and RNA quality was assessed by the integrity of rRNA bands following gel electrophoresis. The poly(A)^+ ^mRNA fraction was then isolated from total RNA using FastTrack2.0 kit (Invitrogen), and quantified by UV spectrophotometry.

### Expression profiling

Gene-expression profiling was performed essentially as reported previously [8], and detailed protocols for array fabrication and hybridization are available online [28]. Briefly, Cy5-labeled cDNA was prepared using 2 μg mRNA from normal tissue samples, and Cy3-labeled cDNA was prepared using 1.5 μg mRNA common reference, pooled from 11 established human cell lines [8]. For each experimental sample, Cy5- and Cy3-labeled samples were co-hybridized to a cDNA microarray containing 39,711 human cDNAs, representing 26,260 different genes (UniGene clusters [21]). For the common reference mRNA (Cy5) versus genomic DNA (Cy3) comparisons, normal female genomic DNA was labeled as described [24]. Following hybridization, microarrays were imaged using an Axon GenePix 4000 scanner (Axon Instruments). Fluorescence ratios for array elements were extracted using GenePix software, and uploaded onto the Stanford Microarray Database (SMD) [29] for subsequent analysis. The complete microarray dataset is accessible from SMD [30], or from the Gene Expression Omnibus [31] (accession number GSE2193).

### Data analysis

Fluorescence ratios were normalized by mean-centering genes for each array (that is, 'global' normalization), and then by mean centering each gene across all arrays. We included for analysis only well-measured genes whose expression varied, as determined by: signal intensity over background more than twofold in either test or reference channels in at least 75% of samples; and a fourfold or more ratio variation from the mean in at least two samples (unless otherwise indicated). Hierarchical clustering was performed and displayed using Cluster and TreeView software [32]. Tissue-selective genes were identified using the two-class (each tissue versus all other tissues) significance analysis of microarrays (SAM) method [25], which utilizes a modified *t*-test statistic and sample-label permutations to evaluate statistical significance. The false-discovery rate (FDR), an estimate of the fraction of falsely called tissue-selective genes, varied by tissue, but in all cases was less than 5% (specific FDRs are listed in Additional data file 3). For tissue-selective genes, only tissue types with two or more samples were considered for analysis, and we only considered genes that were well-measured in more than 50% of the samples for the selected tissue type analyzed. GO annotations were assigned to arrayed genes using the AmiGO browser [33] to select relevant GO annotations, and the 'loc2go' file [34] to identify the corresponding sets of genes. Transcript abundance was estimated by multiplying (for each gene) the ratio of tissue sample mRNA versus common reference mRNA by the ratio (average ratio from triplicate experiments) of common reference mRNA versus normal female genomic DNA. Highly-abundant tissue specific transcripts were defined for each tissue type as the top (capped at 50 genes) tissue specific transcripts, identified using the SAM method, from the 1,000 most abundantly expressed transcripts in the full dataset.

## Additional data files

The following additional data are available with the online version of this paper. Additional data file 1 is a table listing the normal tissue specimens included in microarray analysis. Additional data file 2 is a table listing the variably expressed genes. Additional data file 3 is a table listing tissue-specific transcripts. Additional data file 4 is a table listing functionally annotated gene sets. Additional data file 5 is a table listing highly abundant tissue-specific transcripts. Additional data file 6 is a figure showing tissue-specific gene expression. Additional data file 7 is a figure showing expression of functionally annotated gene sets. Additional data file 8 is a figure showing highly abundant tissue-specific gene expression.

## Supplementary Material

Additional File 1 A table listing the normal tissue specimens included in microarray analysis Click here for file

Additional File 2 A table listing the variably expressed genes. **Sheet 1**: Dataset represented in Fig. 1, which includes well-measured genes in ≥ 75% of samples, and with ≥ 4-fold ratio variation from the mean in at least 2 samples; samples ordered by clustering.**Sheet 2**: Variably expressed genes which are well-measured in ≥ 75% of samples, with ≥ 2-fold ratio variation from the mean in at least 2 samples; samples ordered by anatomic site. **Sheet 3**: Variably expressed genes which are well-measured in ≥ 25% of samples, with ≥ 4-fold ratio variation from the mean in at least 2 samples; samples ordered by anatomic site. **Sheet 4**: Same dataset as sheet 1, but here ratios represent relative transcript abundance (See Materials and methods). **Sheet 5**: Same dataset as sheet 2, but here ratios represent relative transcript abundance (See Materials and methods). **Sheet 6**: Same dataset as sheet 3, but here ratios represent relative transcript abundance (See Materials and methods) Click here for file

Additional File 3 A table listing tissue-specific transcripts Click here for file

Additional File 4 A table listing functionally annotated gene sets Click here for file

Additional File 5 A table listing highly abundant tissue-specific transcripts Click here for file

Additional File 6 A figure showing tissue-specific gene expression. Variably-expressed genes determined to be expressed in a tissue-selective fashion using the SAM method are depicted as described in the legend to manuscript Figure 2. **a**, brain; **b**, salivary gland; **c**, esophagus; **d**, stomach; **e**, small bowel; **f**, colon; **g**, pancreas; **h**, liver; **i**, heart; **j**, skeletal muscle; **k**, lung; **l**, kidney; **m**, bladder;**n**, prostate; **o**, seminal vesicle; **p**, testis; **q**, ovary; **r**, fallopian tube; **s**, uterus; **t**, cervix, **u**, thyroid; **v**, parathyroid; **w**, adrenal; **x**, lymph node; **y**, tonsil; **z**, thymus; **aa**, spleen; **bb**, buffy coat Click here for file

Additional File 7 A figure showing expression of functionally annotated gene sets. Hierarchical cluster of 115 normal tissue specimens and annotated gene sets representing examples of specific molecular functions, cellular components, or biological processes. **a**, tyrosine kinase (activity); **b**, kinase (activity); **c**, G-protein coupled receptor (activity); **d**, transcription factor activity; **e**, ion channel (activity); **f**, extracellular matrix (component), **g**, cell adhesion (process); **h**, programmed cell death (process) Click here for file

Additional File 8 A figure showing highly abundant tissue-specific gene expression. Highly-abundant tissue specific transcripts were defined for each tissue type as the top (capped at 50 genes) tissue specific transcripts, identified using the SAM method, from the 1000 most abundantly expressed transcripts in the full dataset. **a**, brain; **b**, salivary gland; **c**, esophagus; **d**, stomach; **e**, small bowel; **f**, colon; **g**, pancreas; **h**, liver; **i**, heart; **j**, skeletal muscle; **k**, lung; **l**, kidney; **m**, bladder;**n**, prostate; **o**, seminal vesicle; **p**, testis; **q**, ovary; **r**, fallopian tube; **s**, uterus; **t**, cervix, **u**, thyroid; **v**, parathyroid; **w**, adrenal; **x**, lymph node; **y**, tonsil; **z**, thymus; **aa**, spleen; **bb**, buffy coat Click here for file
