# Supplementary material for: A DNA microarray survey of gene expression in normal human tissues
Source: Genome Biol. 2005 Feb 14;6(3):R22. doi: 10.1186/gb-2005-6-3-r22 (PMC1088941; doi:10.1186/gb-2005-6-3-r22)

a. Brain

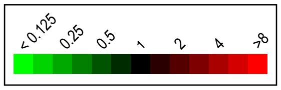

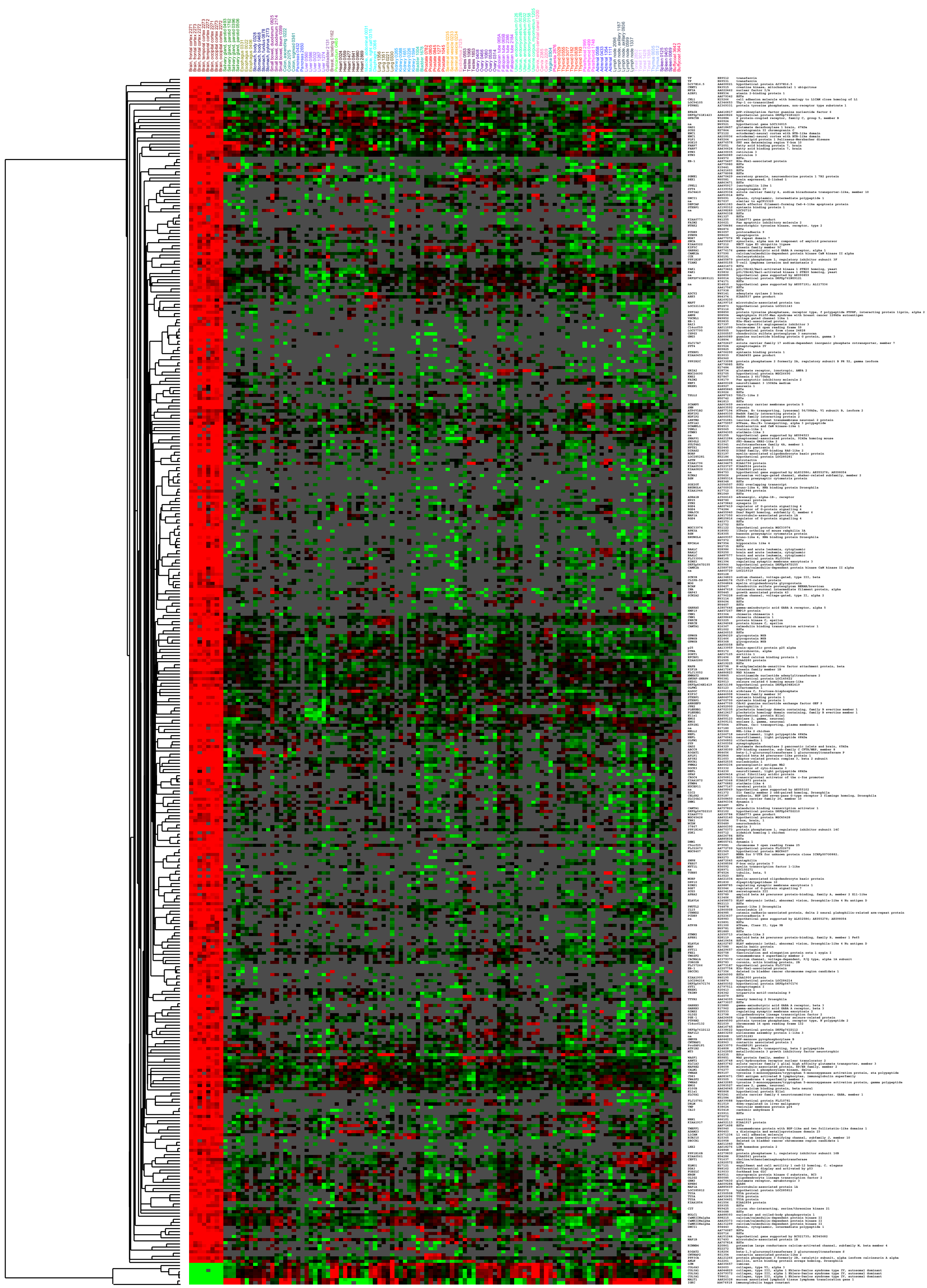

b. Salivary gland

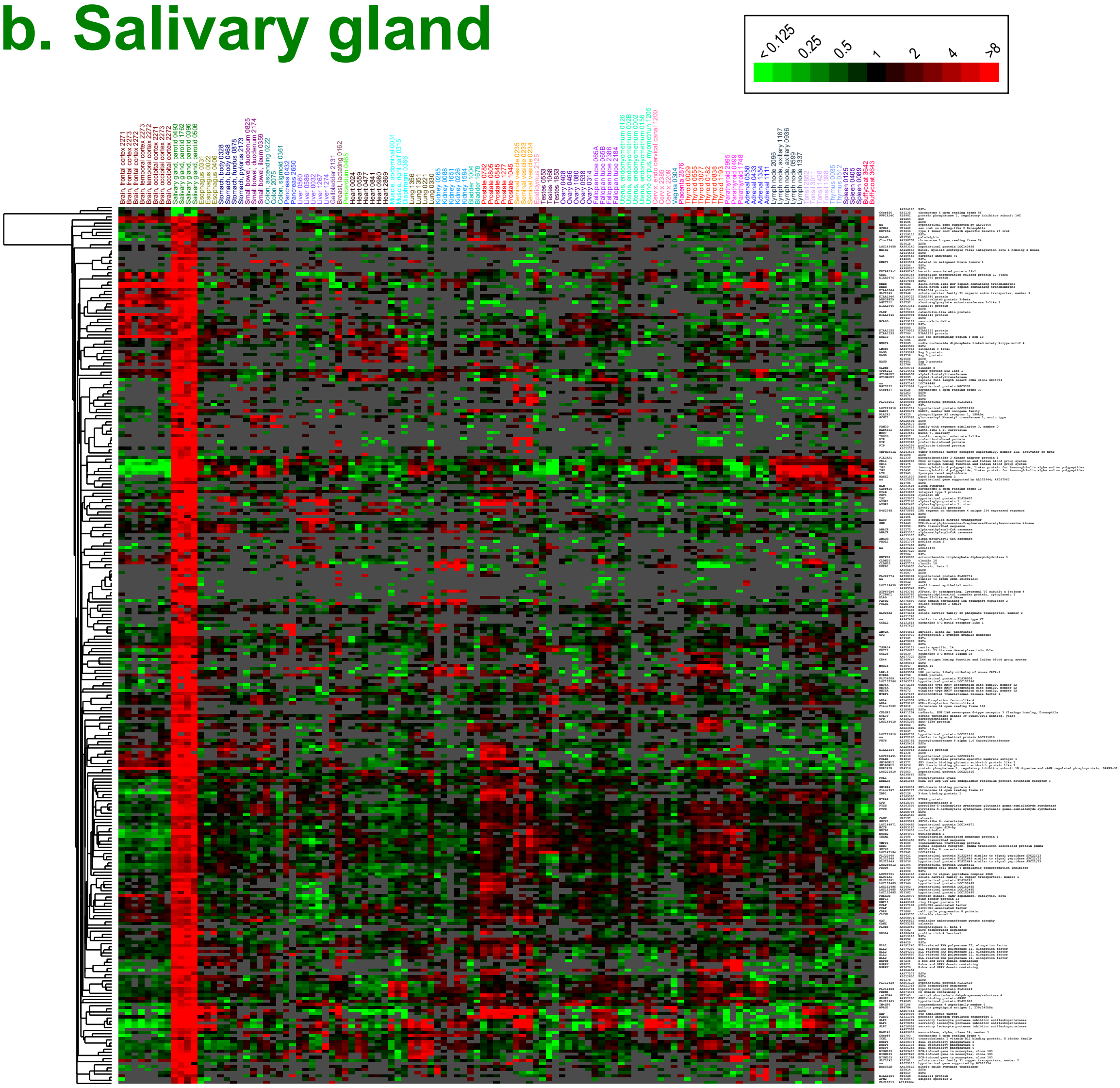

c. Esophagus

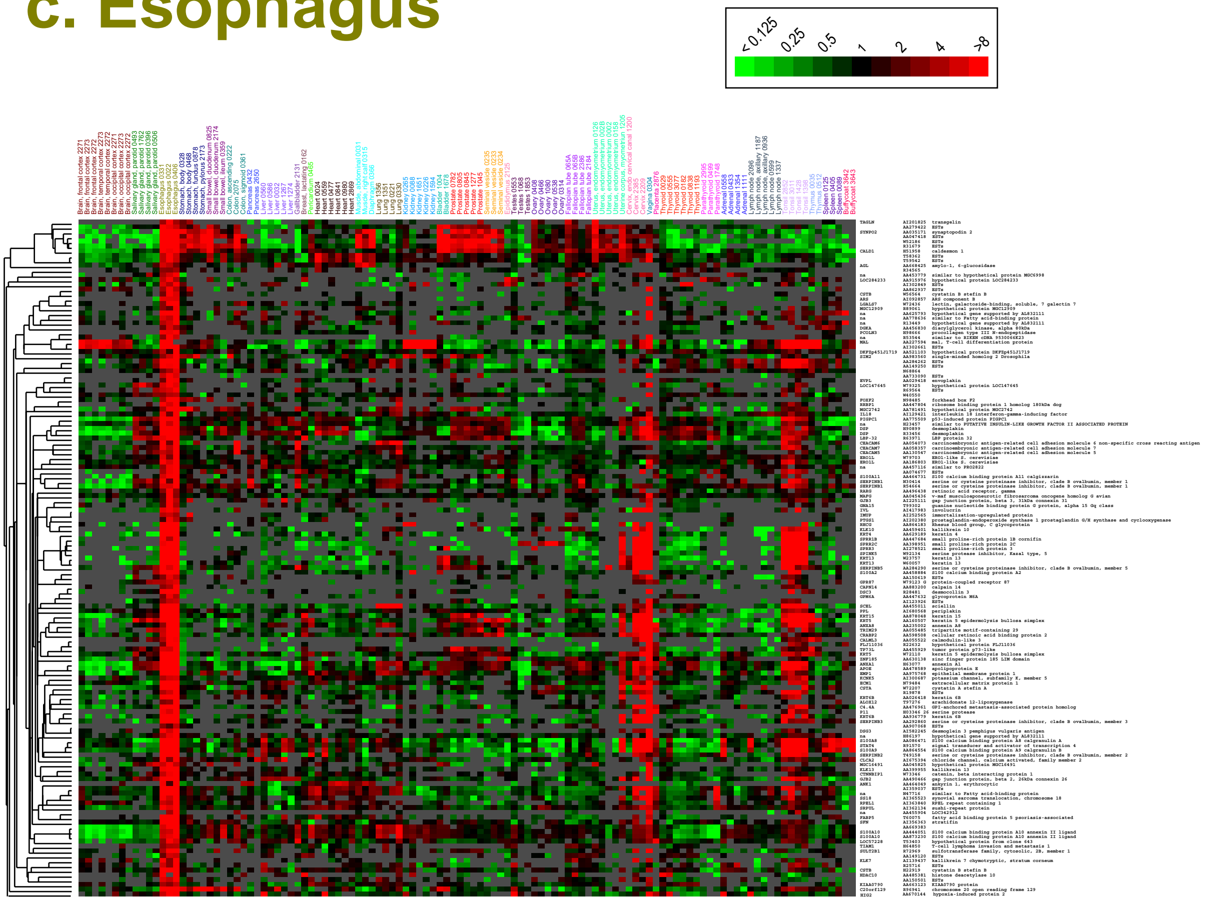

d. Stomach

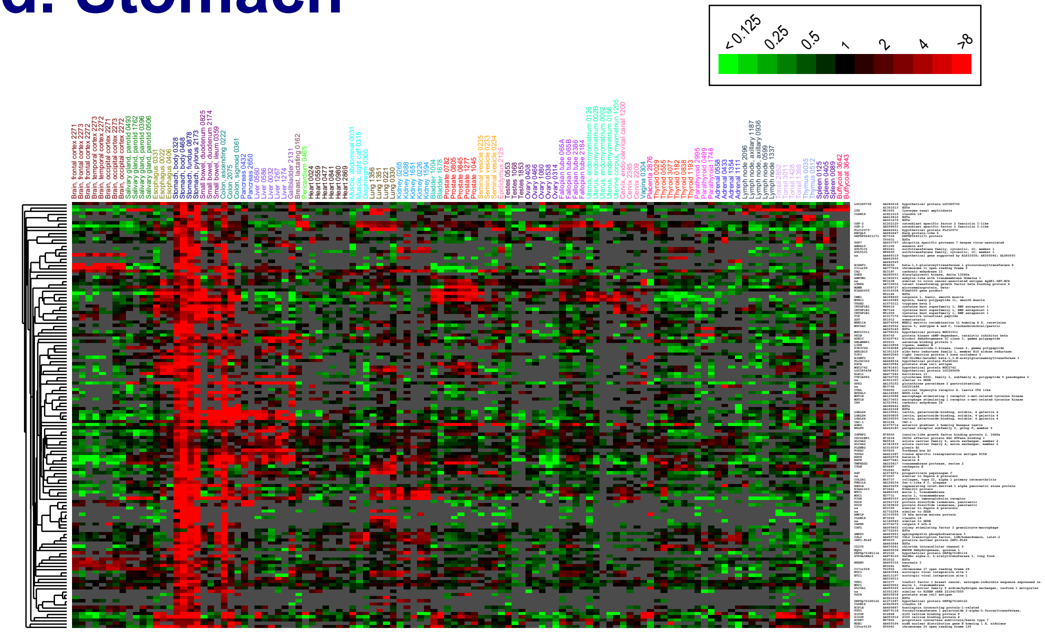

e. Small bowel

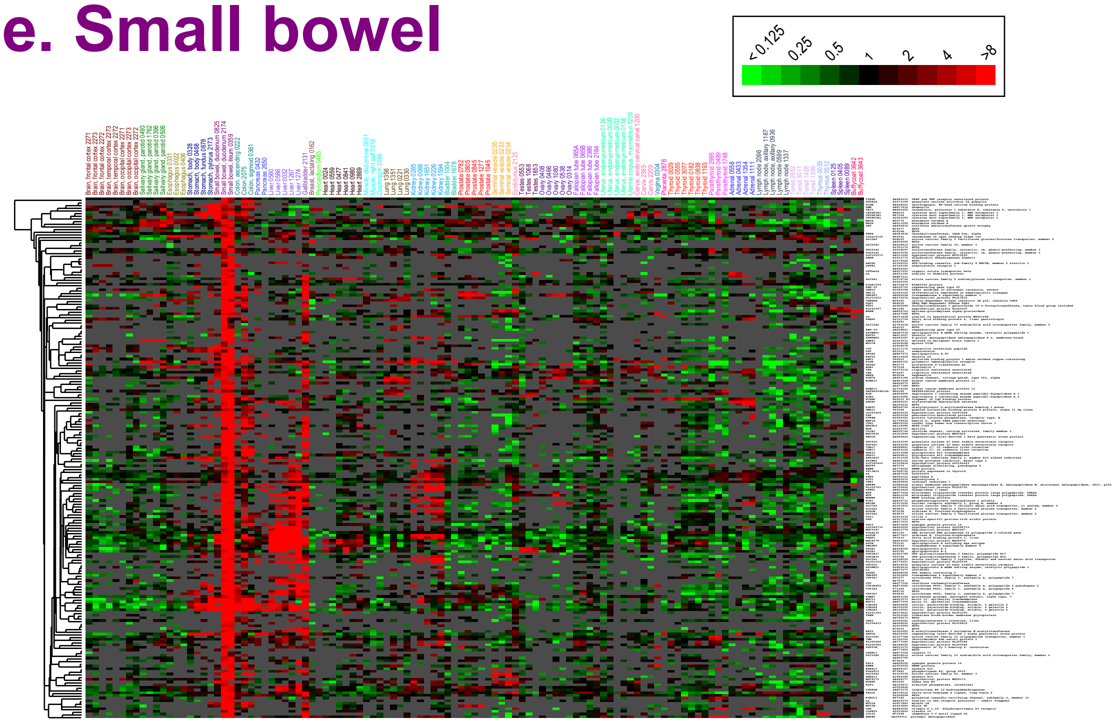

f. Colon

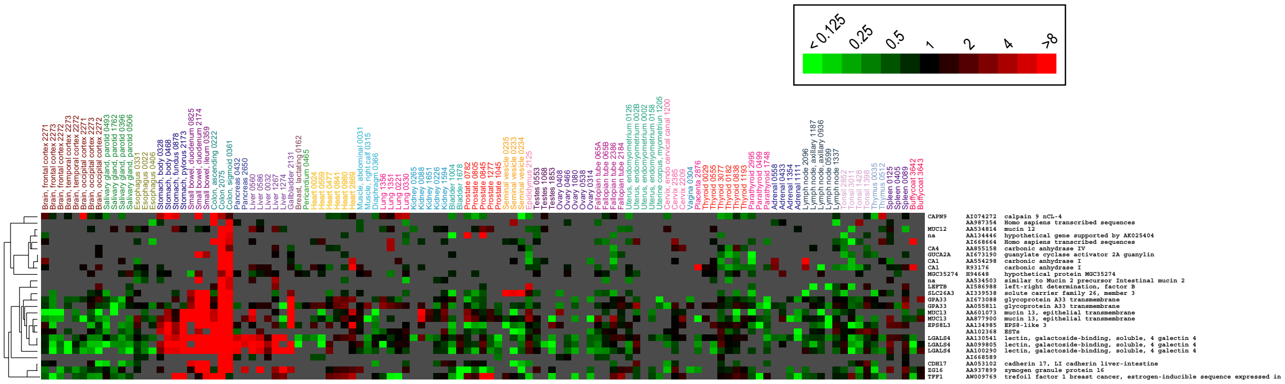

< 0.125
0.25
0.5
1
2
4
>8
CAPN9 AI074272 calpain 9 nCL-4
AA987354 Homo sapiens transcribed sequences
MUC12 AA534814 mucin 12
na AA134446 hypothetical gene supported by AK025404
AI668664 Homo sapiens transcribed sequences
CA4 AA855158 carbonic anhydrase IV
GUCA2A AI673190 guanylate cyclase activator 2A guanylin
CA1 AA554298 carbonic anhydrase I
CA1 R93176 carbonic anhydrase I
MGC35274 H94648 hypothetical protein MGC35274
na AA534503 similar to Mucin 2 precursor Intestinal mucin 2
LEFTB AI586988 left-right determination, factor B
SLC26A3 AI339538 solute carrier family 26, member 3
GPA33 AI673088 glycoprotein A33 transmembrane
GPA33 AA055811 glycoprotein A33 transmembrane
MUC13 AA601073 mucin 13, epithelial transmembrane
MUC13 AA877900 mucin 13, epithelial transmembrane
EPS8L3 AA134985 EPS8-like 3
AA102368 ESTs
LGALS4 AA130541 lectin, galactoside-binding, soluble, 4 galectin 4
LGALS4 AA099805 lectin, galactoside-binding, soluble, 4 galectin 4
LGALS4 AA100290 lectin, galactoside-binding, soluble, 4 galectin 4
AI668589
CDH17 AA053102 cadherin 17, LI cadherin liver-intestine
ZG16 AA937899 zymogen granule protein 16
TFF1 AW009769 trefoil factor 1 breast cancer, estrogen-inducible sequence expressed in

g. Pancreas

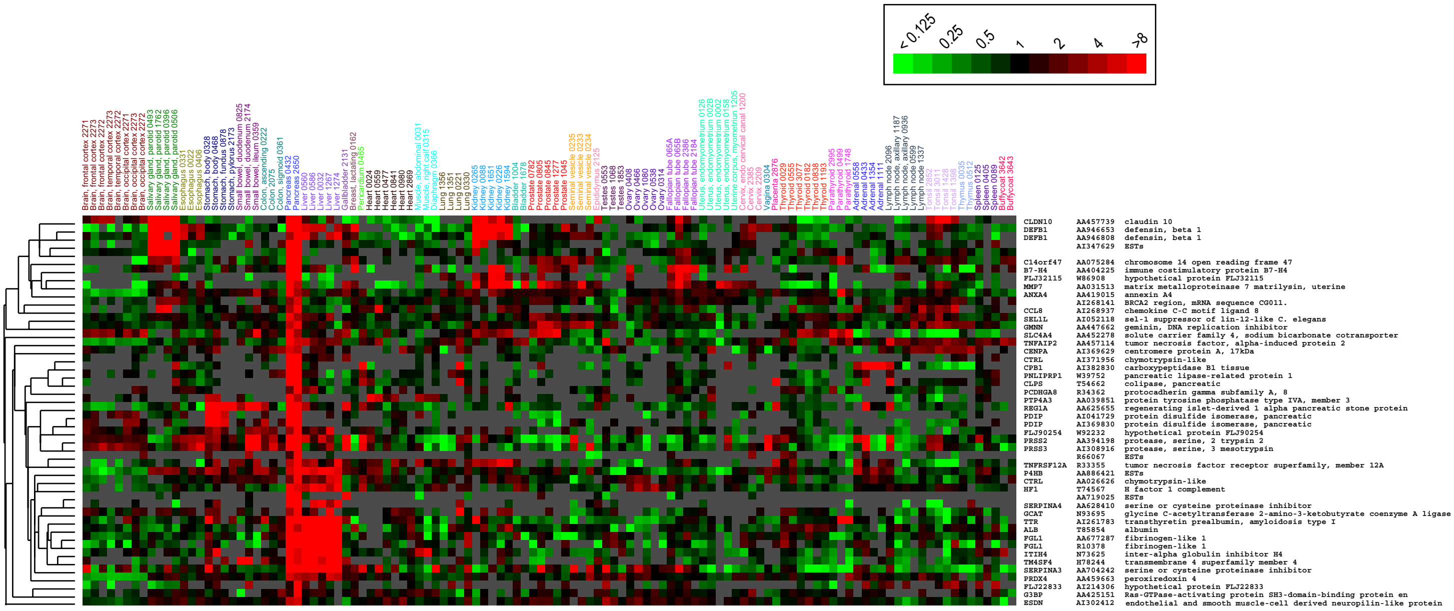
< 0.125
0.25
0.5
1
2
4
>8
CLDN10 AA457739 claudin 10
DEFB1 AA946653 defensin, beta 1
DEFB1 AA946808 defensin, beta 1
AI347629 ESTs
C14orf47 AA075284 chromosome 14 open reading frame 47
B7-H4 AA404225 immune costimulatory protein B7-H4
FLJ32115 W86908 hypothetical protein FLJ32115
MMP7 AA031513 matrix metalloproteinase 7 matrilysin, uterine
ANXA4 AA419015 annexin A4
AI268141 BRCA2 region, mRNA sequence CG011.
CCL8 AI268937 chemokine C-C motif ligand 8
SEL1L AI052118 sel-1 suppressor of lin-12-like C. elegans
GMNN AA447662 geminin, DNA replication inhibitor
SLC4A4 AA452278 solute carrier family 4, sodium bicarbonate cotransporter
TNFAIP2 AA457114 tumor necrosis factor, alpha-induced protein 2
CENPA AI369629 centromere protein A, 17kDa
CTRL AI371956 chymotrypsin-like
CPB1 AI382830 carboxypeptidase B1 tissue
PNLIPRP1 W39752 pancreatic lipase-related protein 1
CLPS T54662 colipase, pancreatic
PCDHGA8 R34362 protocadherin gamma subfamily A, 8
PTP4A3 AA039851 protein tyrosine phosphatase type IVA, member 3
REG1A AA625655 regenerating islet-derived 1 alpha pancreatic stone protein
PDIP AI041729 protein disulfide isomerase, pancreatic
PDIP AI369830 protein disulfide isomerase, pancreatic
FLJ90254 W92232 hypothetical protein FLJ90254
PRSS2 AA394198 protease, serine, 2 trypsin 2
PRSS3 AI308916 protease, serine, 3 mesotrypsin
R66067 ESTs
TNFRSF12A R33355 tumor necrosis factor receptor superfamily, member 12A
P4HB AA886421 ESTs
CTRL AA026626 chymotrypsin-like
HF1 T74567 H factor 1 complement
AA719025 ESTs
SERPINA4 AA628410 serine or cysteine proteinase inhibitor
GCAT N93695 glycine C-acetyltransferase 2-amino-3-ketobutyrate coenzyme A ligase
TTR AI261783 transthyretin prealbumin, amyloidosis type I
ALB T85854 albumin
FGL1 AA677287 fibrinogen-like 1
FGL1 R10378 fibrinogen-like 1
ITIH4 N73625 inter-alpha globulin inhibitor H4
TM4SF4 H78244 transmembrane 4 superfamily member 4
SERPINA3 AA704242 serine or cysteine proteinase inhibitor
PRDX4 AA459663 peroxiredoxin 4
FLJ22833 AI214306 hypothetical protein FLJ22833
G3BP AA425151 Ras-GTPase-activating protein SH3-domain-binding protein en
ESDN AI302412 endothelial and smooth muscle-cell derived neuropilin-like protein

h. Liver

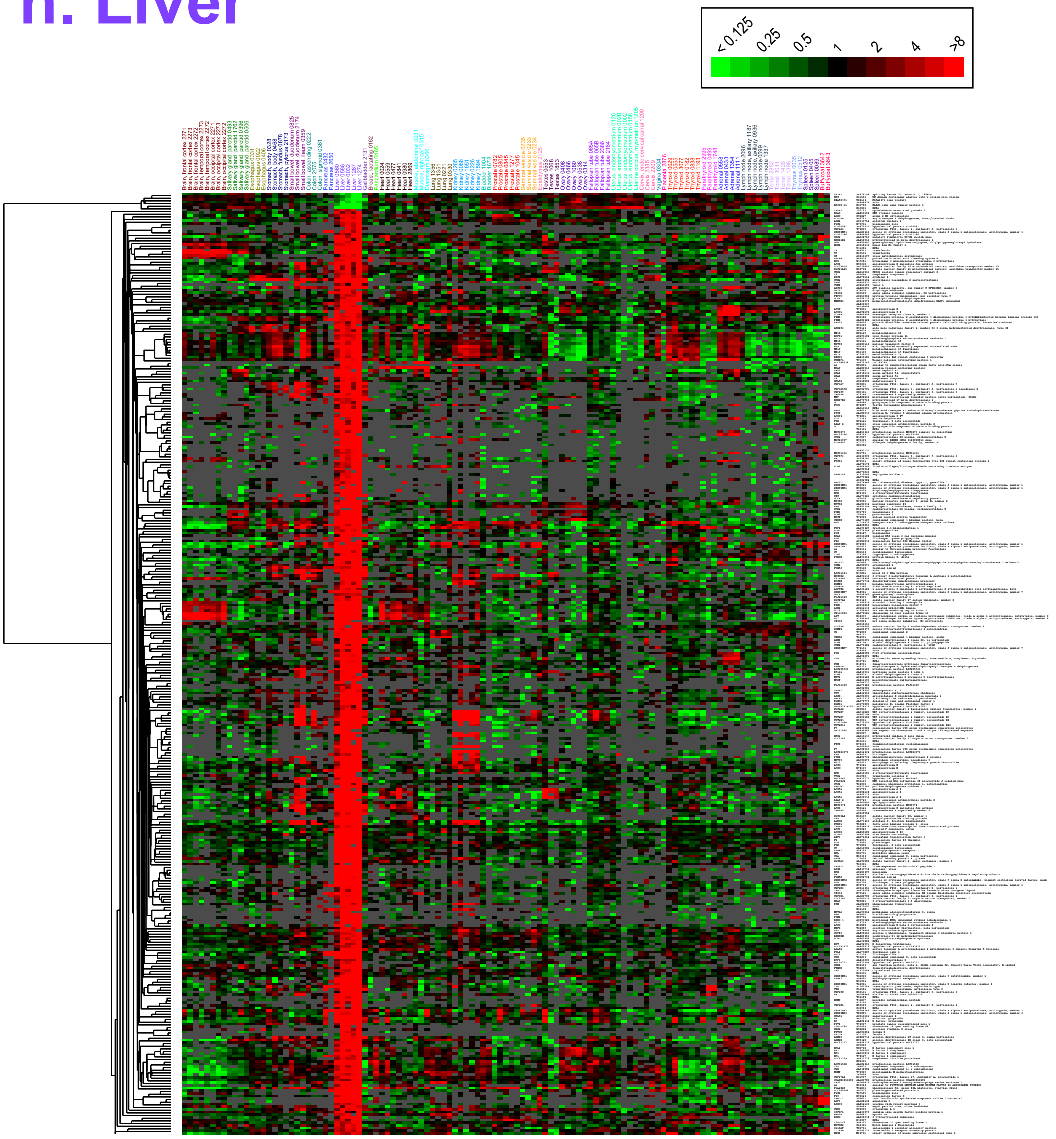

i. Heart

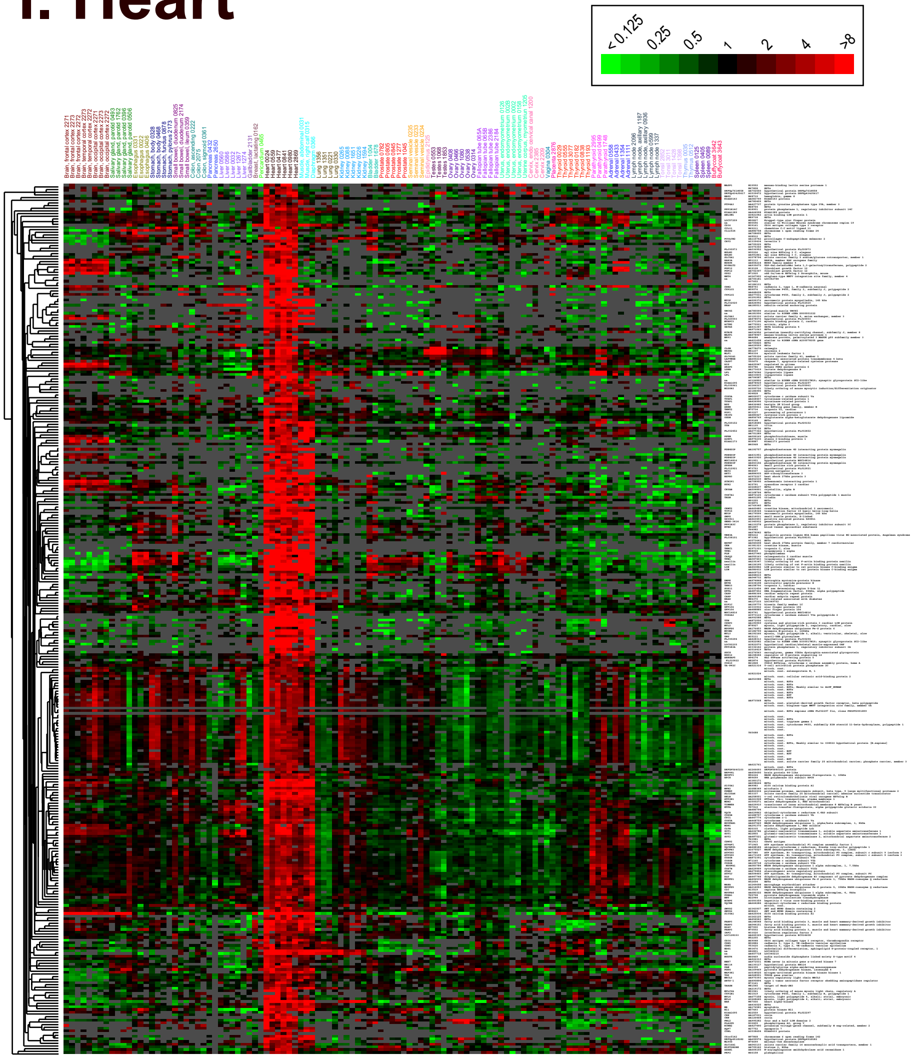

j. Skeletal muscle

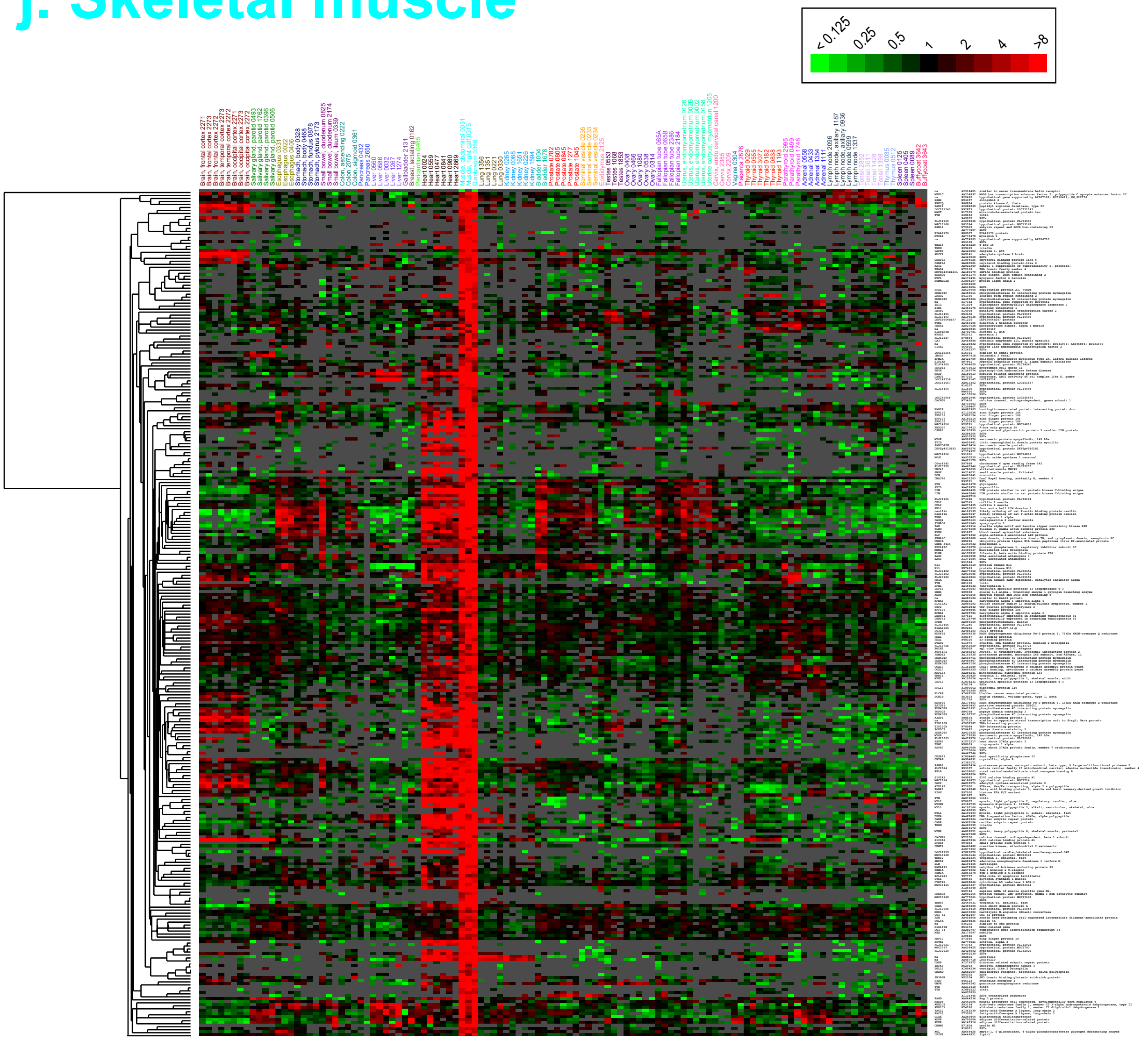

k. Lung

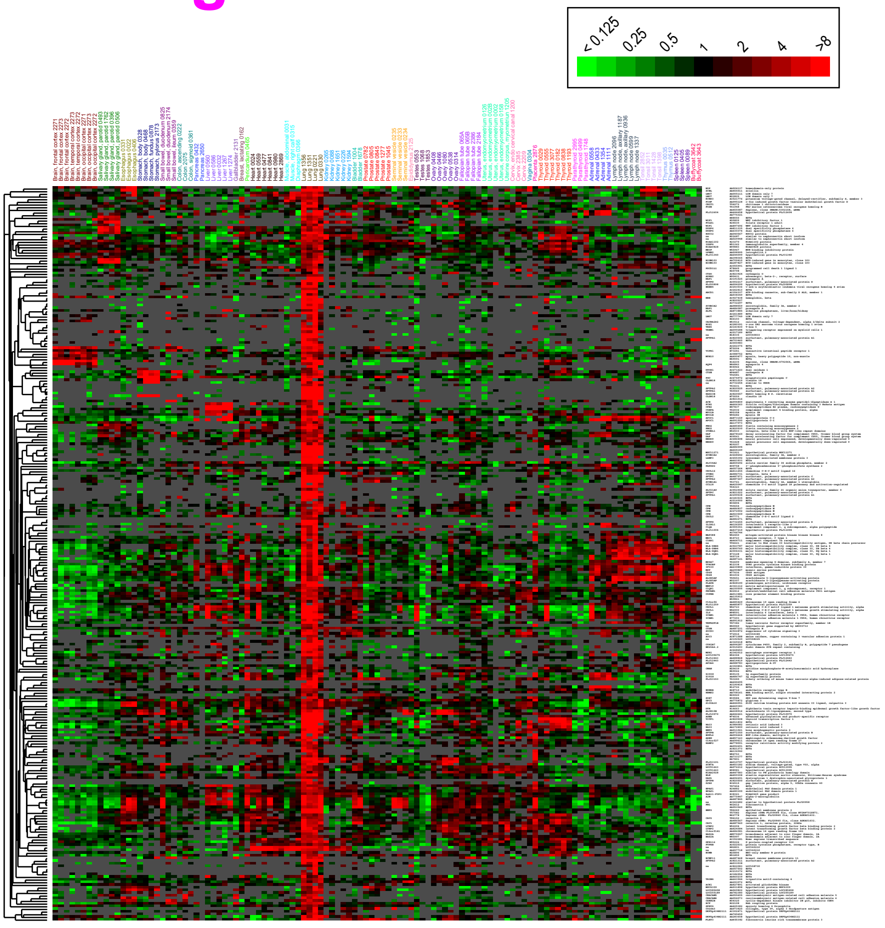

I. Kidney

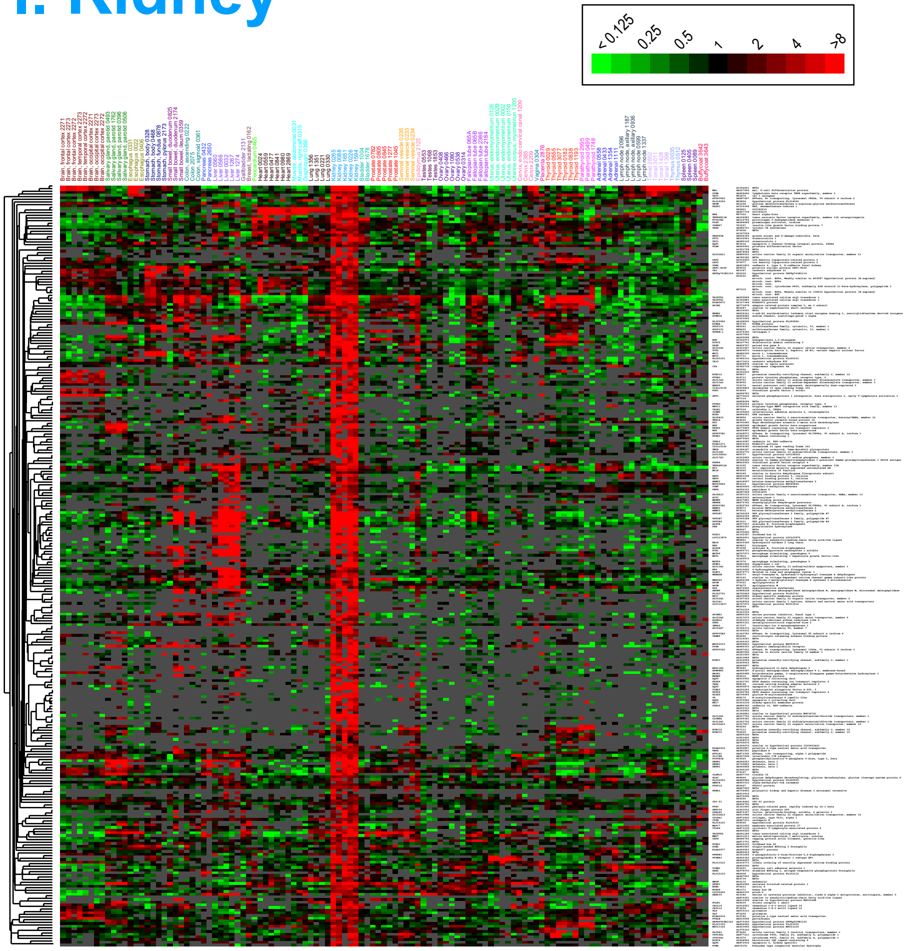

m. Bladder

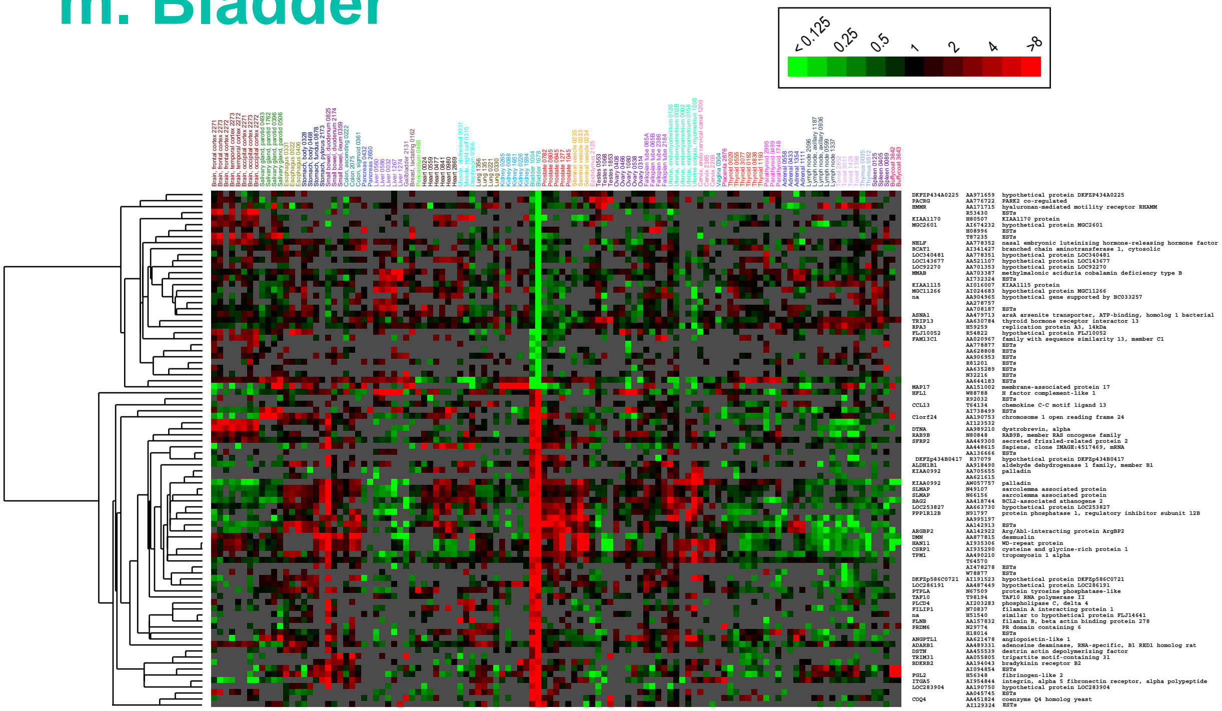

n. Prostate

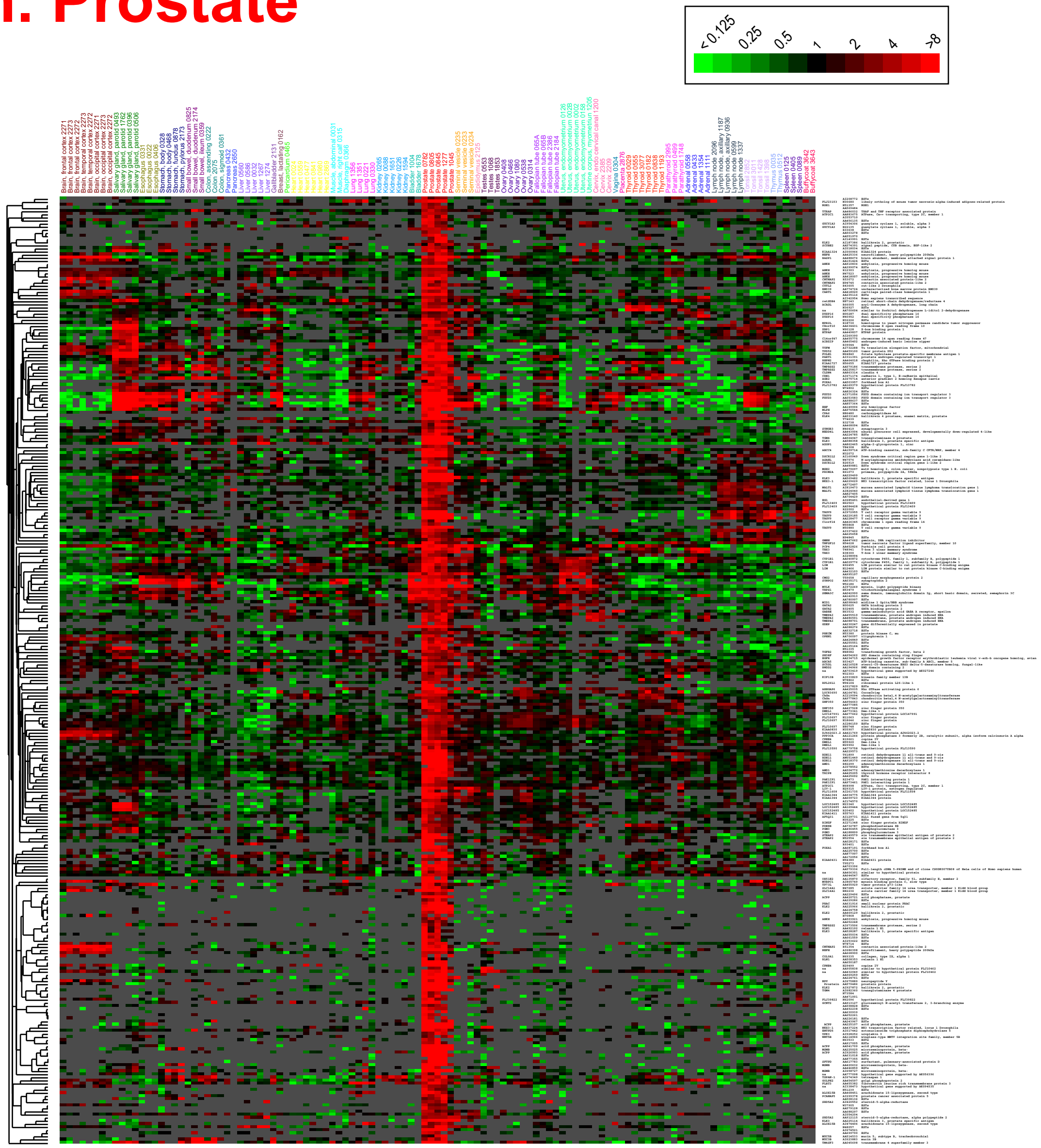

o. Seminal vesicle

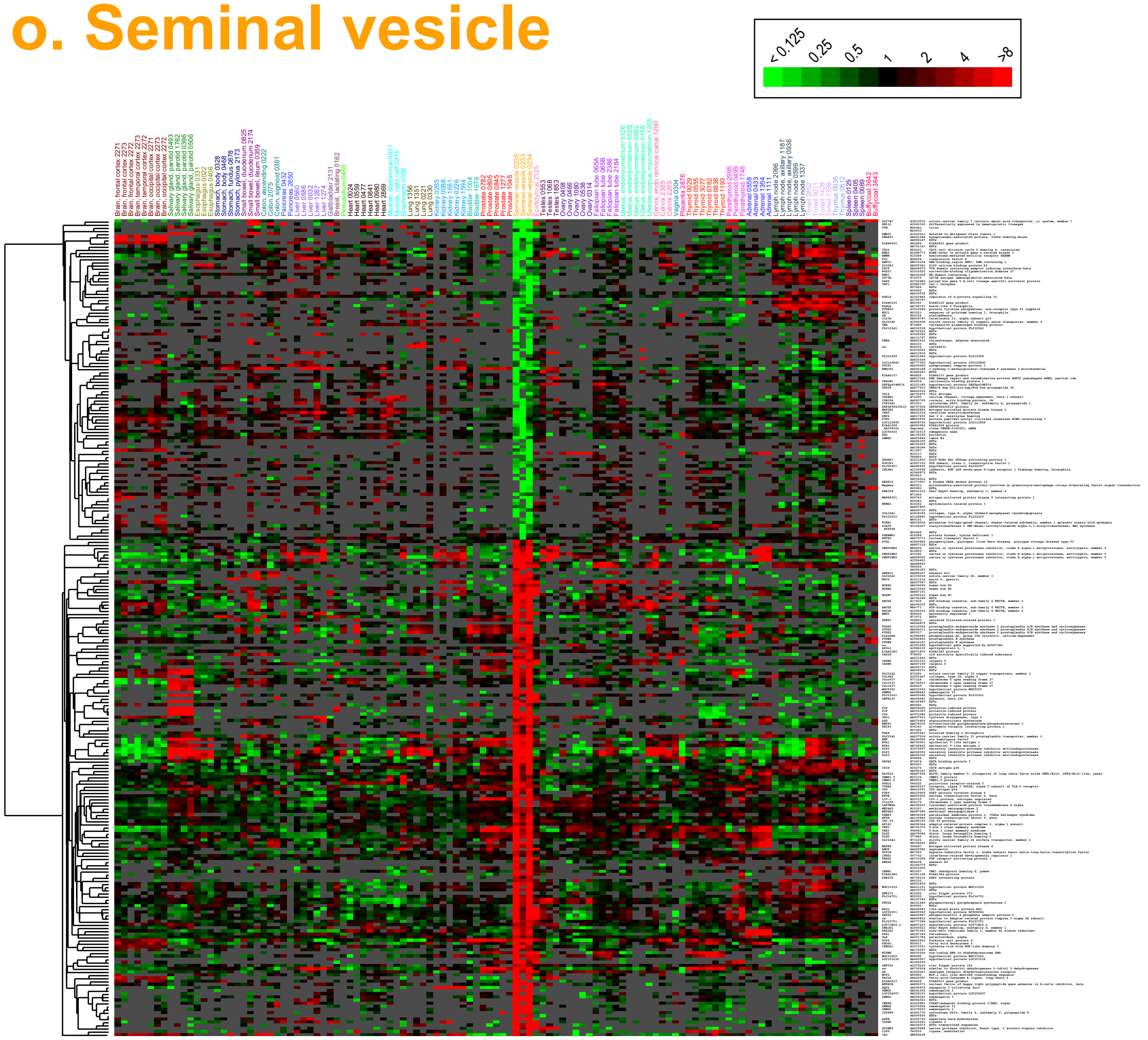

p. Testis

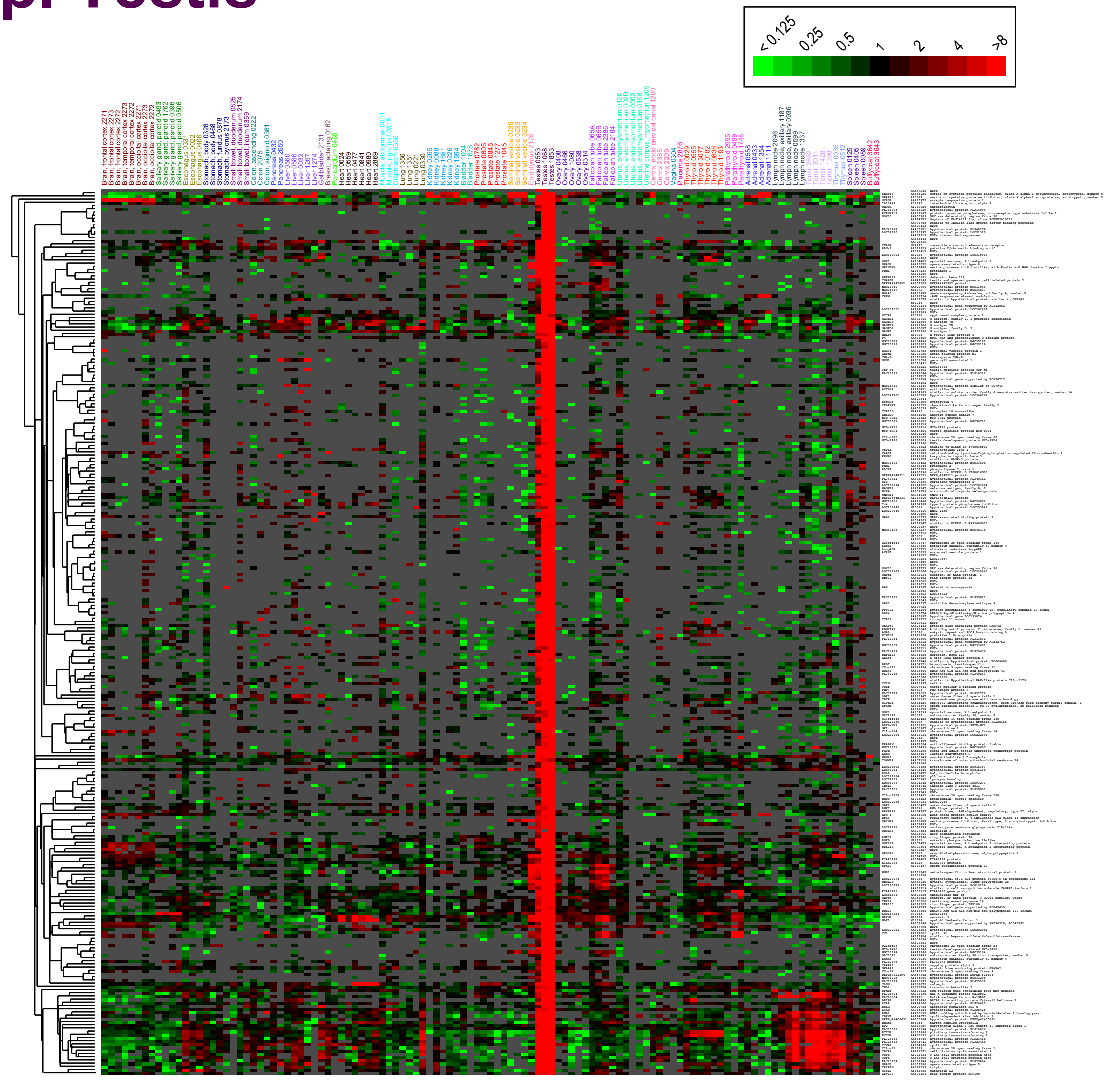

q. Ovary

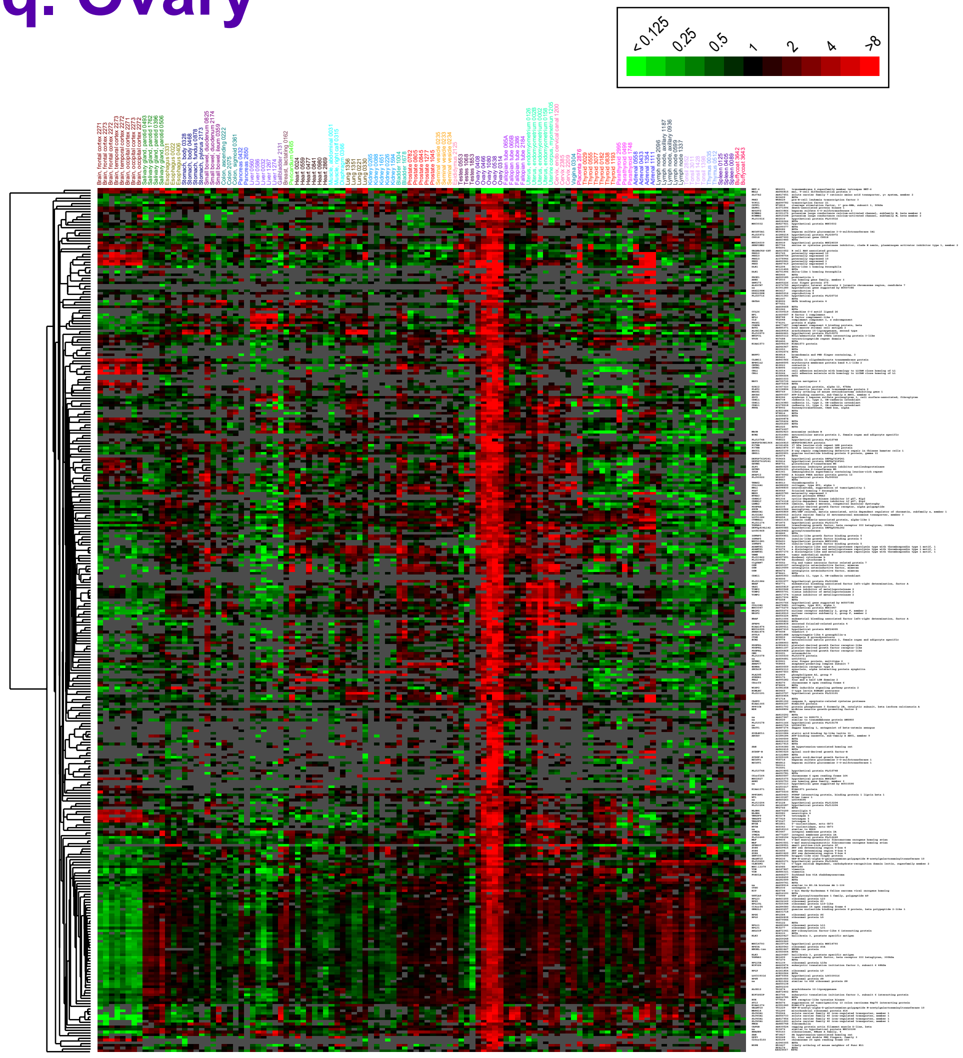

r. Fallopian tube

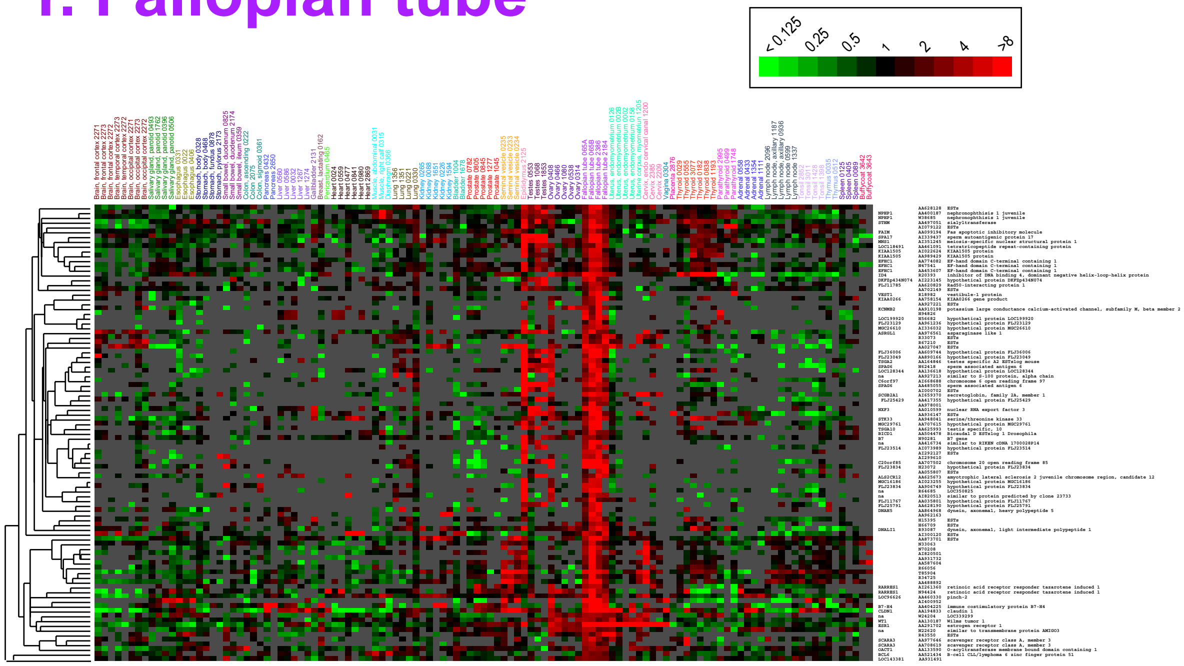

s. Uterus

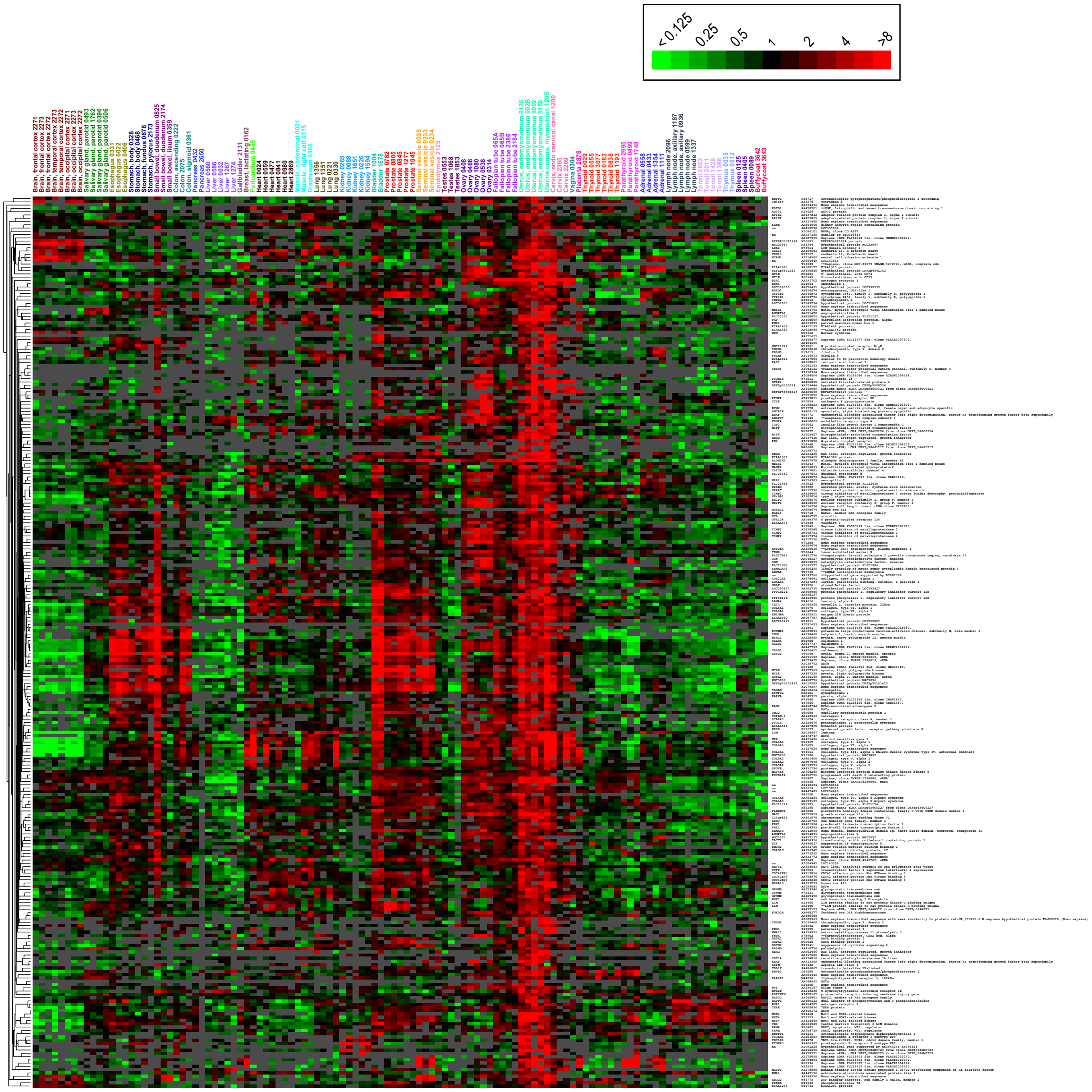

t. Cervix

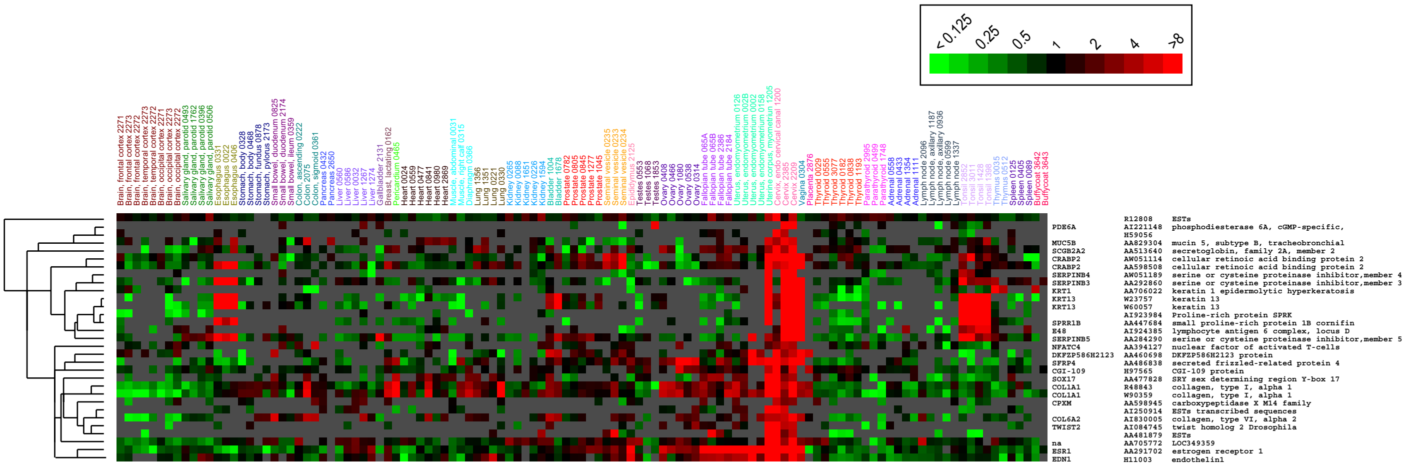
< 0.125
0.25
0.5
1
2
4
>8
R12808 ESTs
PDE6A AI221148 phosphodiesterase 6A, cGMP-specific,
H59056
MUC5B AA829304 mucin 5, subtype B, tracheobronchial
SCGB2A2 AA513640 secretoglobin, family 2A, member 2
CRABP2 AW051114 cellular retinoic acid binding protein 2
CRABP2 AA598508 cellular retinoic acid binding protein 2
SERPINB4 AW051189 serine or cysteine proteinase inhibitor,member 4
SERPINB3 AA292860 serine or cysteine proteinase inhibitor,member 3
KRT1 AA706022 keratin 1 epidermolytic hyperkeratosis
KRT13 W23757 keratin 13
KRT13 W60057 keratin 13
AI923984 Proline-rich protein SPRK
SPRR1B AA447684 small proline-rich protein 1B cornifin
E48 AI924385 lymphocyte antigen 6 complex, locus D
SERPINB5 AA284290 serine or cysteine proteinase inhibitor,member 5
NFATC4 AA394127 nuclear factor of activated T-cells
DKFZP586H2123 AA460698 DKFZP586H2123 protein
SFRP4 AA486838 secreted frizzled-related protein 4
CGI-109 H97565 CGI-109 protein
SOX17 AA477828 SRY sex determining region Y-box 17
COL1A1 R48843 collagen, type I, alpha 1
COL1A1 W90359 collagen, type I, alpha 1
CPXM AA598945 carboxypeptidase X M14 family
AI250914 ESTs transcribed sequences
COL6A2 AI830005 collagen, type VI, alpha 2
TWIST2 AI084745 twist homolog 2 Drosophila
AA481879 ESTs
na AA705772 LOC349359
ESR1 AA291702 estrogen receptor 1
EDN1 H11003 endothelin1

u. Thyroid

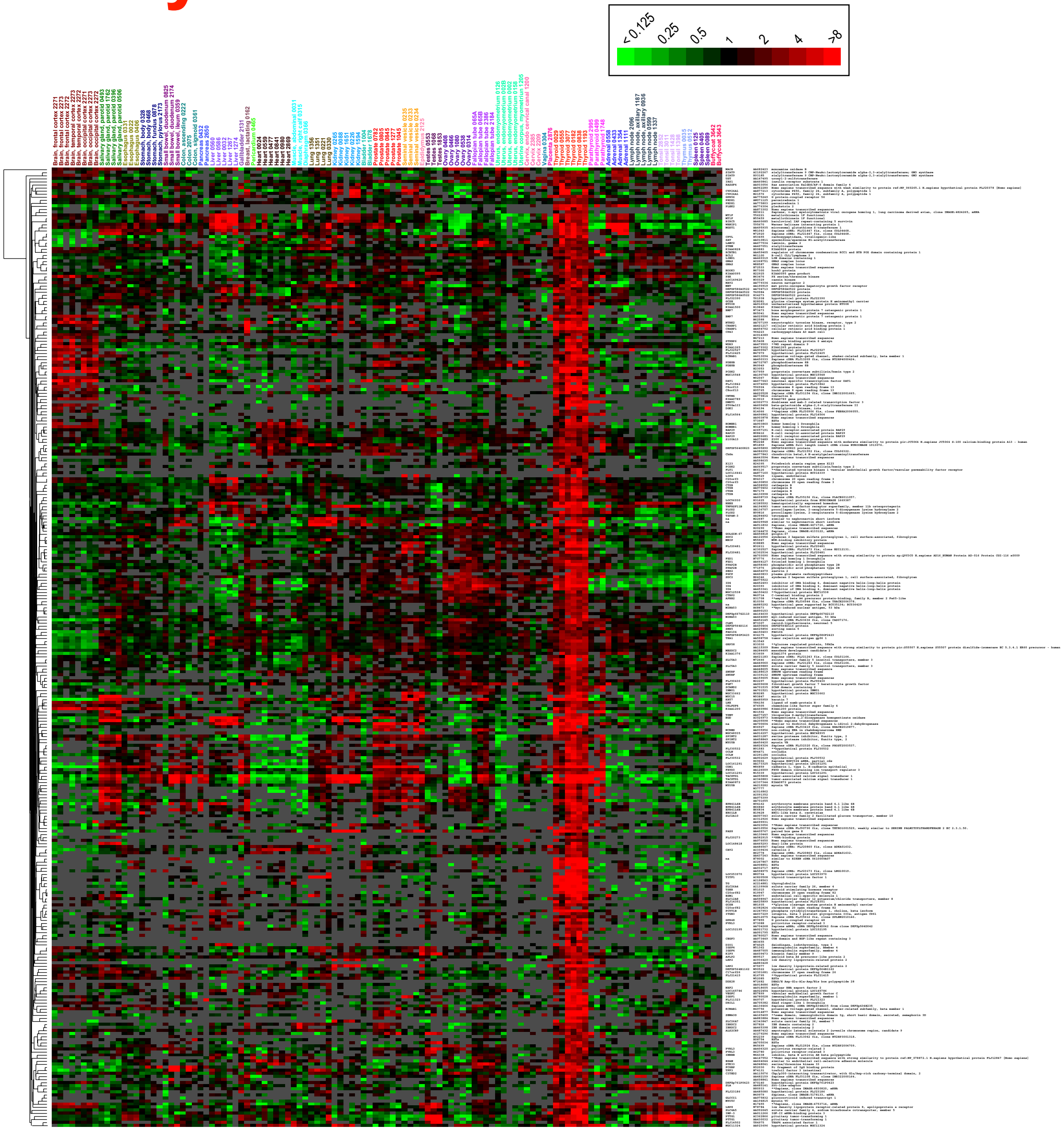

<0.125
0.25
0.5
1
2
4
>8
Brain, frontal cortex 2271
Brain, frontal cortex 2273
Brain, frontal cortex 2272
Brain, temporal cortex 2273
Brain, temporal cortex 2272
Brain, occipital cortex 2271
Brain, occipital cortex 2273
Brain, occipital cortex 2272
Salivary gland, parotid 0493
Salivary gland, parotid 1762
Salivary gland, parotid 0396
Salivary gland, parotid 0506
Esophagus 0331
Esophagus 0022
Esophagus 0406
Stomach, body 0328
Stomach, body 0468
Stomach, fundus 0878
Stomach, pylorus 2173
Small bowel, duodenum 0825
Small bowel, duodenum 2174
Small bowel, ileum 0359
Colon, ascending 0222
Colon 2075
Colon, sigmoid 0361
Pancreas 0432
Pancreas 2650
Liver 0560
Liver 0586
Liver 0032
Liver 1267
Liver 1274
Gallbladder 2131
Breast, lactating 0162
Pericardium 0465
Heart 0024
Heart 0559
Heart 0477
Heart 0841
Heart 0980
Heart 2869
Muscle, abdominal 0031
Muscle, right calf 0315
Diaphragm 0366
Lung 1356
Lung 1351
Lung 0221
Lung 0330
Kidney 0265
Kidney 0088
Kidney 1651
Kidney 0226
Kidney 1594
Bladder 1004
Bladder 1678
Prostate 0782
Prostate 0805
Prostate 0845
Prostate 1277
Prostate 1045
Seminal vesicle 0235
Seminal vesicle 0233
Seminal vesicle 0234
Epididymus 2125
Testes 0553
Testes 1068
Testes 1853
Ovary 0408
Ovary 0466
Ovary 1080
Ovary 0538
Ovary 0314
Fallopian tube 065A
Fallopian tube 065B
Fallopian tube 2386
Fallopian tube 2184
Uterus, endomyometrium 0126
Uterus, endomyometrium 002B
Uterus, endomyometrium 0002
Uterus, endomyometrium 0158
Uterine corpus, myometriun 1205
Cervix, endo cervical canal 1200
Cervix 2385
Cervix 2209
Vagina 0304
Placenta 2876
Thyroid 0029
Thyroid 0555
Thyroid 3077
Thyroid 0182
Thyroid 0838
Thyroid 1193
Parathyroid 2995
Parathyroid 0499
Parathyroid 1748
Adrenal 0558
Adrenal 0433
Adrenal 1354
Adrenal 1111
Lymph node 2096
Lymph node, axillary 1187
Lymph node, axillary 0936
Lymph node 0599
Lymph node 1337
Tonsil 2852
Tonsil 3011
Tonsil 1428
Tonsil 1398
Thymus 0035
Thymus 0512
Spleen 0125
Spleen 0405
Spleen 0089
Buffycoat 3642
Buffycoat 3643

v. Parathyroid

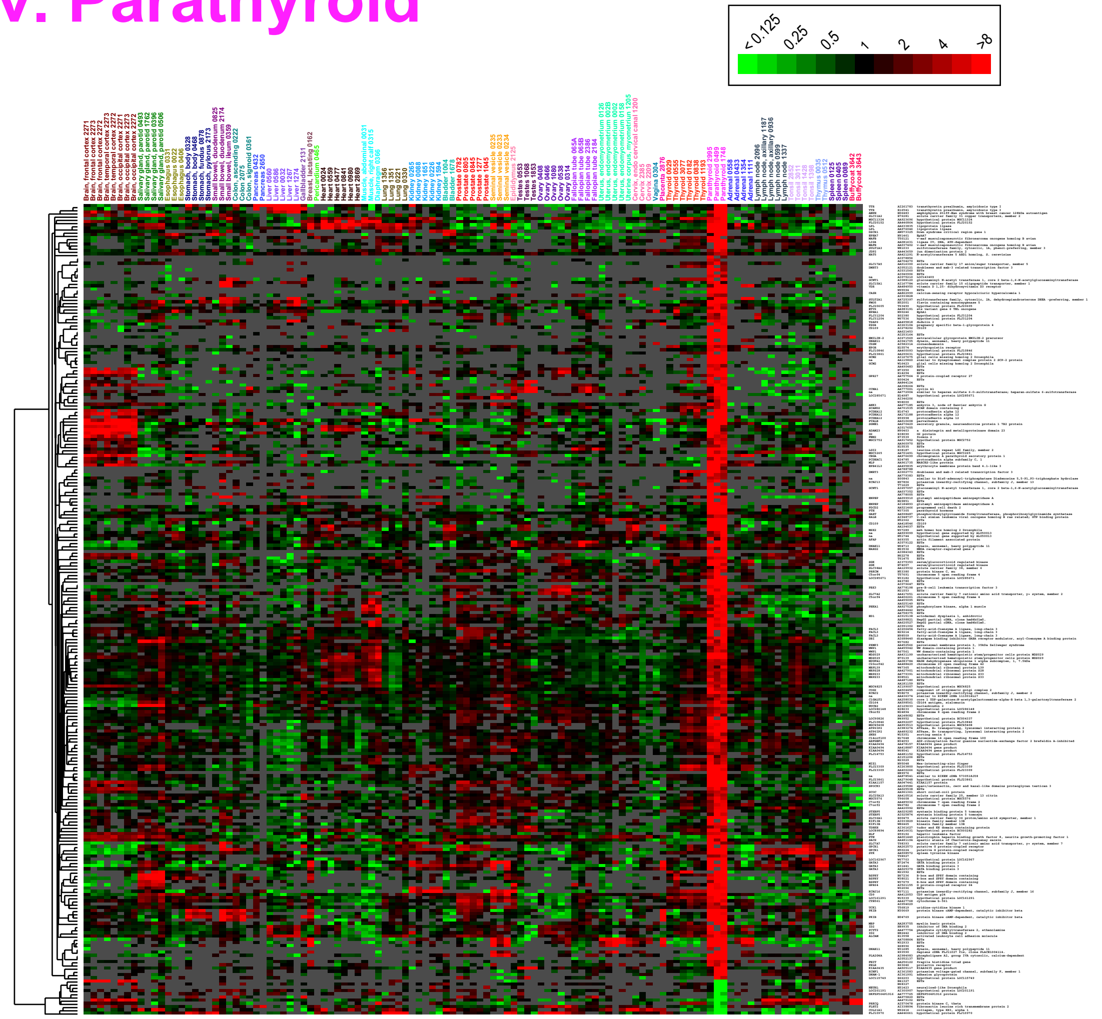

w. Adrenal

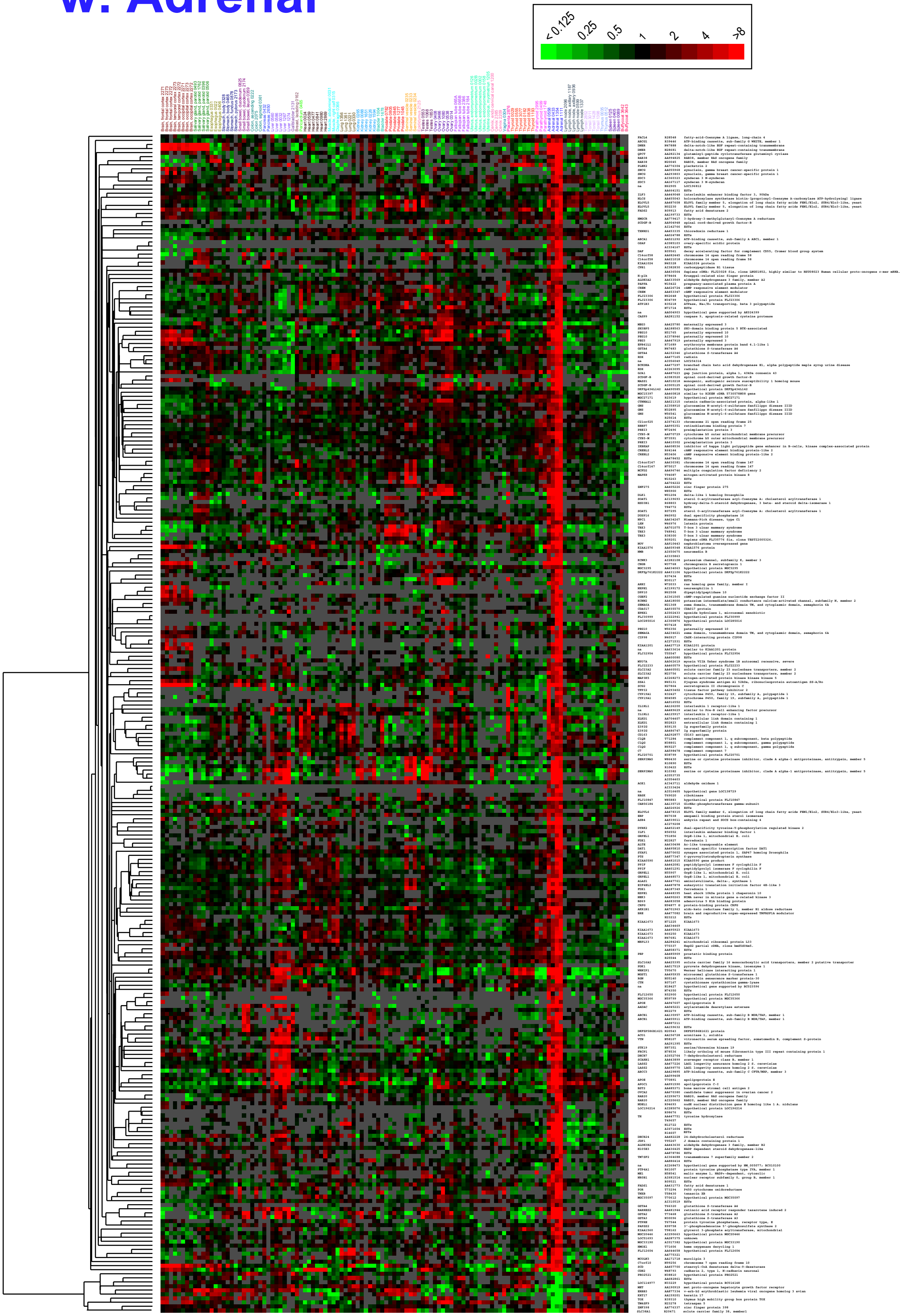

x. Lymph node

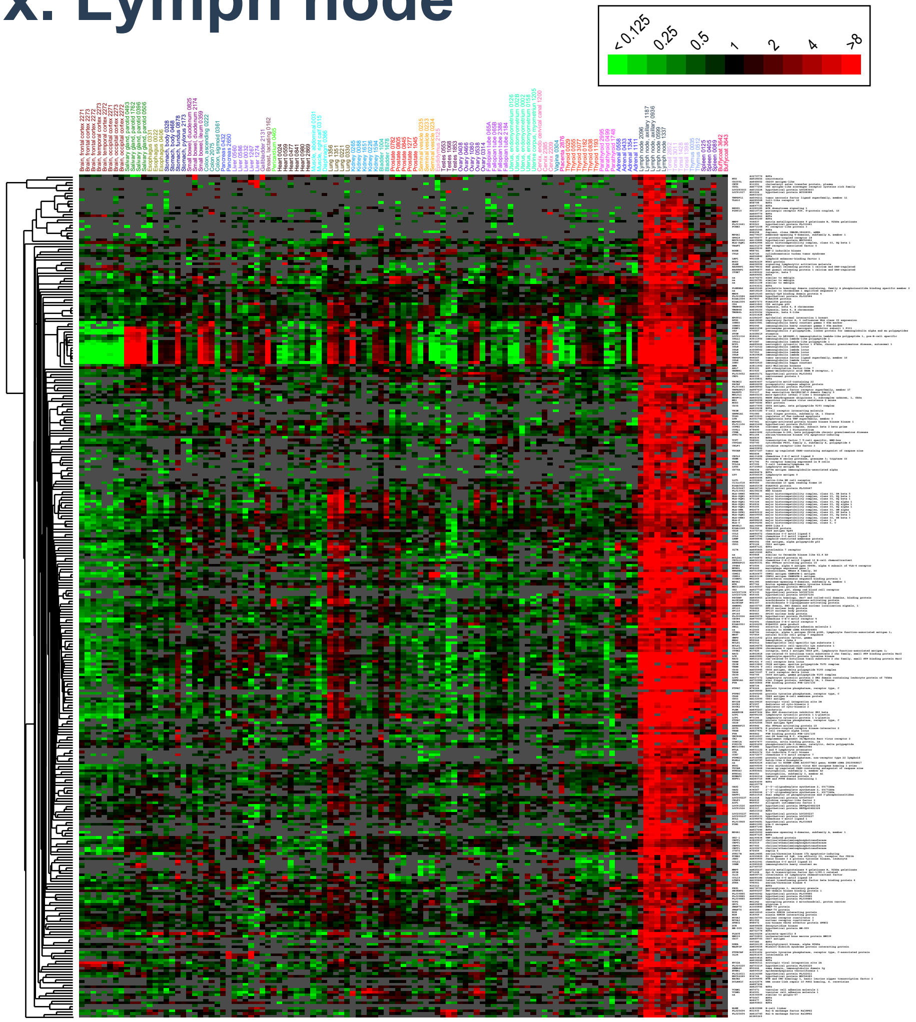

y. Tonsil

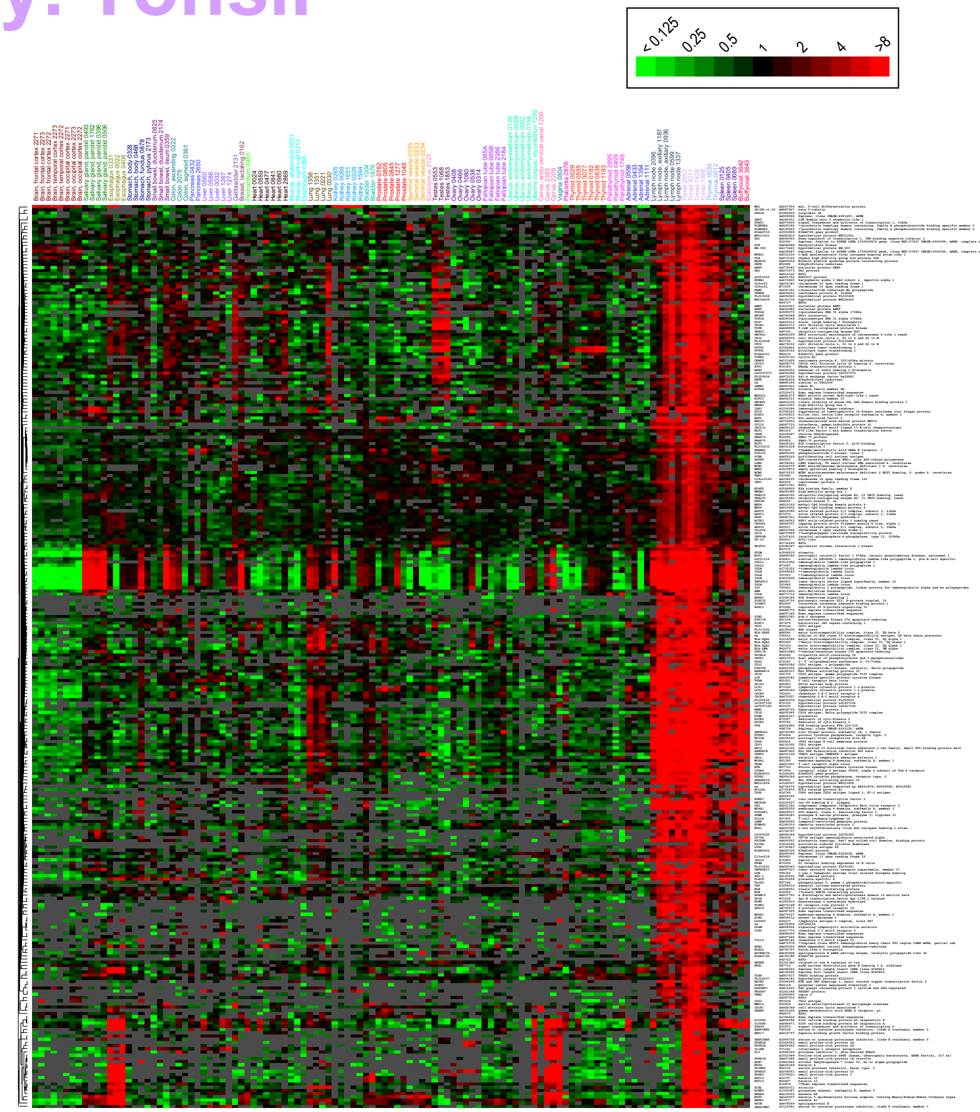

z. Thymus

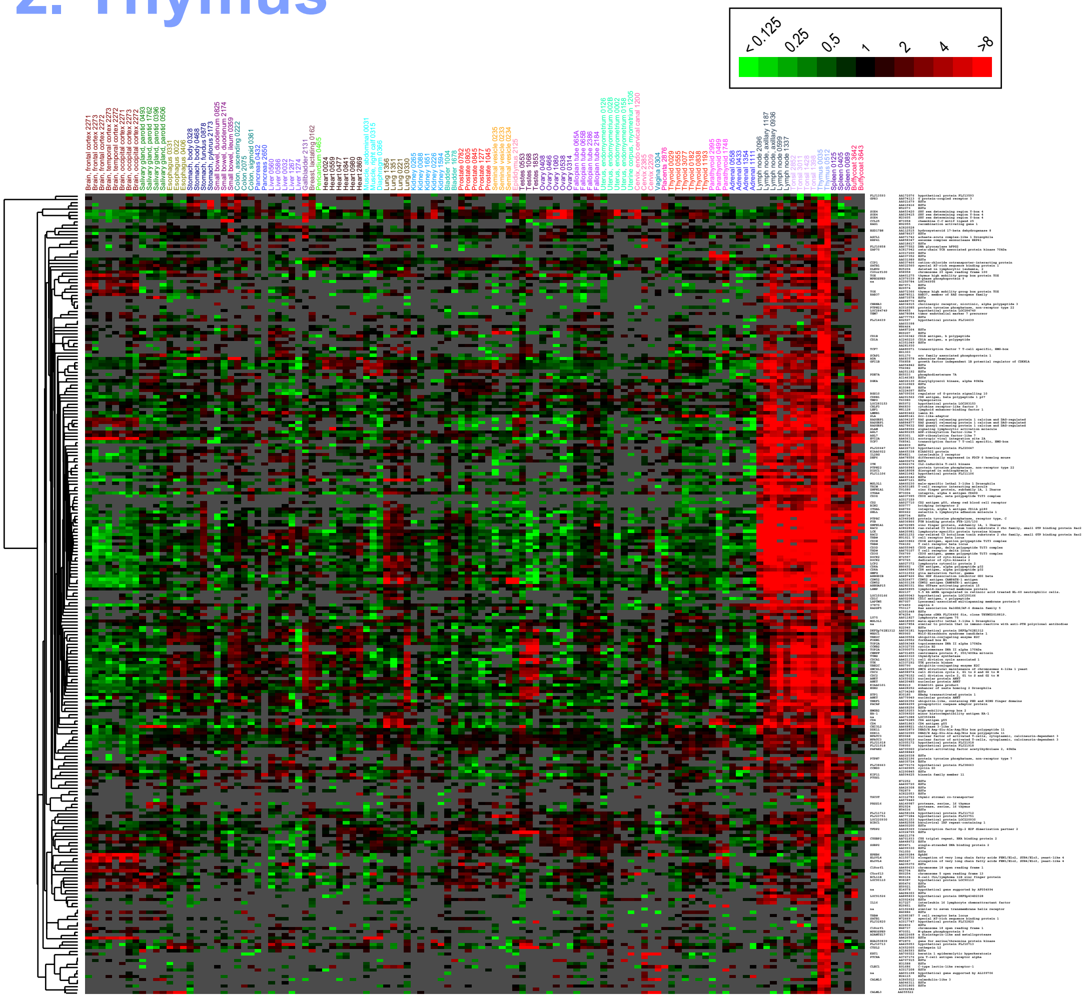

aa. Spleen

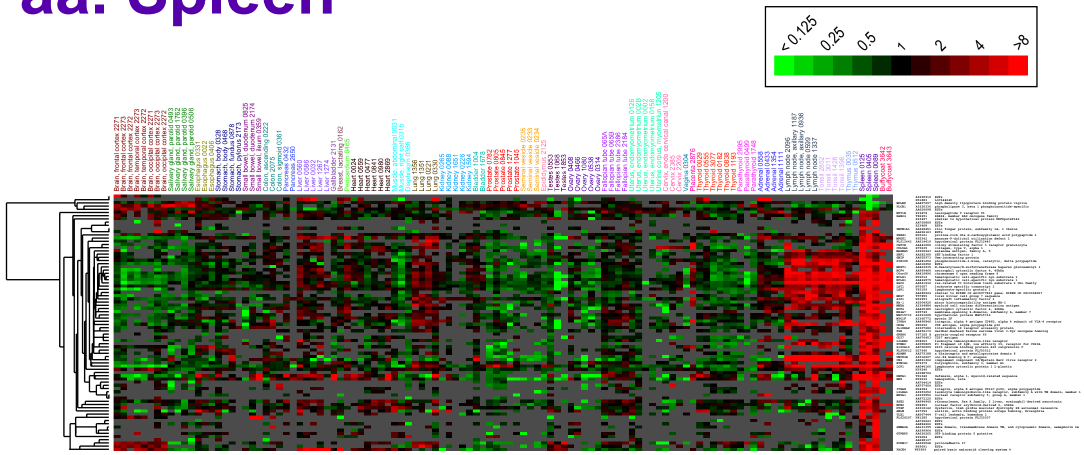

bb. Buffy coat

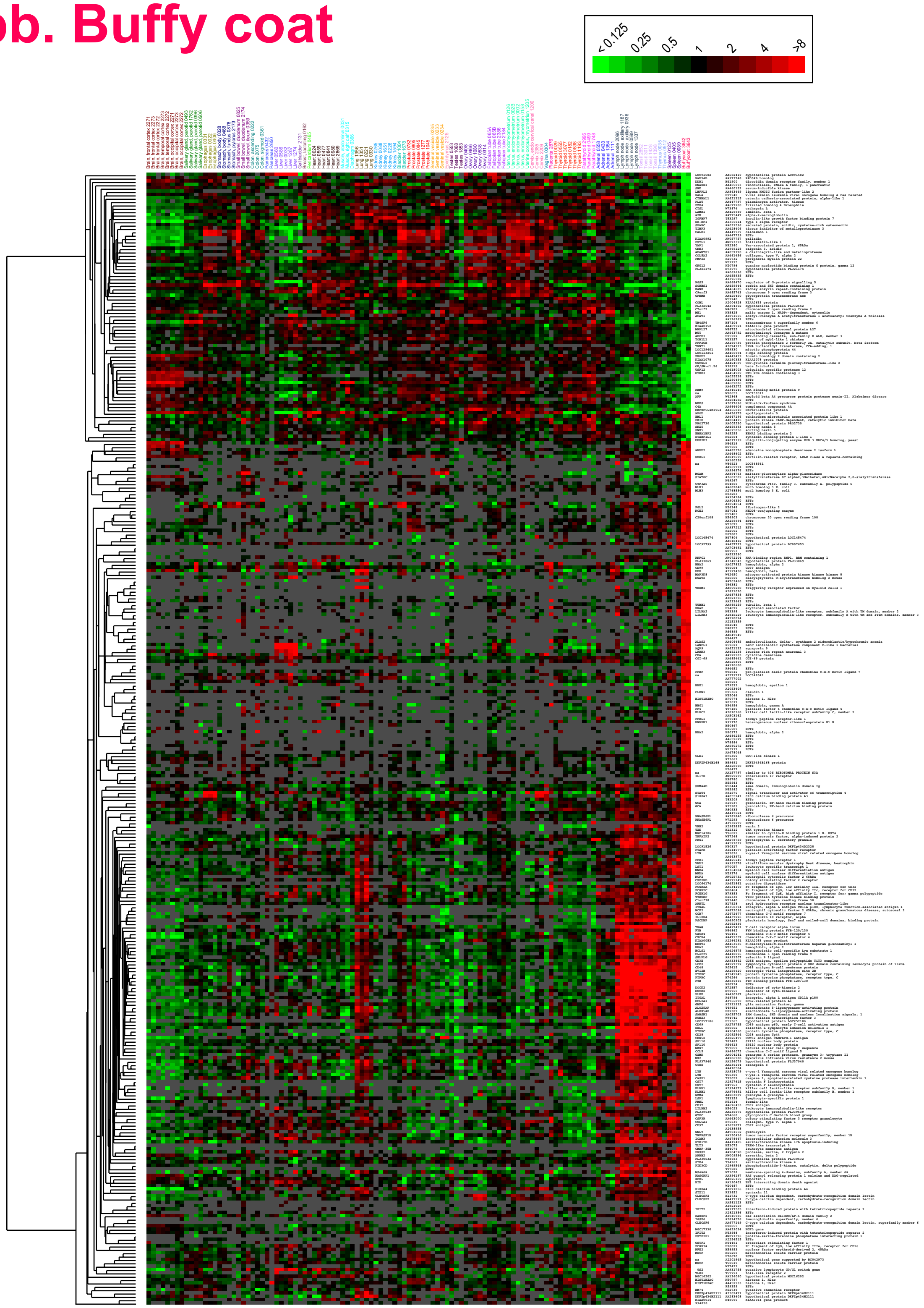

Supplement: Additional File 6 — A figure showing tissue-specific gene expression. Variably-expressed genes determined to be expressed in a tissue-selective fashion using the SAM method are depicted as described in the legend to manuscript Figure 2. a, brain; b, salivary gland; c, esophagus; d, stomach; e, small bowel; f, colon; g, pancreas; h, liver; i, heart; j, skeletal muscle; k, lung; l, kidney; m, bladder;n, prostate; o, seminal vesicle; p, testis; q, ovary; r, fallopian tube; s, uterus; t, cervix, u, thyroid; v, parathyroid; w, adrenal; x, lymph node; y, tonsil; z, thymus; aa, spleen; bb, buffy coat [file gb-2005-6-3-r22-S6.pdf]
