# Supplementary material for: A DNA microarray survey of gene expression in normal human tissues
Source: Genome Biol. 2005 Feb 14;6(3):R22. doi: 10.1186/gb-2005-6-3-r22 (PMC1088941; doi:10.1186/gb-2005-6-3-r22)

a. Tyrosine kinases

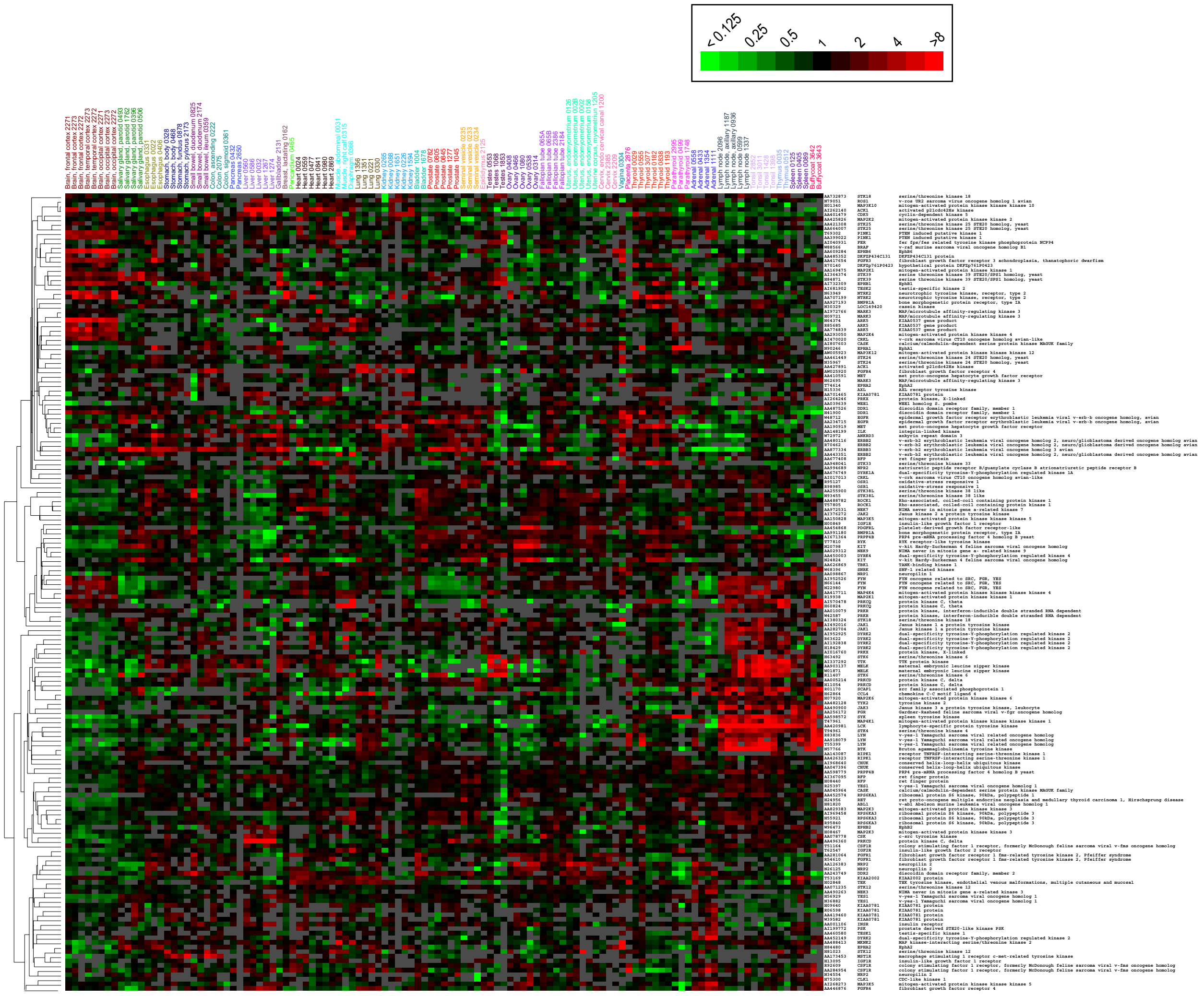

b. Kinases

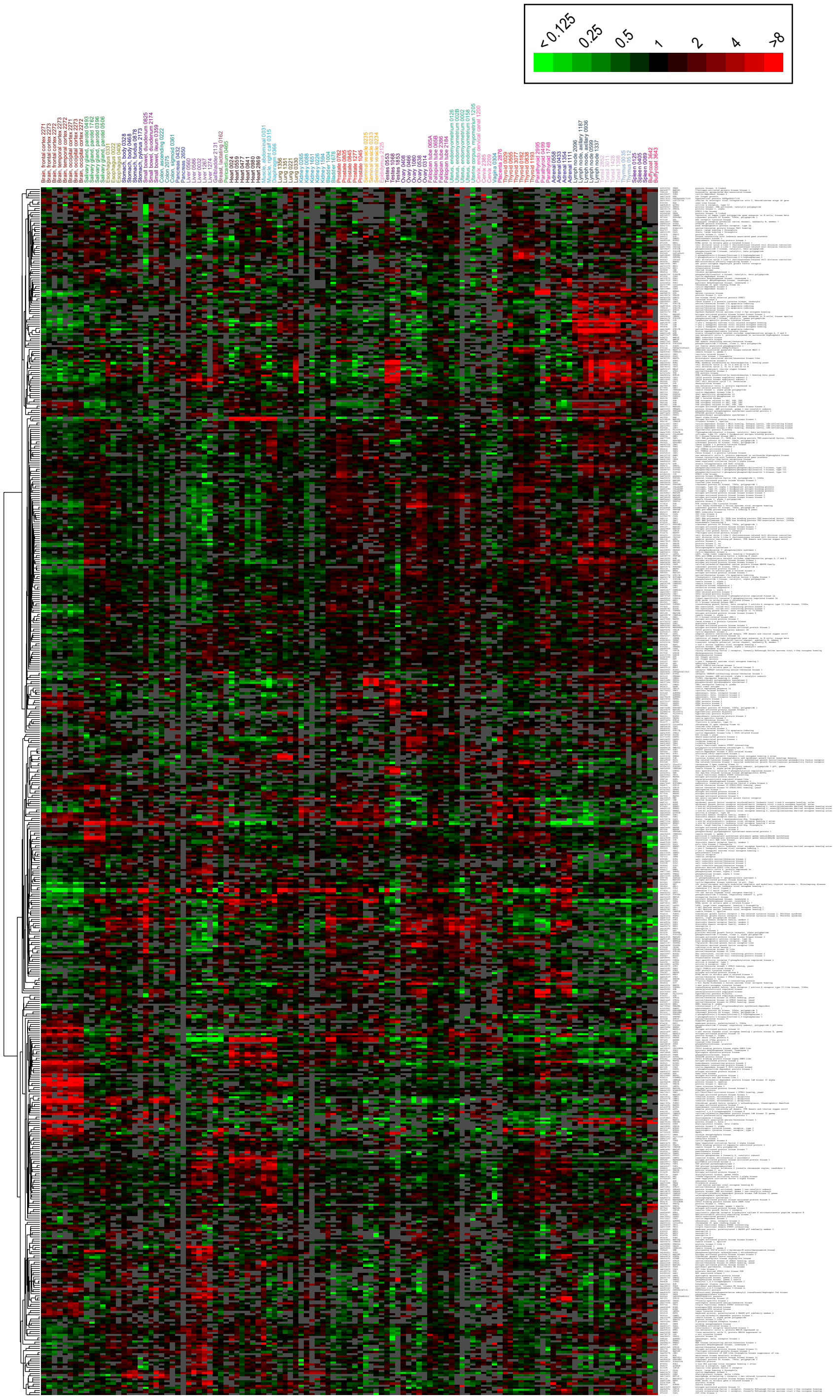

c. G-protein coupled receptors

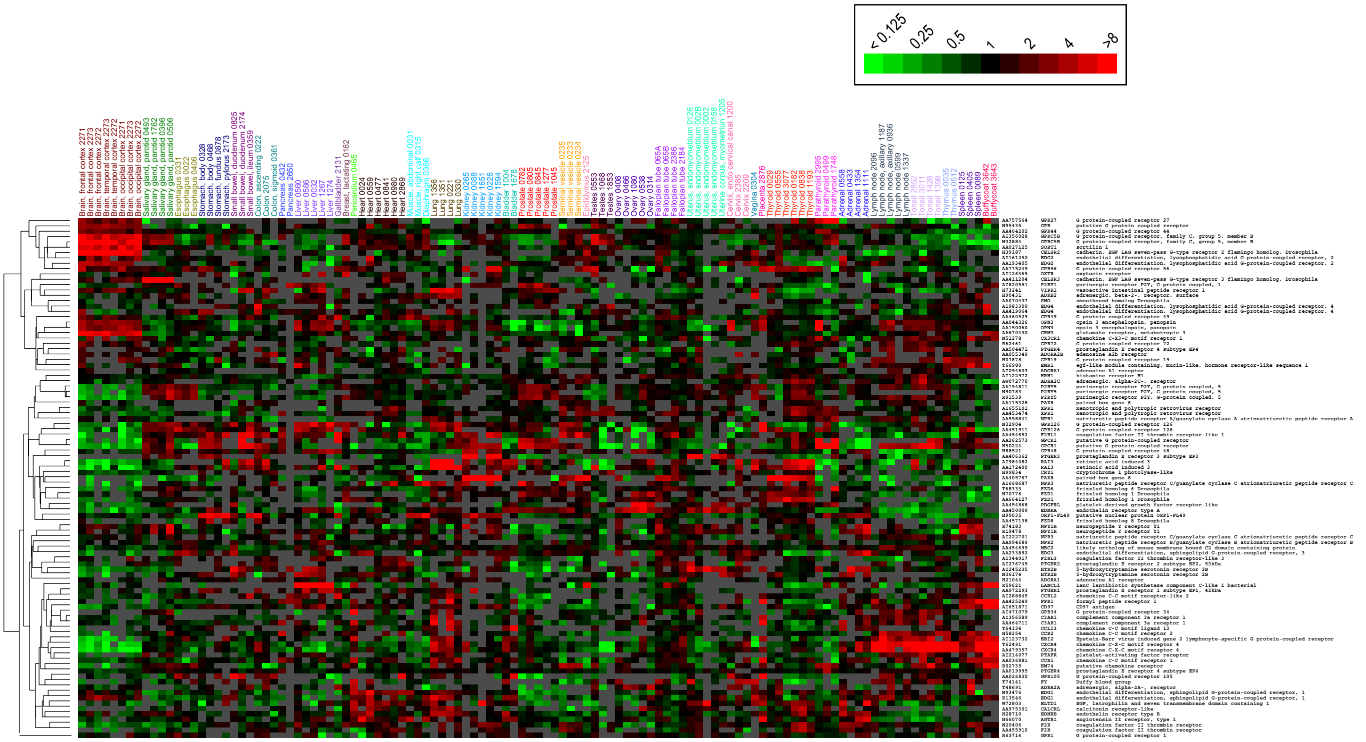

d. Transcription factors

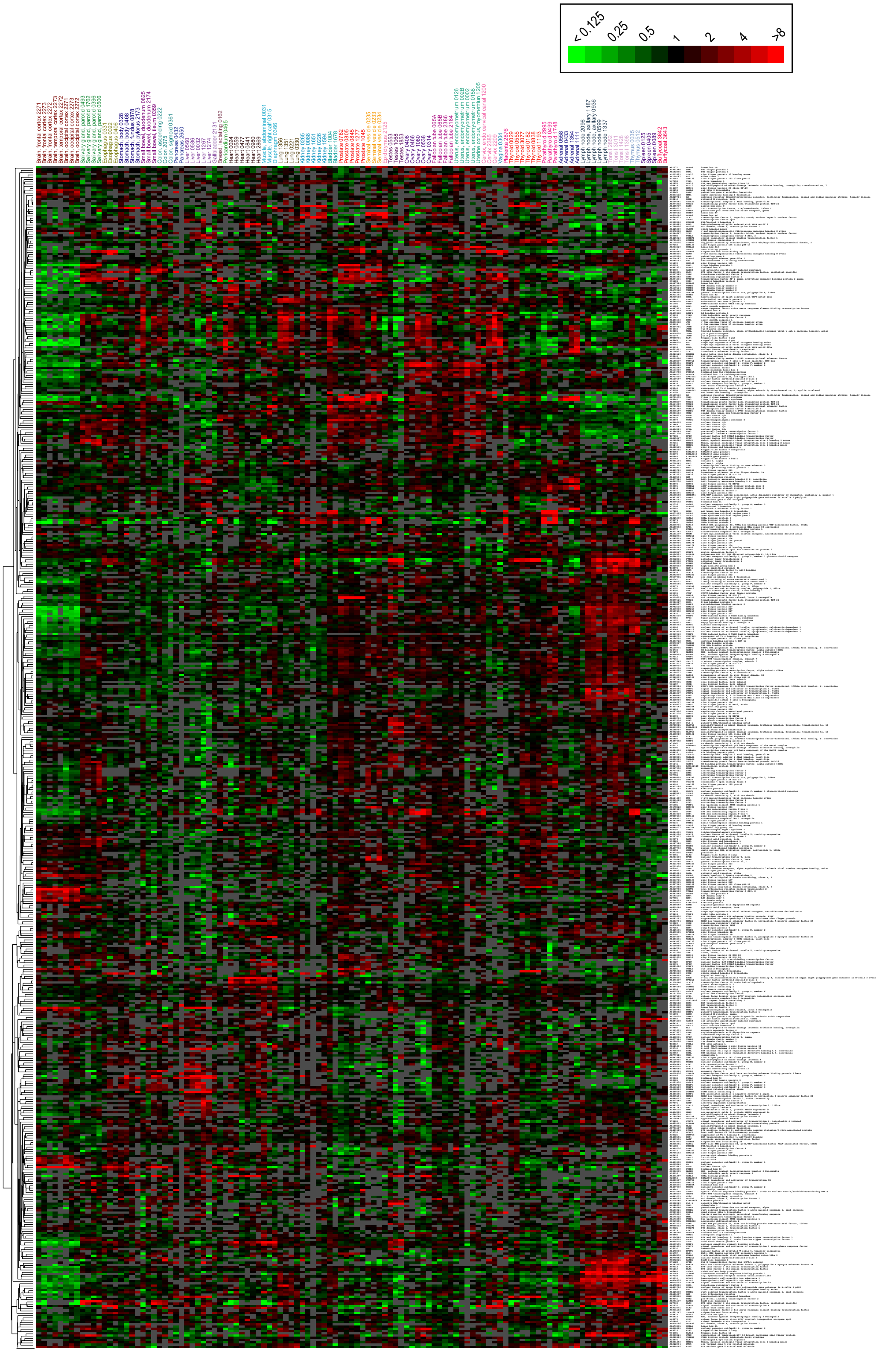

e. Ion channels

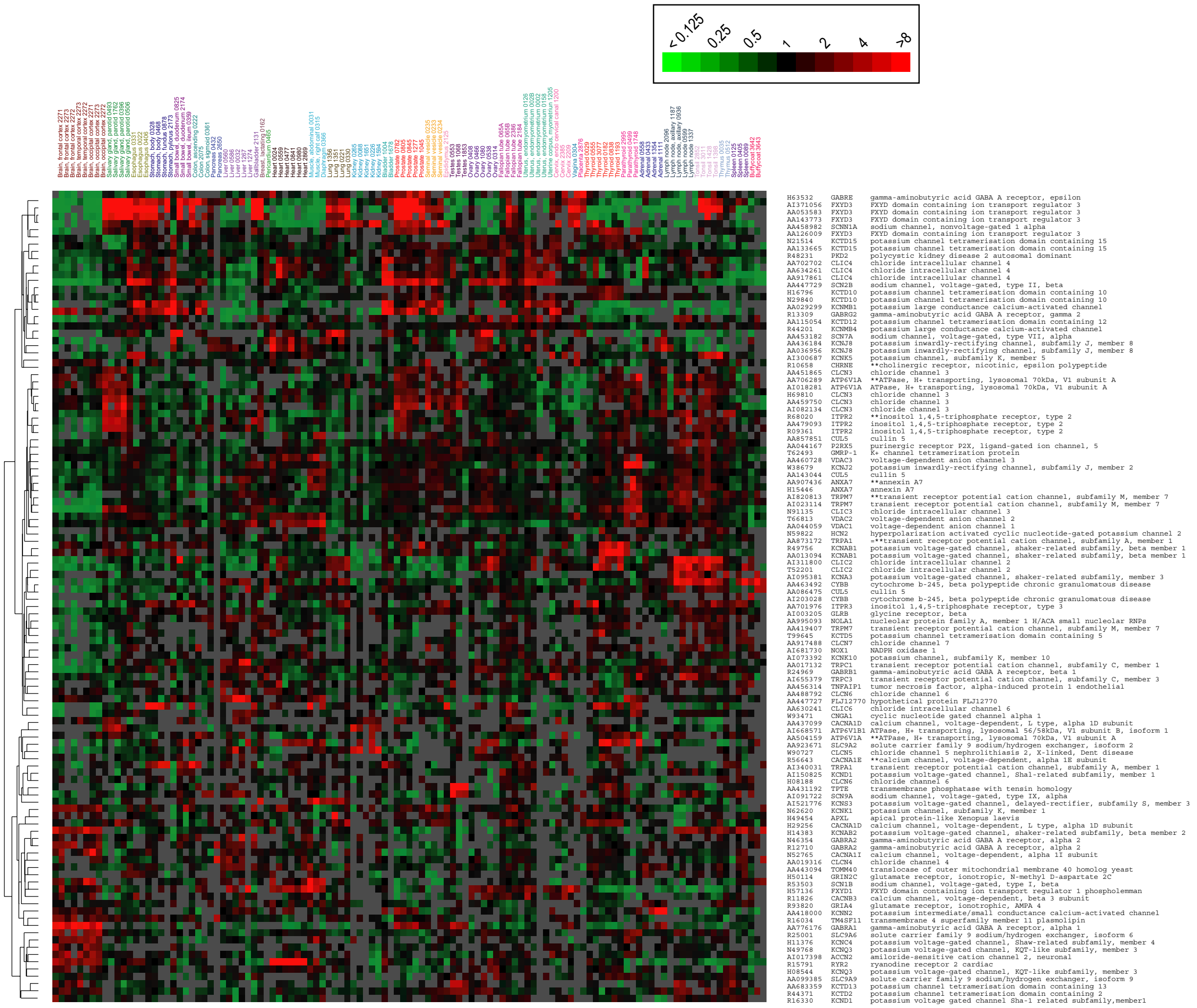

f. Extracellular matrix

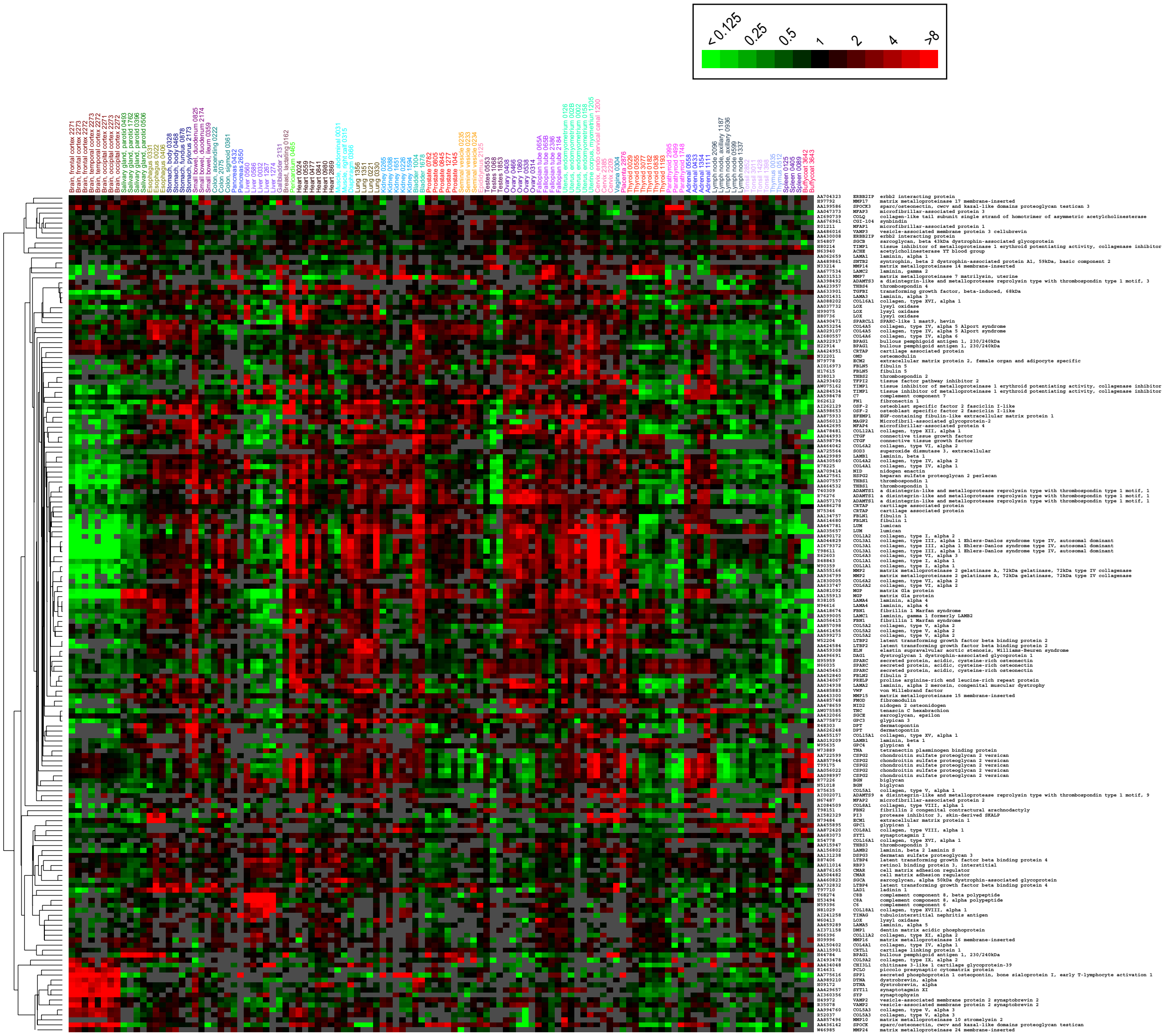

g. Cell adhesion

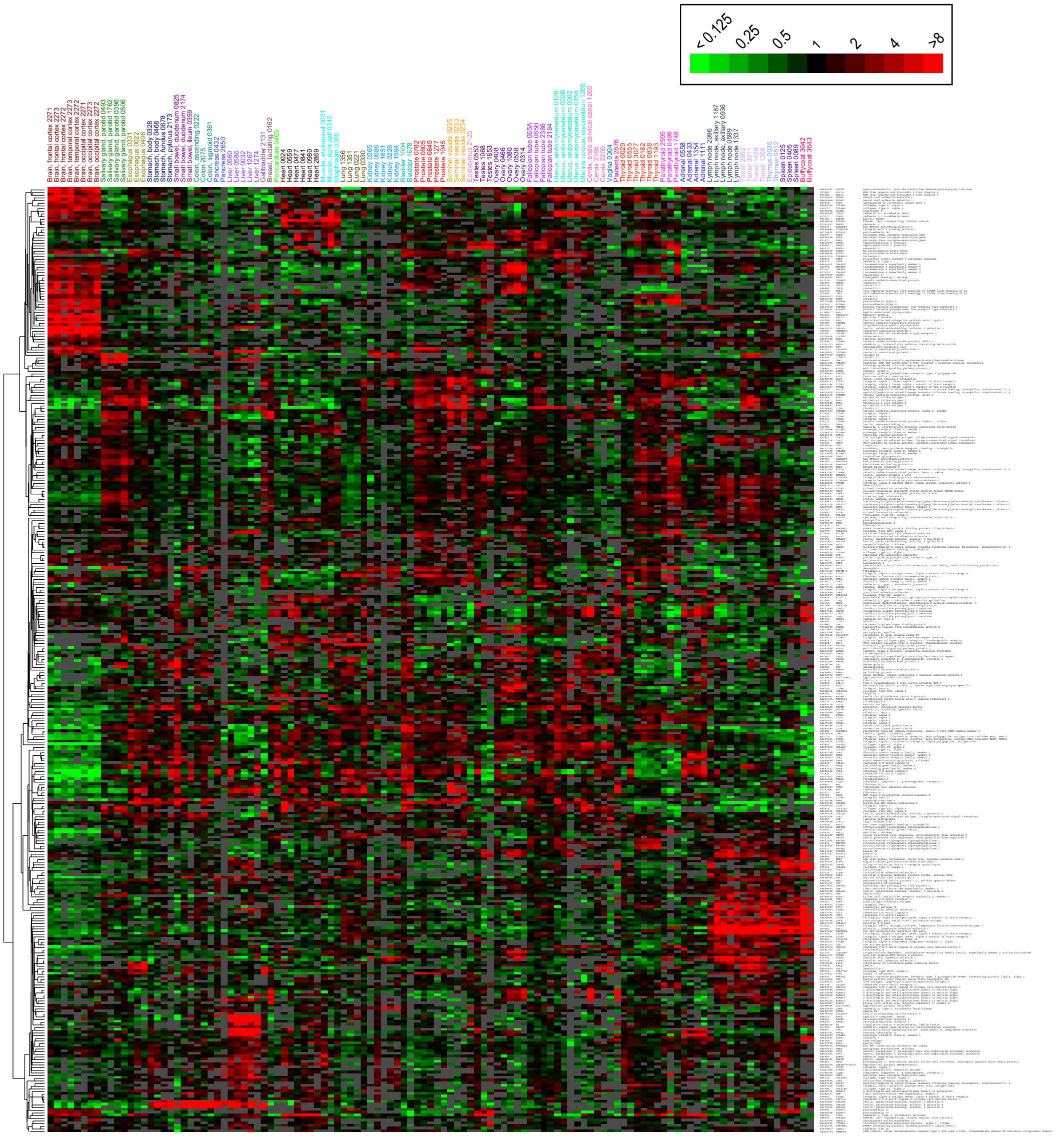

h. Programmed cell death

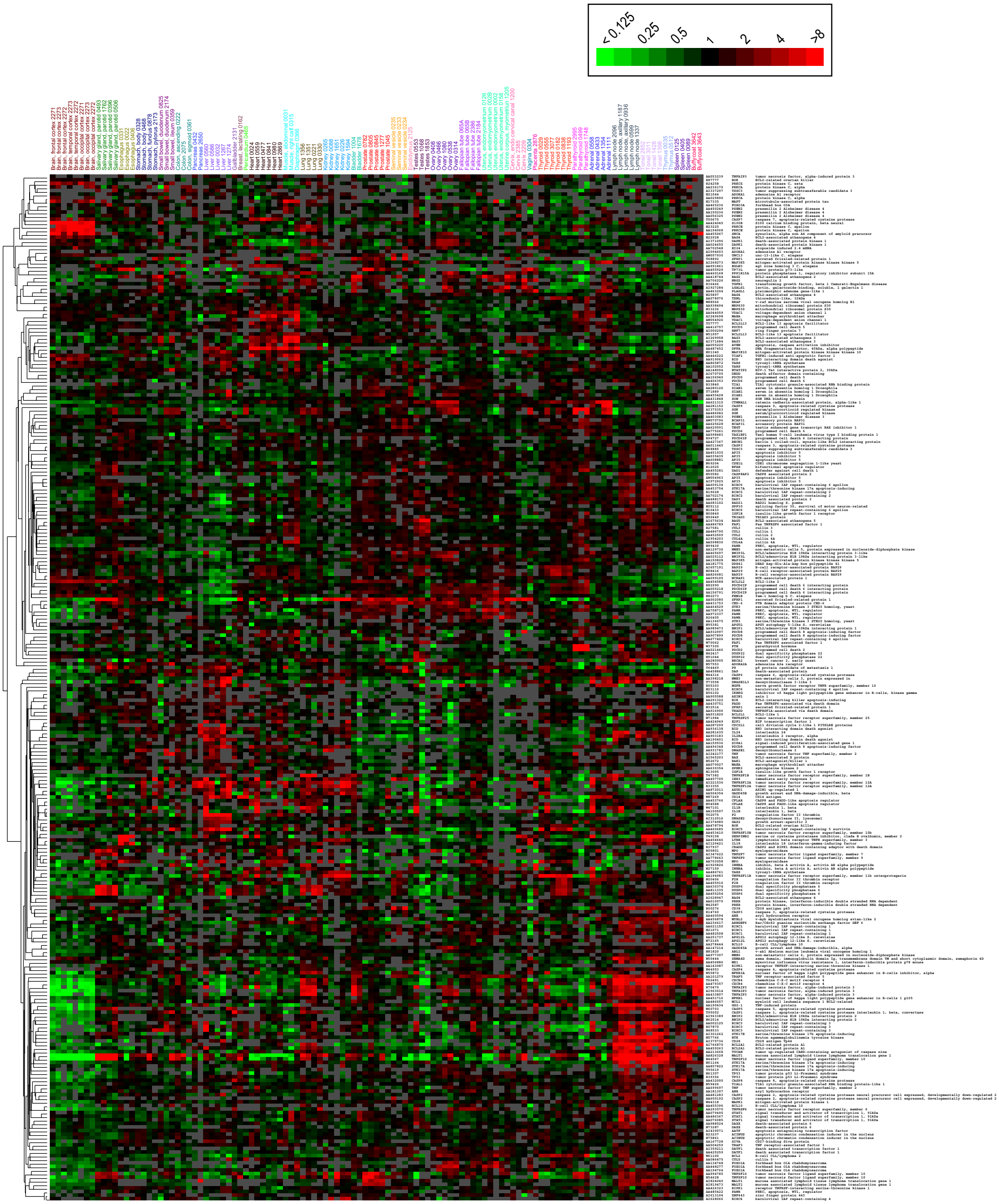

Supplement: Additional File 7 — A figure showing expression of functionally annotated gene sets. Hierarchical cluster of 115 normal tissue specimens and annotated gene sets representing examples of specific molecular functions, cellular components, or biological processes. a, tyrosine kinase (activity); b, kinase (activity); c, G-protein coupled receptor (activity); d, transcription factor activity; e, ion channel (activity); f, extracellular matrix (component), g, cell adhesion (process); h, programmed cell death (process) [file gb-2005-6-3-r22-S7.pdf]
