# Supplementary material for: A DNA microarray survey of gene expression in normal human tissues
Source: Genome Biol. 2005 Feb 14;6(3):R22. doi: 10.1186/gb-2005-6-3-r22 (PMC1088941; doi:10.1186/gb-2005-6-3-r22)

a. Brain

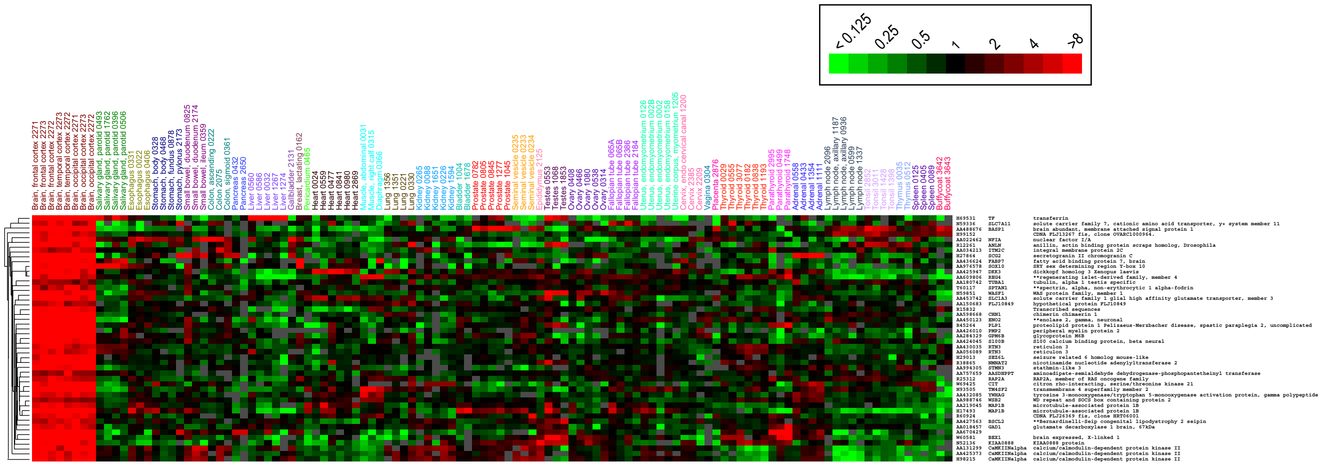

b. Salivary gland

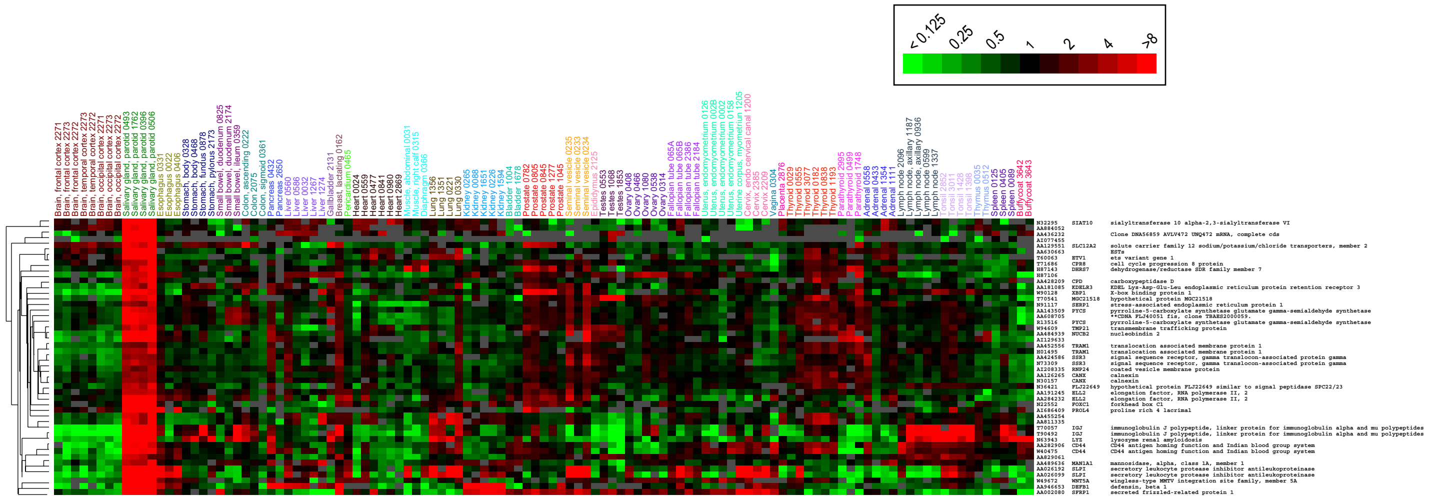

c. Esophagus

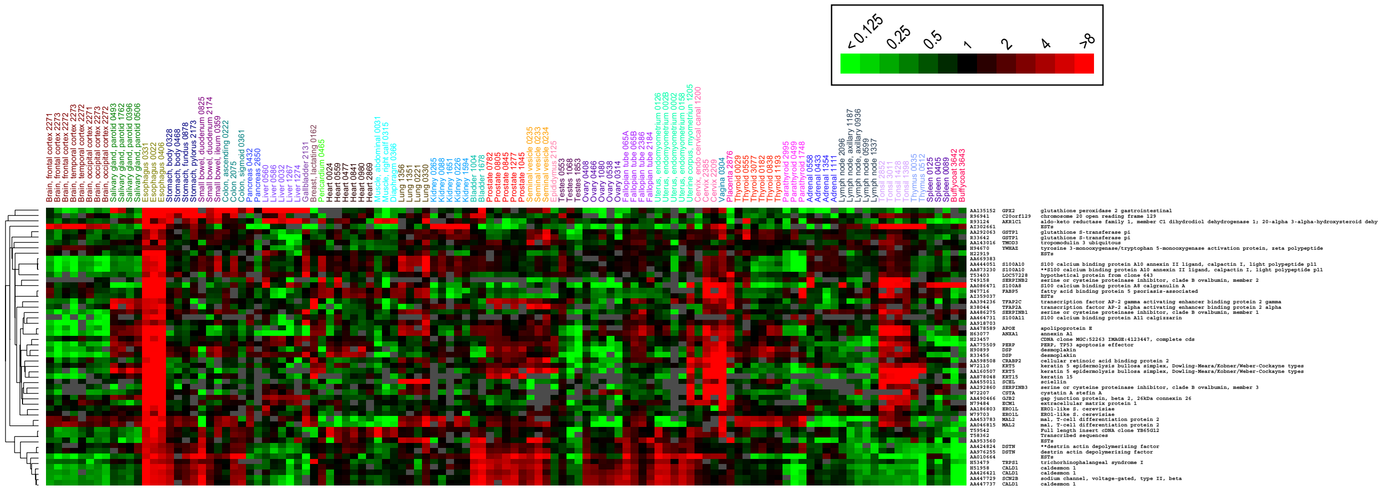

d. Stomach

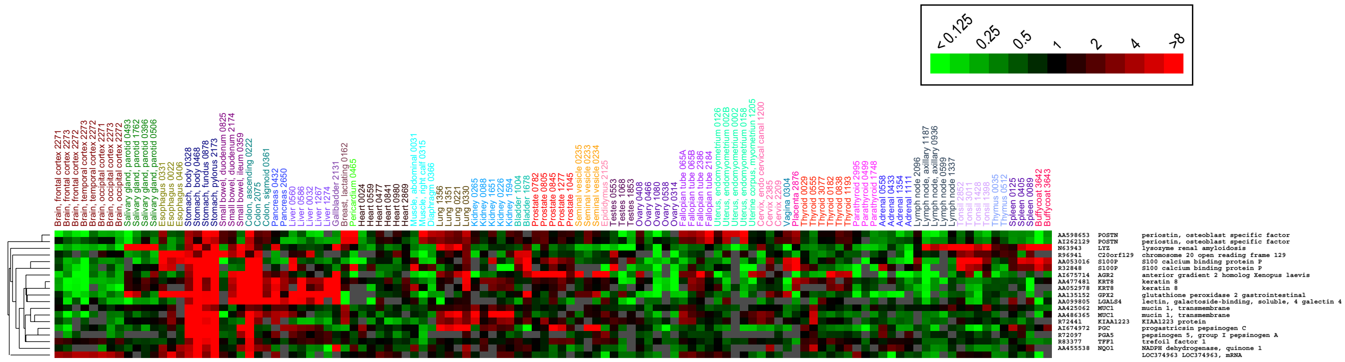

< 0.125
0.25
0.5
1
2
4
>8
AA598653 POSTN periostin, osteoblast specific factor
AI262129 POSTN periostin, osteoblast specific factor
N63943 LYZ lysozyme renal amyloidosis
R96941 C20orf129 chromosome 20 open reading frame 129
AA053016 S100P S100 calcium binding protein P
R32848 S100P S100 calcium binding protein P
AI675714 AGR2 anterior gradient 2 homolog Xenopus laevis
AA477481 KRT8 keratin 8
AA052978 KRT8 keratin 8
AA135152 GPX2 glutathione peroxidase 2 gastrointestinal
AA099805 LGALS4 lectin, galactoside-binding, soluble, 4 galectin 4
AA425062 MUC1 mucin 1, transmembrane
AA486365 MUC1 mucin 1, transmembrane
R72441 KIAA1223 KIAA1223 protein
AI674972 PGC progastricsin pepsinogen C
R72097 PGA5 pepsinogen 5, group I pepsinogen A
R83377 TFF1 trefoil factor 1
AA455538 NQO1 NADPH dehydrogenase, quinone 1
LOC374963 LOC374963, mRNA

e. Small bowel

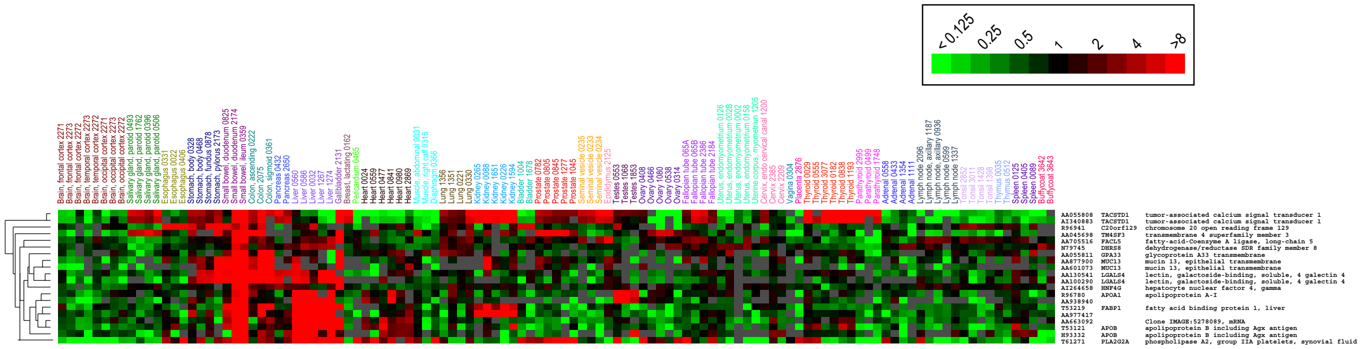

f. Colon

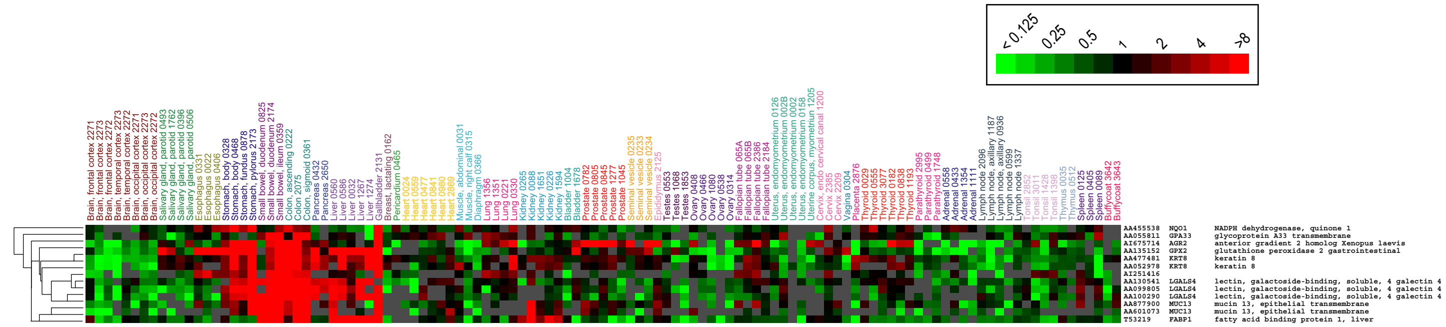

< 0.125
0.25
0.5
1
2
4
>8
AA455538 NQO1 NADPH dehydrogenase, quinone 1
AA055811 GPA33 glycoprotein A33 transmembrane
AI675714 AGR2 anterior gradient 2 homolog Xenopus laevis
AA135152 GPX2 glutathione peroxidase 2 gastrointestinal
AA477481 KRT8 keratin 8
AA052978 KRT8 keratin 8
AI251416
AA130541 LGALS4 lectin, galactoside-binding, soluble, 4 galectin 4
AA099805 LGALS4 lectin, galactoside-binding, soluble, 4 galectin 4
AA100290 LGALS4 lectin, galactoside-binding, soluble, 4 galectin 4
AA877900 MUC13 mucin 13, epithelial transmembrane
AA601073 MUC13 mucin 13, epithelial transmembrane
T53219 FABP1 fatty acid binding protein 1, liver

g. Pancreas

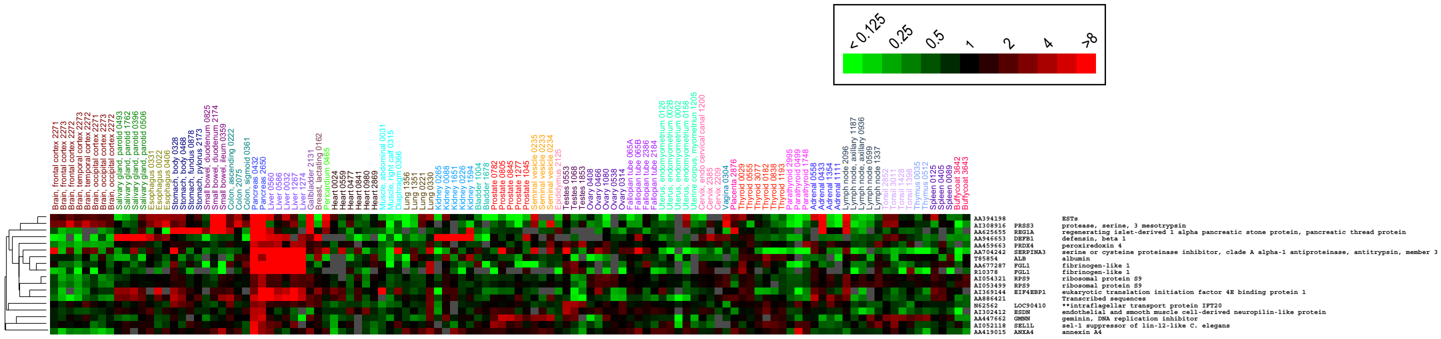

< 0.125
0.25
0.5
1
2
4
>8
AA394198 ESTs
AI308916 PRSS3 protease, serine, 3 mesotrypsin
AA625655 REG1A regenerating islet-derived 1 alpha pancreatic stone protein, pancreatic thread protein
AA946653 DEFB1 defensin, beta 1
AA459663 PRDX4 peroxiredoxin 4
AA704242 SERPINA3 serine or cysteine proteinase inhibitor, clade A alpha-1 antiproteinase, antitrypsin, member 3
T85854 ALB albumin
AA677287 FGL1 fibrinogen-like 1
R10378 FGL1 fibrinogen-like 1
AI054321 RPS9 ribosomal protein S9
AI053499 RPS9 ribosomal protein S9
AI369144 EIF4EBP1 eukaryotic translation initiation factor 4E binding protein 1
AA886421 Transcribed sequences
N62562 LOC90410 **intraflagellar transport protein IFT20
AI302412 ESDN endothelial and smooth muscle cell-derived neuropilin-like protein
AA447662 GMNN geminin, DNA replication inhibitor
AI052118 SEL1L sel-1 suppressor of lin-12-like C. elegans
AA419015 ANXA4 annexin A4
Spleen 0405
Spleen 0089
Buffycoat 3642
Buffycoat 3643

h. Liver

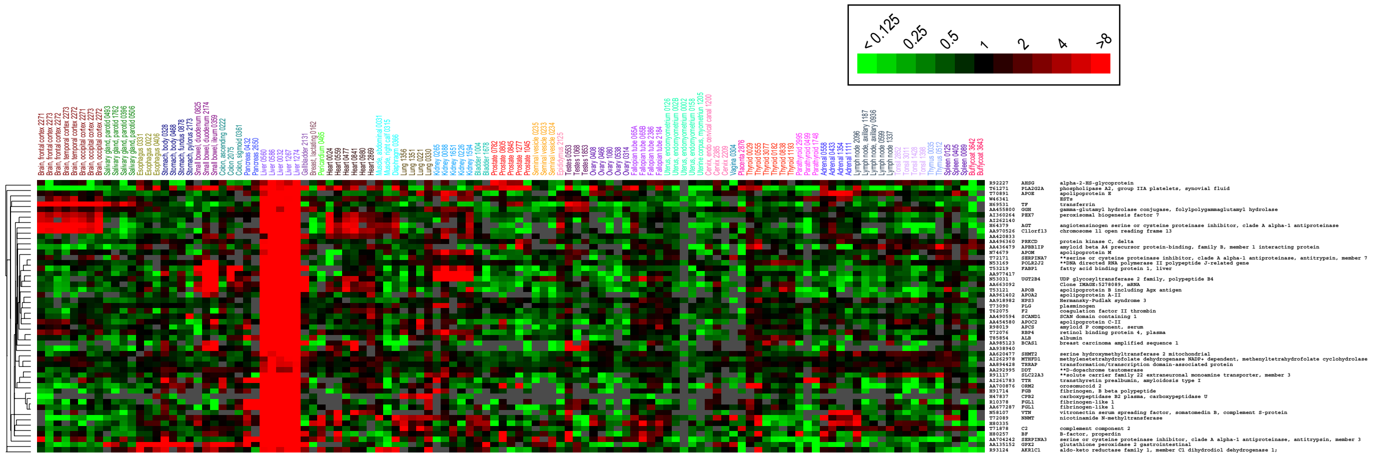

< 0.125
0.25
0.5
1
2
4
>8
R92227 AHSG alpha-2-HS-glycoprotein
T61271 PLA2G2A phospholipase A2, group IIA platelets, synovial fluid
T70891 APOE apolipoprotein E
W46341 ESTs
H69531 TF transferrin
AA455800 GGH gamma-glutamyl hydrolase conjugase, folylpolygammaglutamyl hydrolase
AI360264 PEX7 peroxisomal biogenesis factor 7
AI262140
H64379 AGT angiotensinogen serine or cysteine proteinase inhibitor, clade A alpha-1 antiproteinase
AA970526 C11orf13 chromosome 11 open reading frame 13
AA420833
AA496360 PRKCD protein kinase C, delta
AA436479 APBB1IP amyloid beta A4 precursor protein-binding, family B, member 1 interacting protein
N74679 APOM apolipoprotein M
T72171 SERPINA7 **serine or cysteine proteinase inhibitor, clade A alpha-1 antiproteinase, antitrypsin, member 7
N53169 POLR2J2 **DNA directed RNA polymerase II polypeptide J-related gene
T53219 FABP1 fatty acid binding protein 1, liver
AA977417
N53031 UGT2B4 UDP glycosyltransferase 2 family, polypeptide B4
AA663092 Clone IMAGE:5278089, mRNA
T53121 APOB apolipoprotein B including Agx antigen
AA961402 APOA2 apolipoprotein A-II
AA918982 HPS3 Hermansky-Pudlak syndrome 3
T73090 PLG plasminogen
T62075 F2 coagulation factor II thrombin
AA490594 SCAND1 SCAN domain containing 1
AA454580 APOC2 apolipoprotein C-II
R98019 APCS amyloid P component, serum
T72076 RBP4 retinol binding protein 4, plasma
T85854 ALB albumin
AA985123 BCAS1 breast carcinoma amplified sequence 1
AA938940
AA620477 SHMT2 serine hydroxymethyltransferase 2 mitochondrial
AI262978 MTHFD1 methylenetetrahydrofolate dehydrogenase NADP+ dependent, methenyltetrahydrofolate cyclohydrolase
AA894428 TRRAP transformation/transcription domain-associated protein
AA292995 DDT **D-dopachrome tautomerase
R91117 SLC22A3 **solute carrier family 22 extraneuronal monoamine transporter, member 3
AI261783 TTR transthyretin prealbumin, amyloidosis type I
AA700876 ORM2 orosomucoid 2
H91714 FGB fibrinogen, B beta polypeptide
H47837 CPB2 carboxypeptidase B2 plasma, carboxypeptidase U
R10378 FGL1 fibrinogen-like 1
AA677287 FGL1 fibrinogen-like 1
N58107 VTN vitronectin serum spreading factor, somatomedin B, complement S-protein
T72089 NNMT nicotinamide N-methyltransferase
H80335
T71878 C2 complement component 2
H80257 BF B-factor, properdin
AA704242 SERPINA3 serine or cysteine proteinase inhibitor, clade A alpha-1 antiproteinase, antitrypsin, member 3
AA135152 GPX2 glutathione peroxidase 2 gastrointestinal
R93124 AKR1C1 aldo-keto reductase family 1, member C1 dihydrodiol dehydrogenase 1;

i. Heart

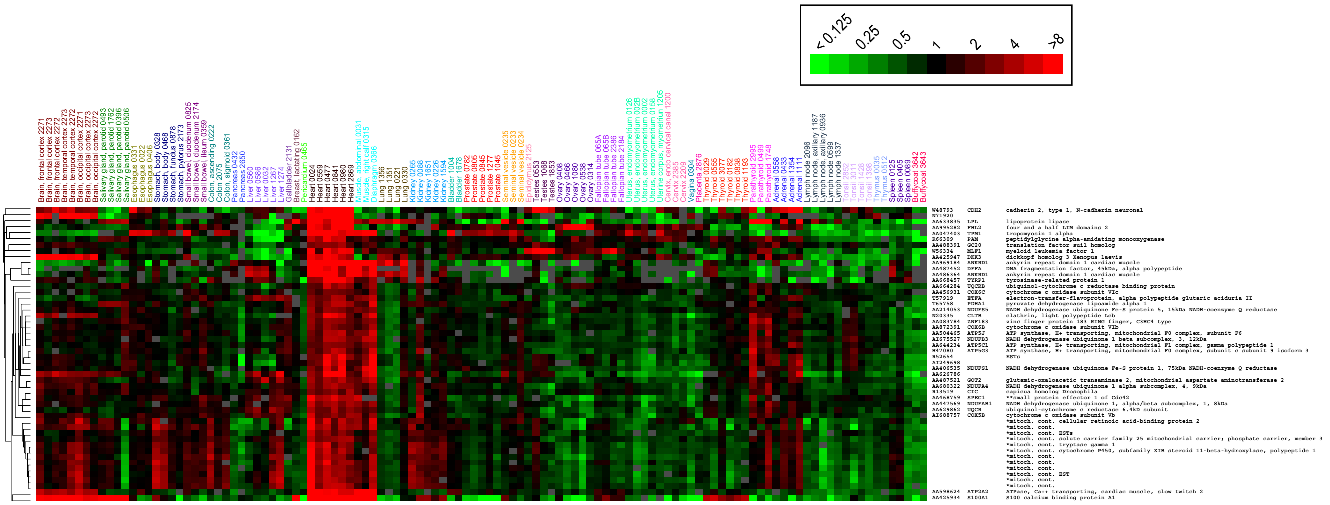

< 0.125
0.25
0.5
1
2
4
>8
W48793 CDH2 cadherin 2, type 1, N-cadherin neuronal
N71920
AA633835 LPL lipoprotein lipase
AA995282 FHL2 four and a half LIM domains 2
AA047403 TPM1 tropomyosin 1 alpha
R66309 PAM peptidylglycine alpha-amidating monooxygenase
AA488391 GC20 translation factor sui1 homolog
W56334 MLF1 myeloid leukemia factor 1
AA425947 DKK3 dickkopf homolog 3 Xenopus laevis
AA969184 ANKRD1 ankyrin repeat domain 1 cardiac muscle
AA487452 DFFA DNA fragmentation factor, 45kDa, alpha polypeptide
AA486364 ANKRD1 ankyrin repeat domain 1 cardiac muscle
AA668457 TYRP1 tyrosinase-related protein 1
AA664284 UQCRB ubiquinol-cytochrome c reductase binding protein
AA456931 COX6C cytochrome c oxidase subunit VIc
T57919 ETFA electron-transfer-flavoprotein, alpha polypeptide glutaric aciduria II
T65758 PDHA1 pyruvate dehydrogenase lipoamide alpha 1
AA214053 NDUFS5 NADH dehydrogenase ubiquinone Fe-S protein 5, 15kDa NADH-coenzyme Q reductase
N20335 CLTB clathrin, light polypeptide Lcb
AA083784 ZNF183 zinc finger protein 183 RING finger, C3HC4 type
AA872391 COX6B cytochrome c oxidase subunit VIb
AA504465 ATP5J ATP synthase, H+ transporting, mitochondrial F0 complex, subunit F6
AI675527 NDUFB3 NADH dehydrogenase ubiquinone 1 beta subcomplex, 3, 12kDa
AA644234 ATP5C1 ATP synthase, H+ transporting, mitochondrial F1 complex, gamma polypeptide 1
H47080 ATP5G3 ATP synthase, H+ transporting, mitochondrial F0 complex, subunit c subunit 9 isoform 3
R52654 ESTs
AI249698
AA406535 NDUFS1 NADH dehydrogenase ubiquinone Fe-S protein 1, 75kDa NADH-coenzyme Q reductase
AA626786
AA487521 GOT2 glutamic-oxaloacetic transaminase 2, mitochondrial aspartate aminotransferase 2
AA680322 NDUFA4 NADH dehydrogenase ubiquinone 1 alpha subcomplex, 4, 9kDa
R13519 CIC capicua homolog Drosophila
AA468759 SPEC1 **small protein effector 1 of Cdc42
AA447569 NDUFAB1 NADH dehydrogenase ubiquinone 1, alpha/beta subcomplex, 1, 8kDa
AA629862 UQCR ubiquinol-cytochrome c reductase 6.4kD subunit
AI688757 COX5B cytochrome c oxidase subunit Vb
*mitoch. cont. cellular retinoic acid-binding protein 2
*mitoch. cont.
*mitoch. cont. ESTs
*mitoch. cont. solute carrier family 25 mitochondrial carrier; phosphate carrier, member 3
*mitoch. cont. tryptase gamma 1
*mitoch. cont. cytochrome P450, subfamily XIB steroid 11-beta-hydroxylase, polypeptide 1
*mitoch. cont.
*mitoch. cont.
*mitoch. cont.
*mitoch. cont. EST
*mitoch. cont.
*mitoch. cont.
AA598624 ATP2A2 ATPase, Ca++ transporting, cardiac muscle, slow twitch 2
AA425934 S100A1 S100 calcium binding protein A1

j. Skeletal muscle

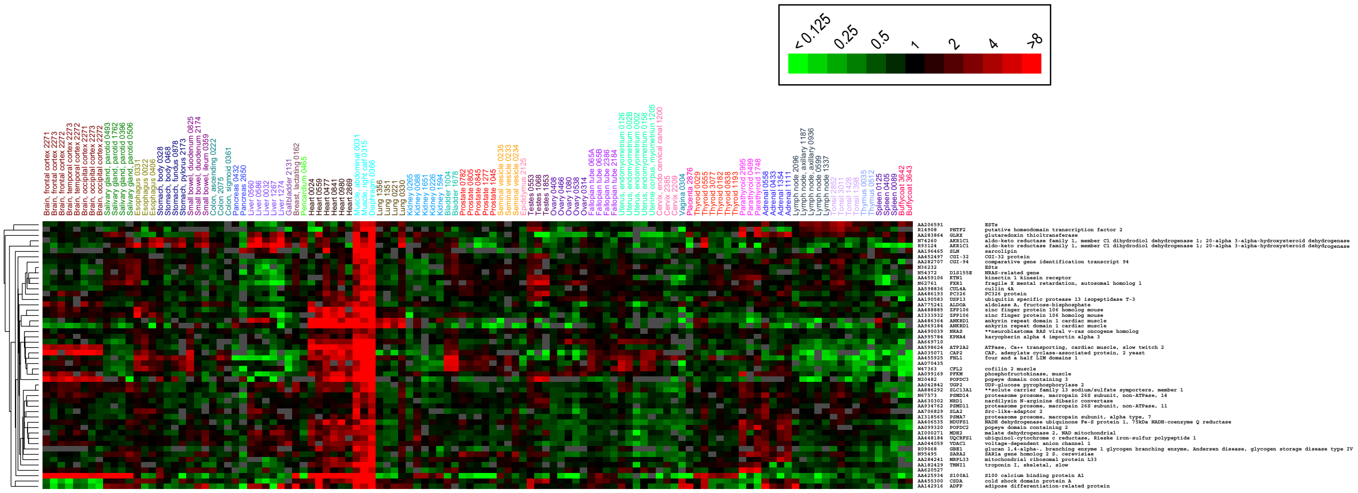

k. Lung

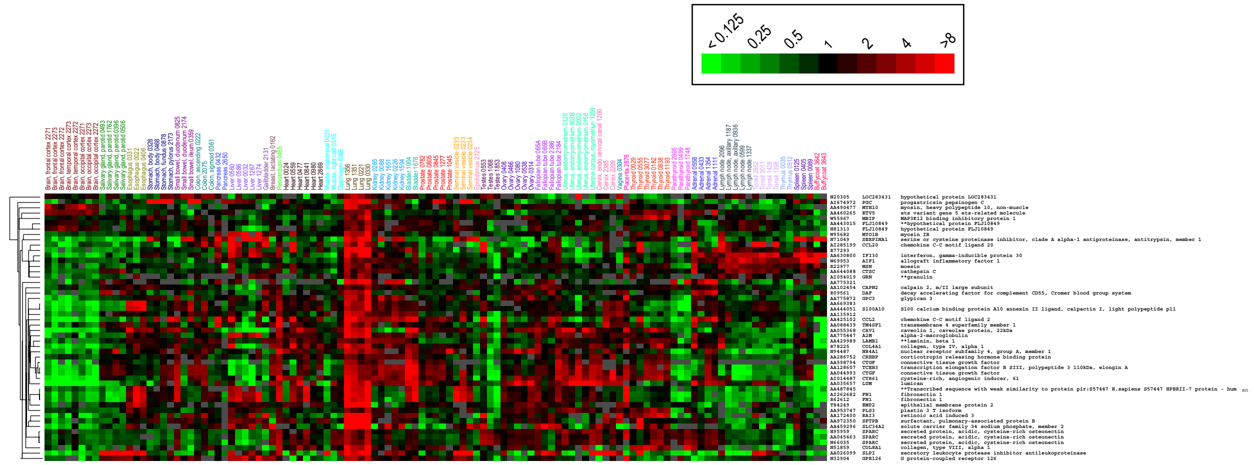

< 0.125
0.25
0.5
1
2
4
>8
N20305 LOC283431 hypothetical protein LOC283431
AI674972 PGC progastricsin pepsinogen C
AA490477 MYH10 myosin, heavy polypeptide 10, non-muscle
AA460265 ETV5 ets variant gene 5 ets-related molecule
W55967 MBIP MAP3K12 binding inhibitory protein 1
AA443015 FLJ10849 **hypothetical protein FLJ10849
H81313 FLJ10849 hypothetical protein FLJ10849
W95682 MYO1B myosin IB
N71049 SERPINA1 serine or cysteine proteinase inhibitor, clade A alpha-1 antiproteinase, antitrypsin, member 1
AI285199 CCL20 chemokine C-C motif ligand 20
R77293
AA630800 IFI30 interferon, gamma-inducible protein 30
W69953 AIF1 allograft inflammatory factor 1
R22977 MSN moesin
AA644088 CTSC cathepsin C
AI054019 GRN **granulin
AA775321
AA102454 CAPN2 calpain 2, m/II large subunit
R09561 DAF decay accelerating factor for complement CD55, Cromer blood group system
AA775872 GPC3 glypican 3
AA669383
AA444051 S100A10 S100 calcium binding protein A10 annexin II ligand, calpactin I, light polypeptide p11
AA135912
AA425102 CCL2 chemokine C-C motif ligand 2
AA088439 TM4SF1 transmembrane 4 superfamily member 1
AA055368 CAV1 caveolin 1, caveolae protein, 22kDa
AA775447 A2M alpha-2-macroglobulin
AA429989 LAMB1 **laminin, beta 1
R78225 COL4A1 collagen, type IV, alpha 1
N94487 NR4A1 nuclear receptor subfamily 4, group A, member 1
AA286752 CRHBP corticotropin releasing hormone binding protein
AA598794 CTGF connective tissue growth factor
AA128607 TCEB3 transcription elongation factor B SIII, polypeptide 3 110kDa, elongin A
AA044993 CTGF connective tissue growth factor
AI014487 CYR61 cysteine-rich, angiogenic inducer, 61
AA035657 LUM lumican
AA487845 **Transcribed sequence with weak similarity to protein pir:S57447 H.sapiens S57447 HPBRII-7 protein - hum an
AI262682 FN1 fibronectin 1
R62612 FN1 fibronectin 1
T84249 EMP2 epithelial membrane protein 2
AA953747 PLS3 plastin 3 T isoform
AA172400 RAI3 retinoic acid induced 3
AA972350 SFTPB surfactant, pulmonary-associated protein B
AA459296 SLC34A2 solute carrier family 34 sodium phosphate, member 2
H95959 SPARC secreted protein, acidic, cysteine-rich osteonectin
AA045463 SPARC secreted protein, acidic, cysteine-rich osteonectin
N66035 SPARC secreted protein, acidic, cysteine-rich osteonectin
N51859 COL8A1 collagen, type VIII, alpha 1
AA026099 SLPI secretory leukocyte protease inhibitor antileukoproteinase
N32904 GPR126 G protein-coupled receptor 126

I. Kidney

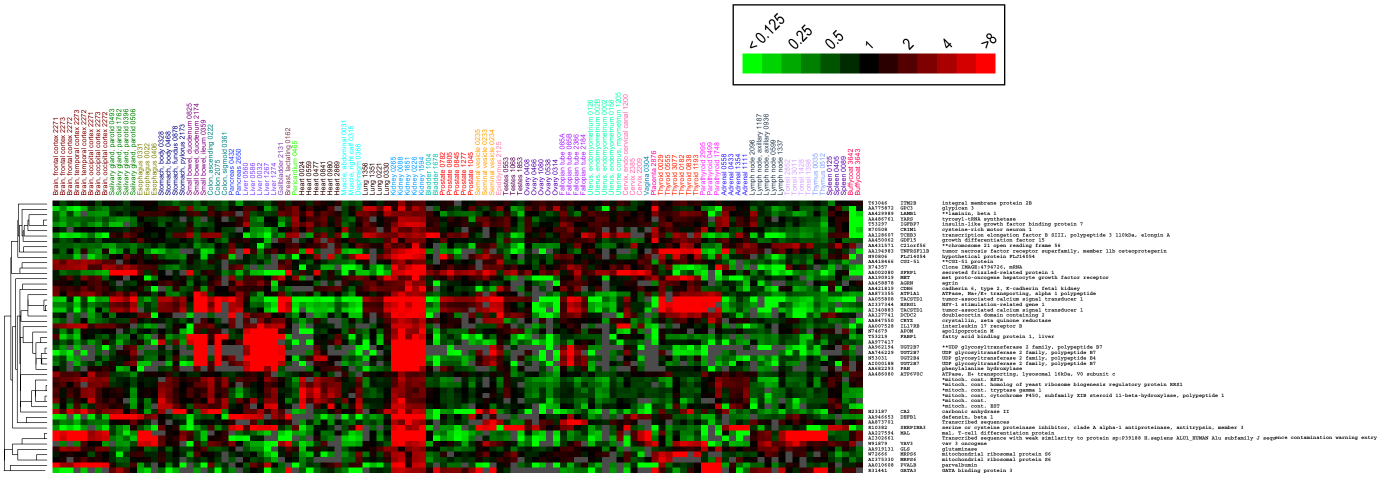

m. Bladder

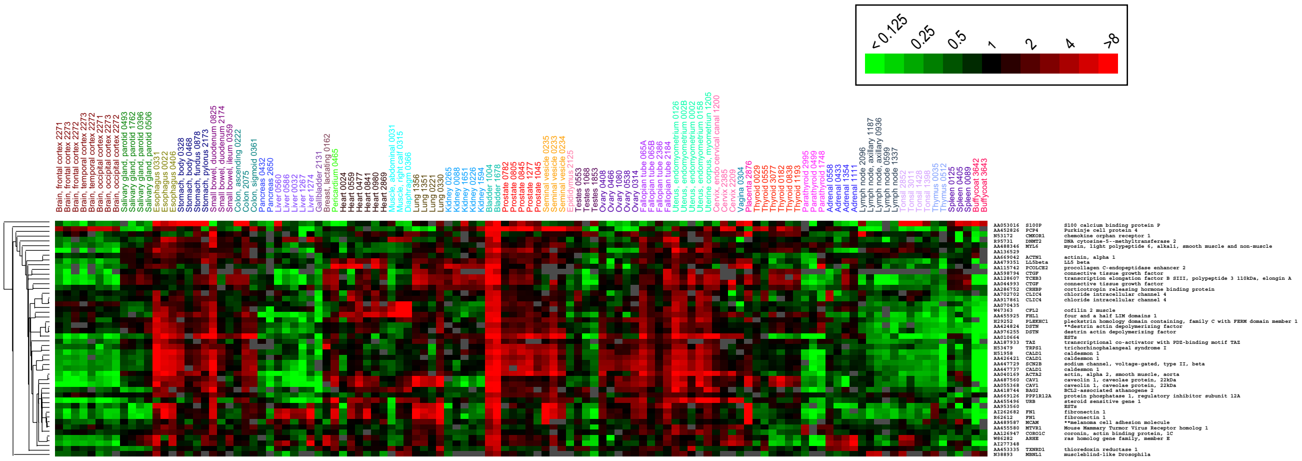

n. Prostate

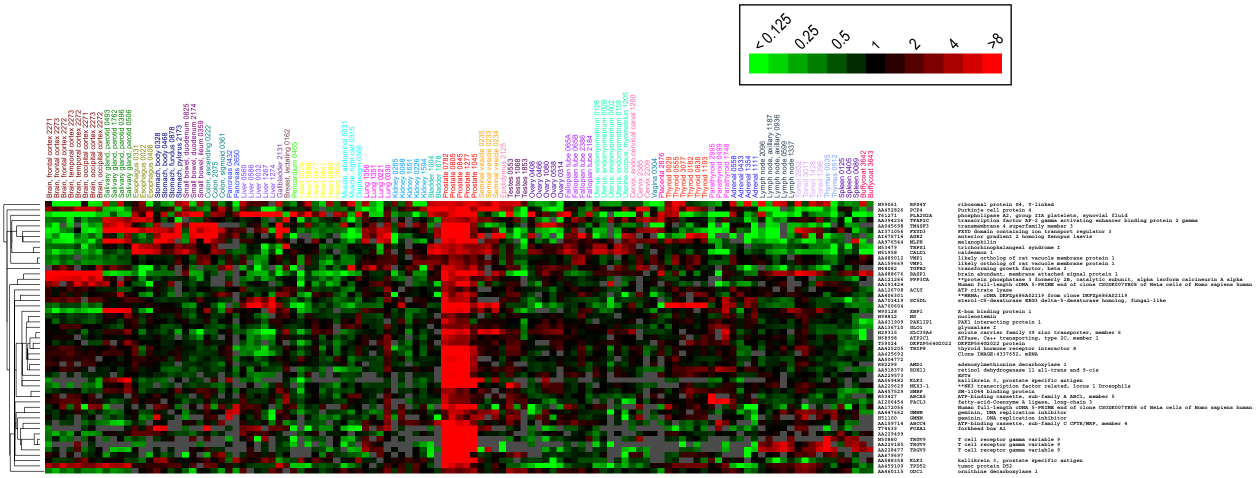

< 0.125
0.25
0.5
1
2
4
>8
N99061 RPS4Y ribosomal protein S4, Y-linked
AA452826 PCP4 Purkinje cell protein 4
T61271 PLA2G2A phospholipase A2, group IIA platelets, synovial fluid
AA394236 TFAP2C transcription factor AP-2 gamma activating enhancer binding protein 2 gamma
AA045698 TM4SF3 transmembrane 4 superfamily member 3
AI371056 FXYD3 FXYD domain containing ion transport regulator 3
AI675714 AGR2 anterior gradient 2 homolog Xenopus laevis
AA976544 MLPH melanophilin
H53479 TRPS1 trichorhinophalangeal syndrome I
H51958 CALD1 caldesmon 1
AA489012 VMP1 likely ortholog of rat vacuole membrane protein 1
AA159669 VMP1 likely ortholog of rat vacuole membrane protein 1
N48082 TGFB2 transforming growth factor, beta 2
AA488676 BASP1 brain abundant, membrane attached signal protein 1
AA121266 PPP3CA **protein phosphatase 3 formerly 2B, catalytic subunit, alpha isoform calcineurin A alpha
AA191424 Human full-length cDNA 5-PRIME end of clone CS0DK007YB08 of HeLa cells of Homo sapiens human
AA126708 ACLY ATP citrate lyase
AA406301 **MRNA; cDNA DKFZp686A02119 from clone DKFZp686A02119
AA703419 SC5DL sterol-C5-desaturase ERG3 delta-5-desaturase homolog, fungal-like
AA700604
W90128 XBP1 X-box binding protein 1
H98812 NS nucleostemin
AA431909 PAK1IP1 PAK1 interacting protein 1
AA136710 GLO1 glyoxalase I
H29315 SLC39A6 solute carrier family 39 zinc transporter, member 6
N68998 ATP2C1 ATPase, Ca++ transporting, type 2C, member 1
T59024 DKFZP564G2022 DKFZP564G2022 protein
AA425205 TRIP8 thyroid hormone receptor interactor 8
AA425692 Clone IMAGE:4337652, mRNA
AA504772
R82299 AMD1 adenosylmethionine decarboxylase 1
AA918370 RDH11 retinol dehydrogenase 11 all-trans and 9-cis
AA229573 ESTs
AA569482 KLK3 kallikrein 3, prostate specific antigen
AA229629 NKX3-1 **NK3 transcription factor related, locus 1 Drosophila
AA457529 SMBP SM-11044 binding protein
R53427 ABCA5 ATP-binding cassette, sub-family A ABC1, member 5
AI206454 FACL3 fatty-acid-Coenzyme A ligase, long-chain 3
AA172056 Human full-length cDNA 5-PRIME end of clone CS0DK007YB08 of HeLa cells of Homo sapiens human
AA447662 GMNN geminin, DNA replication inhibitor
H51100 GMNN geminin, DNA replication inhibitor
AA159714 ABCC4 ATP-binding cassette, sub-family C CFTR/MRP, member 4
T74639 FOXA1 forkhead box A1
AA229499
N50880 TRGV9 T cell receptor gamma variable 9
AA229185 TRGV9 T cell receptor gamma variable 9
AA228477 TRGV9 T cell receptor gamma variable 9
AA679697
AA588358 KLK3 kallikrein 3, prostate specific antigen
AA459100 TPD52 tumor protein D52
AA460115 ODC1 ornithine decarboxylase 1

o. Seminal vesicle

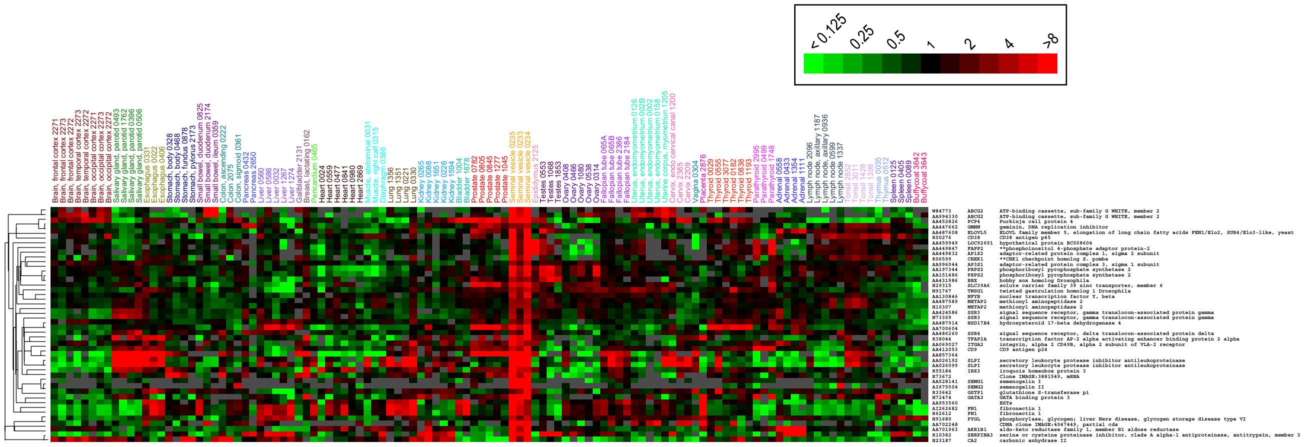

p. Testis

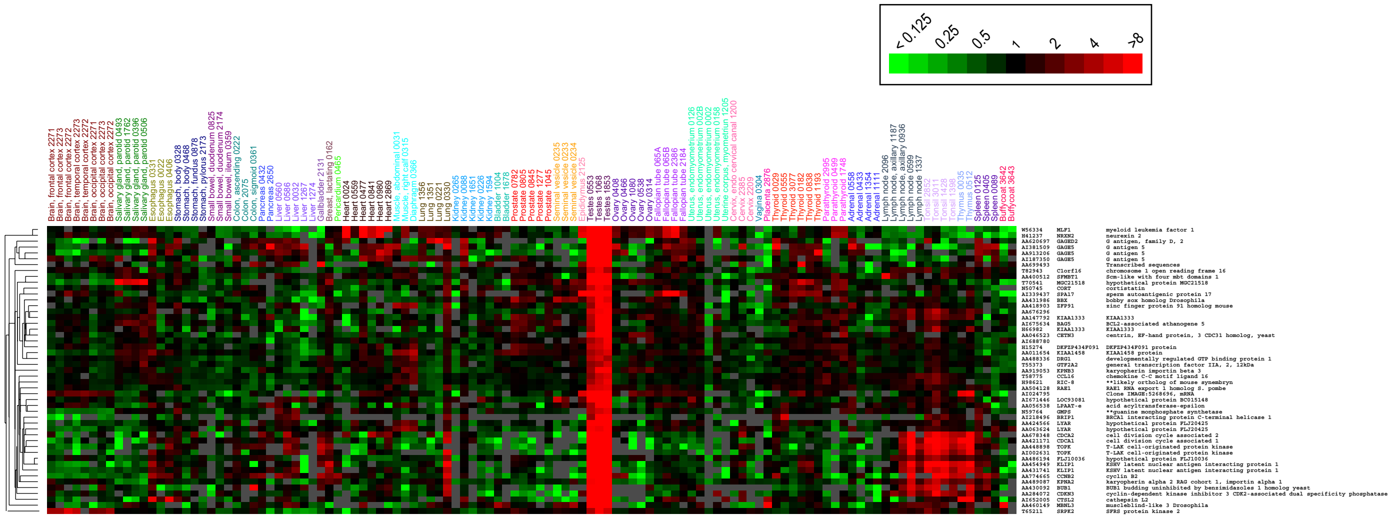

q. Ovary

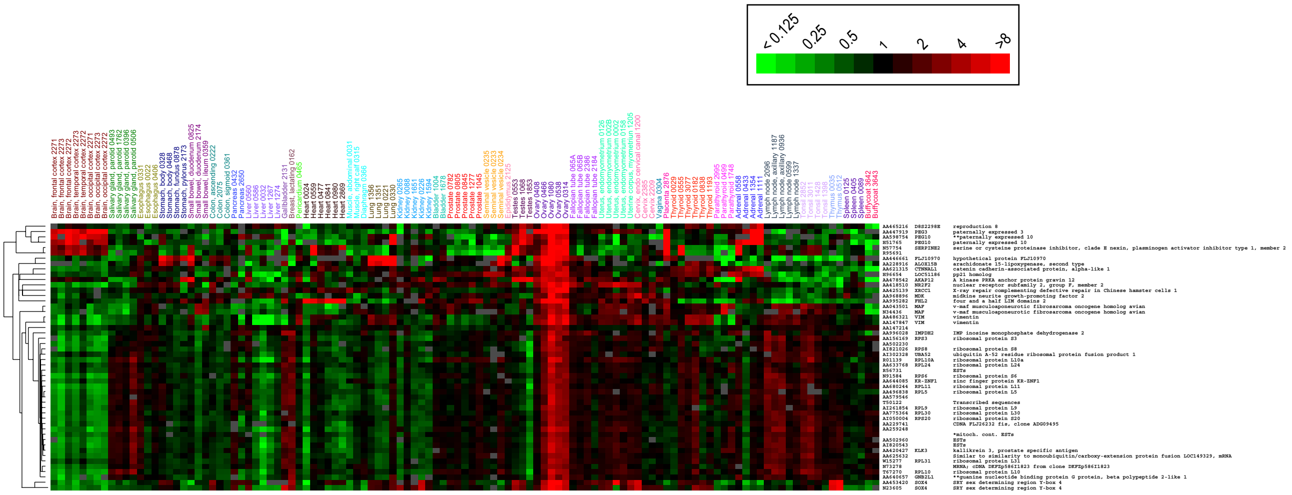

r. Fallopian tube

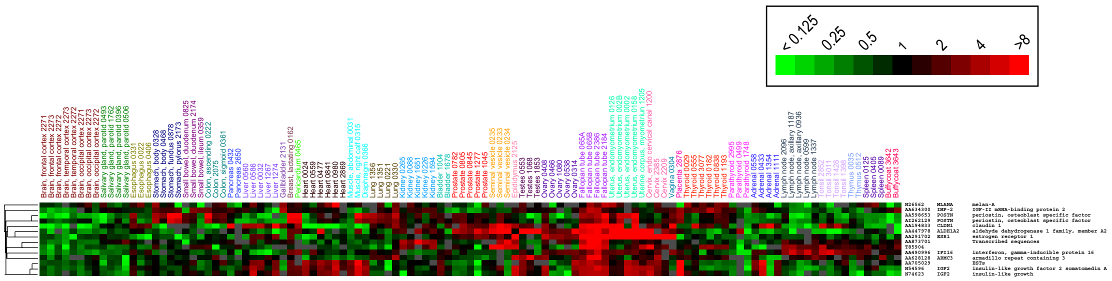

s. Uterus

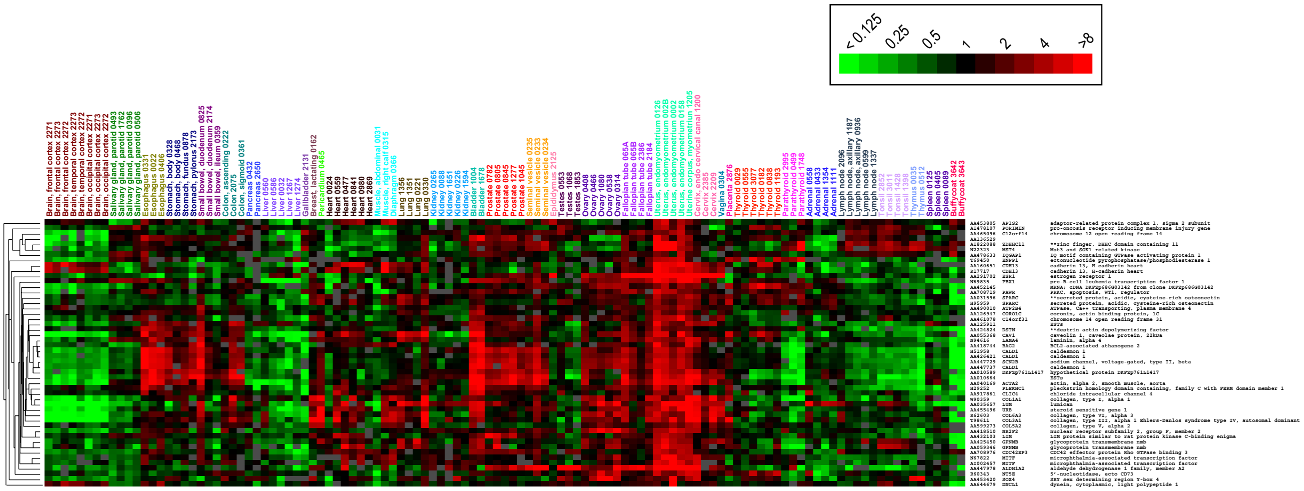

< 0.125
0.25
0.5
1
2
4
>8
Brain, frontal cortex 2271
Brain, frontal cortex 2273
Brain, frontal cortex 2272
Brain, temporal cortex 2273
Brain, temporal cortex 2272
Brain, occipital cortex 2271
Brain, occipital cortex 2273
Brain, occipital cortex 2272
Salivary gland, parotid 0493
Salivary gland, parotid 1762
Salivary gland, parotid 0396
Salivary gland, parotid 0506
Esophagus 0331
Esophagus 0022
Esophagus 0406
Stomach, body 0328
Stomach, body 0468
Stomach, fundus 0878
Stomach, pylorus 2173
Small bowel, duodenum 0825
Small bowel, duodenum 2174
Small bowel, ileum 0359
Colon, ascending 0222
Colon 2075
Colon, sigmoid 0361
Pancreas 0432
Pancreas 2650
Liver 0560
Liver 0586
Liver 0032
Liver 1267
Liver 1274
Gallbladder 2131
Breast, lactating 0162
Pericardium 0465
Heart 0024
Heart 0559
Heart 0477
Heart 0841
Heart 0980
Heart 2869
Muscle, abdominal 0031
Muscle, right calf 0315
Diaphragm 0366
Lung 1356
Lung 1351
Lung 0221
Lung 0330
Kidney 0265
Kidney 0088
Kidney 1651
Kidney 0226
Kidney 1594
Bladder 1004
Bladder 1678
Prostate 0782
Prostate 0805
Prostate 0845
Prostate 1277
Prostate 1045
Seminal vesicle 0235
Seminal vesicle 0233
Seminal vesicle 0234
Epididymus 2125
Testes 0553
Testes 1068
Testes 1853
Ovary 0408
Ovary 0466
Ovary 1080
Ovary 0538
Ovary 0314
Fallopian tube 065A
Fallopian tube 065B
Fallopian tube 2386
Fallopian tube 2184
Uterus, endomyometrium 0126
Uterus, endomyometrium 002B
Uterus, endomyometrium 0002
Uterus, endomyometrium 0158
Uterine corpus, myometrium 1205
Cervix, endo cervical canal 1200
Cervix 2385
Cervix 2209
Vagina 0304
Placenta 2876
Thyroid 0029
Thyroid 0555
Thyroid 3077
Thyroid 0182
Thyroid 0838
Thyroid 1193
Parathyroid 2995
Parathyroid 0499
Parathyroid 1748
Adrenal 0558
Adrenal 0433
Adrenal 1354
Adrenal 1111
Lymph node 2096
Lymph node, axillary 1187
Lymph node, axillary 0936
Lymph node 0599
Lymph node 1337
Tonsil 2852
Tonsil 3011
Tonsil 1428
Tonsil 1398
Thymus 0035
Thymus 0512
Spleen 0125
Spleen 0405
Spleen 0089
Buffycoat 3642
Buffycoat 3643
AA453805 AP1S2 adaptor-related protein complex 1, sigma 2 subunit
AI478107 PORIMIN pro-oncosis receptor inducing membrane injury gene
AA465096 C12orf14 chromosome 12 open reading frame 14
AA136529
AI822088 ZDHHC11 **zinc finger, DHHC domain containing 11
N22323 MST4 Mst3 and SOK1-related kinase
AA478633 IQGAP1 IQ motif containing GTPase activating protein 1
T69450 ENPP1 ectonucleotide pyrophosphatase/phosphodiesterase 1
AA160651 CDH13 cadherin 13, H-cadherin heart
R17717 CDH13 cadherin 13, H-cadherin heart
AA291702 ESR1 estrogen receptor 1
N69835 PBX1 pre-B-cell leukemia transcription factor 1
AA452145 MRNA; cDNA DKFZp686G03142 from clone DKFZp686G03142
AA708719 PAWR PRKC, apoptosis, WT1, regulator
AA031596 SPARC **secreted protein, acidic, cysteine-rich osteonectin
H95959 SPARC secreted protein, acidic, cysteine-rich osteonectin
AA490010 ATP2B4 ATPase, Ca++ transporting, plasma membrane 4
AA126947 CORO1C coronin, actin binding protein, 1C
AA461078 C14orf31 chromosome 14 open reading frame 31
AA125911 ESTs
AA424824 DSTN **destrin actin depolymerizing factor
AA055368 CAV1 caveolin 1, caveolae protein, 22kDa
N94616 LAMA4 laminin, alpha 4
AA418744 BAG2 BCL2-associated athanogene 2
H51958 CALD1 caldesmon 1
AA426421 CALD1 caldesmon 1
AA447729 SCN2B sodium channel, voltage-gated, type II, beta
AA447737 CALD1 caldesmon 1
AA010589 DKFZp761L1417 hypothetical protein DKFZp761L1417
AA010664 ESTs
AA040169 ACTA2 actin, alpha 2, smooth muscle, aorta
H29252 PLEKHC1 pleckstrin homology domain containing, family C with FERM domain member 1
AA917861 CLIC4 chloride intracellular channel 4
W90359 COL1A1 collagen, type I, alpha 1
AA035657 LUM lumican
AA455496 URB steroid sensitive gene 1
R62603 COL6A3 collagen, type VI, alpha 3
T98611 COL3A1 collagen, type III, alpha 1 Ehlers-Danlos syndrome type IV, autosomal dominant
AA599273 COL5A2 collagen, type V, alpha 2
AA418510 NR2F2 nuclear receptor subfamily 2, group F, member 2
AA432103 LIM LIM protein similar to rat protein kinase C-binding enigma
AA425450 GPNMB glycoprotein transmembrane nmb
AA059346 GPNMB glycoprotein transmembrane nmb
AA708976 CDC42EP3 CDC42 effector protein Rho GTPase binding 3
N67822 MITF microphthalmia-associated transcription factor
AI002457 MITF microphthalmia-associated transcription factor
AA447978 ALDH1A2 aldehyde dehydrogenase 1 family, member A2
R60343 NT5E 5'-nucleotidase, ecto CD73
AA453420 SOX4 SRY sex determining region Y-box 4
AA644679 DNCL1 dynein, cytoplasmic, light polypeptide 1

t. Cervix

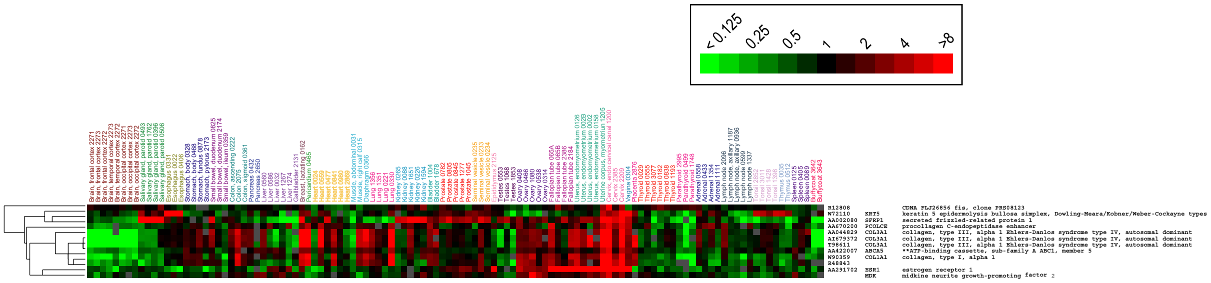
< 0.125
0.25
0.5
1
2
4
>8
R12808 CDNA FLJ26856 fis, clone PRS08123
W72110 KRT5 keratin 5 epidermolysis bullosa simplex, Dowling-Meara/Kobner/Weber-Cockayne types
AA002080 SFRP1 secreted frizzled-related protein 1
AA670200 PCOLCE procollagen C-endopeptidase enhancer
AA044829 COL3A1 collagen, type III, alpha 1 Ehlers-Danlos syndrome type IV, autosomal dominant
AI679372 COL3A1 collagen, type III, alpha 1 Ehlers-Danlos syndrome type IV, autosomal dominant
T98611 COL3A1 collagen, type III, alpha 1 Ehlers-Danlos syndrome type IV, autosomal dominant
AA422007 ABCA5 **ATP-binding cassette, sub-family A ABC1, member 5
W90359 COL1A1 collagen, type I, alpha 1
R48843
AA291702 ESR1 estrogen receptor 1
MDK midkine neurite growth-promoting factor 2

u. Thyroid

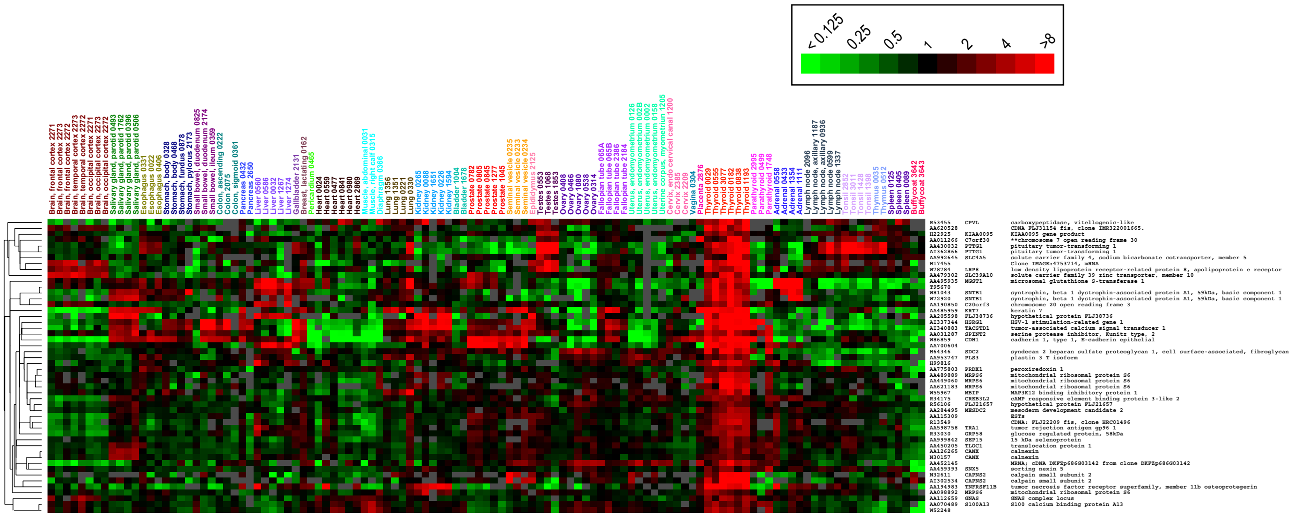

< 0.125
0.25
0.5
1
2
4
>8
R53455 CPVL carboxypeptidase, vitellogenic-like
AA620528 CDNA FLJ31154 fis, clone IMR322001665.
H22925 KIAA0095 KIAA0095 gene product
AA011266 C7orf30 **chromosome 7 open reading frame 30
AA430032 PTTG1 pituitary tumor-transforming 1
AI362866 PTTG1 pituitary tumor-transforming 1
AA992645 SLC4A5 solute carrier family 4, sodium bicarbonate cotransporter, member 5
H17455 Clone IMAGE:4753714, mRNA
W78784 LRP8 low density lipoprotein receptor-related protein 8, apolipoprotein e receptor
AA479302 SLC39A10 solute carrier family 39 zinc transporter, member 10
AA495935 MGST1 microsomal glutathione S-transferase 1
T95670
W81043 SNTB1 syntrophin, beta 1 dystrophin-associated protein A1, 59kDa, basic component 1
W72920 SNTB1 syntrophin, beta 1 dystrophin-associated protein A1, 59kDa, basic component 1
AA190850 C20orf3 chromosome 20 open reading frame 3
AA485959 KRT7 keratin 7
AA205598 FLJ38736 hypothetical protein FLJ38736
AI337344 HSRG1 HSV-1 stimulation-related gene 1
AI340883 TACSTD1 tumor-associated calcium signal transducer 1
AA031287 SPINT2 serine protease inhibitor, Kunitz type, 2
W86859 CDH1 cadherin 1, type 1, E-cadherin epithelial
AA700604
H64346 SDC2 syndecan 2 heparan sulfate proteoglycan 1, cell surface-associated, fibroglycan
AA953747 PLS3 plastin 3 T isoform
H99816
AA775803 PRDX1 peroxiredoxin 1
AA489889 MRPS6 mitochondrial ribosomal protein S6
AA449060 MRPS6 mitochondrial ribosomal protein S6
AA621183 MRPS6 mitochondrial ribosomal protein S6
W55967 MBIP MAP3K12 binding inhibitory protein 1
R34175 CREB3L2 cAMP responsive element binding protein 3-like 2
R56106 FLJ21657 hypothetical protein FLJ21657
AA284495 MESDC2 mesoderm development candidate 2
AA115309 ESTs
R13549 CDNA: FLJ22209 fis, clone HRC01496
AA598758 TRA1 tumor rejection antigen gp96 1
R33030 GRP58 glucose regulated protein, 58kDa
AA999842 SEP15 15 kDa selenoprotein
AA450205 TLOC1 translocation protein 1
AA126265 CANX calnexin
N30157 CANX calnexin
AA452145 MRNA; cDNA DKFZp686G03142 from clone DKFZp686G03142
AA459393 SNX5 sorting nexin 5
N32611 CAPNS2 calpain small subunit 2
AI302534 CAPNS2 calpain small subunit 2
AA194983 TNFRSF11B tumor necrosis factor receptor superfamily, member 11b osteoprotegerin
AA098892 MRPS6 mitochondrial ribosomal protein S6
AA112659 GNAS GNAS complex locus
AA070489 S100A13 S100 calcium binding protein A13
W52248

v. Parathyroid

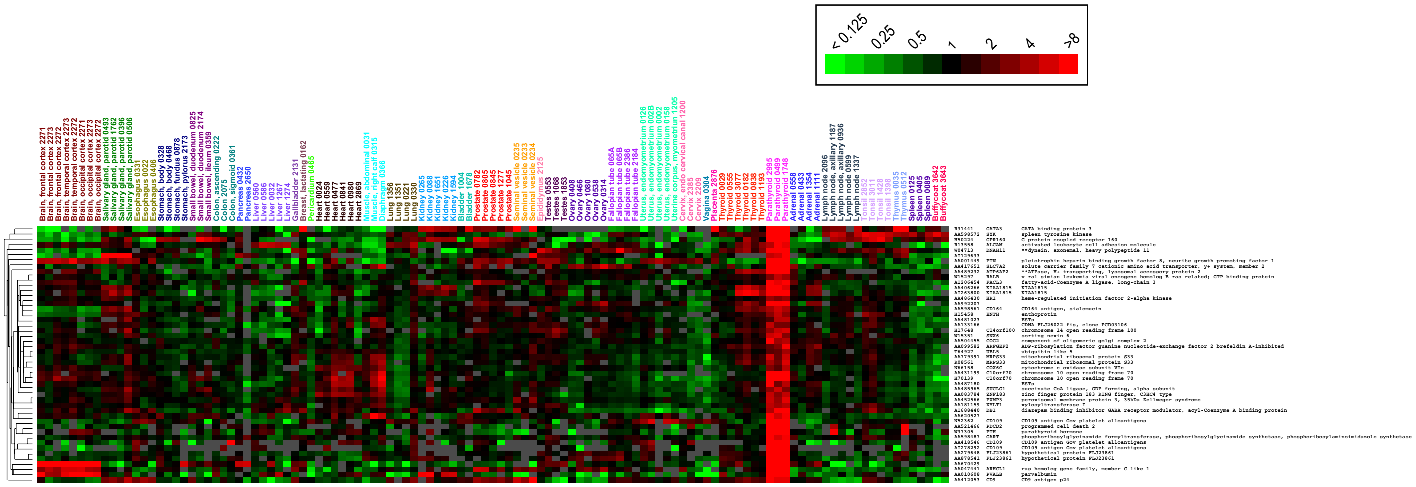

w. Adrenal

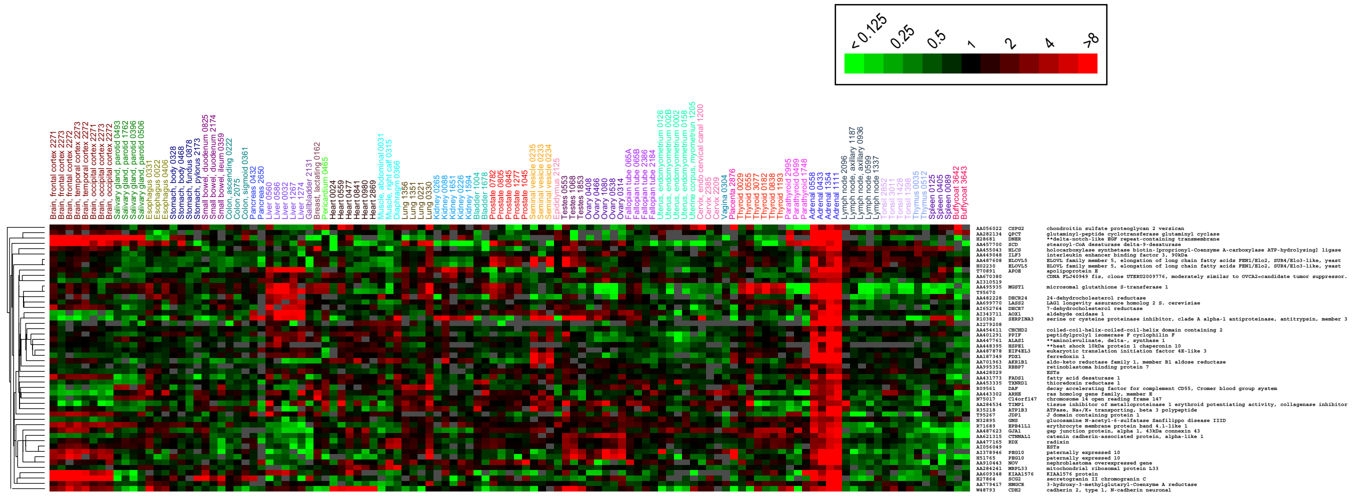

x. Lymph node

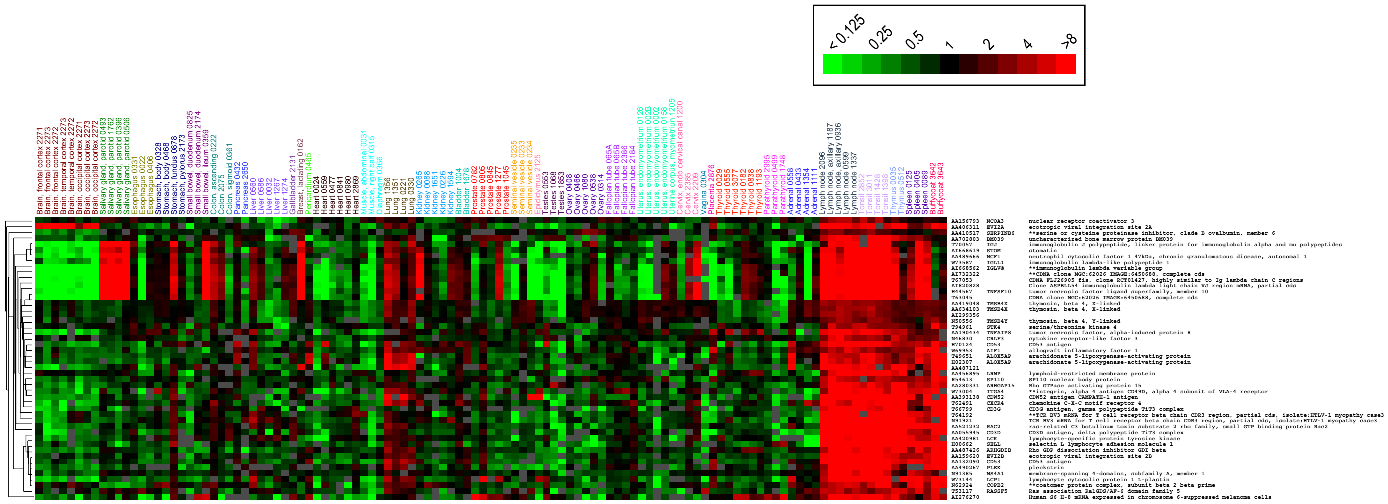
<0.125
0.25
0.5
1
2
4
>8
AA156793 NCOA3 nuclear receptor coactivator 3
AA406311 EVI2A ecotropic viral integration site 2A
AA410517 SERPINB6 **serine or cysteine proteinase inhibitor, clade B ovalbumin, member 6
AA702803 BM039 uncharacterized bone marrow protein BM039
T70057 IGJ immunoglobulin J polypeptide, linker protein for immunoglobulin alpha and mu polypeptides
AI668619 STOM stomatin
AA489666 NCF1 neutrophil cytosolic factor 1 47kDa, chronic granulomatous disease, autosomal 1
W73587 IGLL1 immunoglobulin lambda-like polypeptide 1
AI668562 IGLV@ **immunoglobulin lambda variable group
AI732322 **CDNA clone MGC:62026 IMAGE:6450688, complete cds
T67053 CDNA FLJ26905 fis, clone RCT01427, highly similar to Ig lambda chain C regions
AI820828 Clone ASPBLL54 immunoglobulin lambda light chain VJ region mRNA, partial cds
H44567 TNFSF10 tumor necrosis factor ligand superfamily, member 10
T63045 CDNA clone MGC:62026 IMAGE:6450688, complete cds
AA419048 TMSB4X thymosin, beta 4, X-linked
AA634103 TMSB4X thymosin, beta 4, X-linked
AI299356
N50556 TMSB4Y thymosin, beta 4, Y-linked
T94961 STK4 serine/threonine kinase 4
AA190434 TNFAIP8 tumor necrosis factor, alpha-induced protein 8
N46830 CRLF3 cytokine receptor-like factor 3
H70124 CD53 CD53 antigen
W69953 AIF1 allograft inflammatory factor 1
T49651 ALOX5AP arachidonate 5-lipoxygenase-activating protein
H02307 ALOX5AP arachidonate 5-lipoxygenase-activating protein
AA487121
AA456895 LRMP lymphoid-restricted membrane protein
R54613 SP110 SP110 nuclear body protein
AA280331 ARHGAP15 Rho GTPase activating protein 15
W73004 ITGA4 **integrin, alpha 4 antigen CD49D, alpha 4 subunit of VLA-4 receptor
AA393138 CDW52 CDW52 antigen CAMPATH-1 antigen
T62491 CXCR4 chemokine C-X-C motif receptor 4
T66799 CD3G CD3G antigen, gamma polypeptide TiT3 complex
T64192 **TCR BV3 mRNA for T cell receptor beta chain CDR3 region, partial cds, isolate:HTLV-1 myopathy case3
N91921 TCR BV3 mRNA for T cell receptor beta chain CDR3 region, partial cds, isolate:HTLV-1 myopathy case3
AA521232 RAC2 ras-related C3 botulinum toxin substrate 2 rho family, small GTP binding protein Rac2
AA055945 CD3D CD3D antigen, delta polypeptide TiT3 complex
AA420981 LCK lymphocyte-specific protein tyrosine kinase
H00662 SELL selectin L lymphocyte adhesion molecule 1
AA487426 ARHGDIB Rho GDP dissociation inhibitor GDI beta
AA159620 EVI2B ecotropic viral integration site 2B
AA132090 CD53 CD53 antigen
AA490267 PLEK pleckstrin
N91385 MS4A1 membrane-spanning 4-domains, subfamily A, member 1
W73144 LCP1 lymphocyte cytosolic protein 1 L-plastin
N62924 COPB2 **coatomer protein complex, subunit beta 2 beta prime
T53117 RASSF5 Ras association RalGDS/AF-6 domain family 5
AI276270 Human S6 H-8 mRNA expressed in chromosome 6-suppressed melanoma cells

y. Tonsil

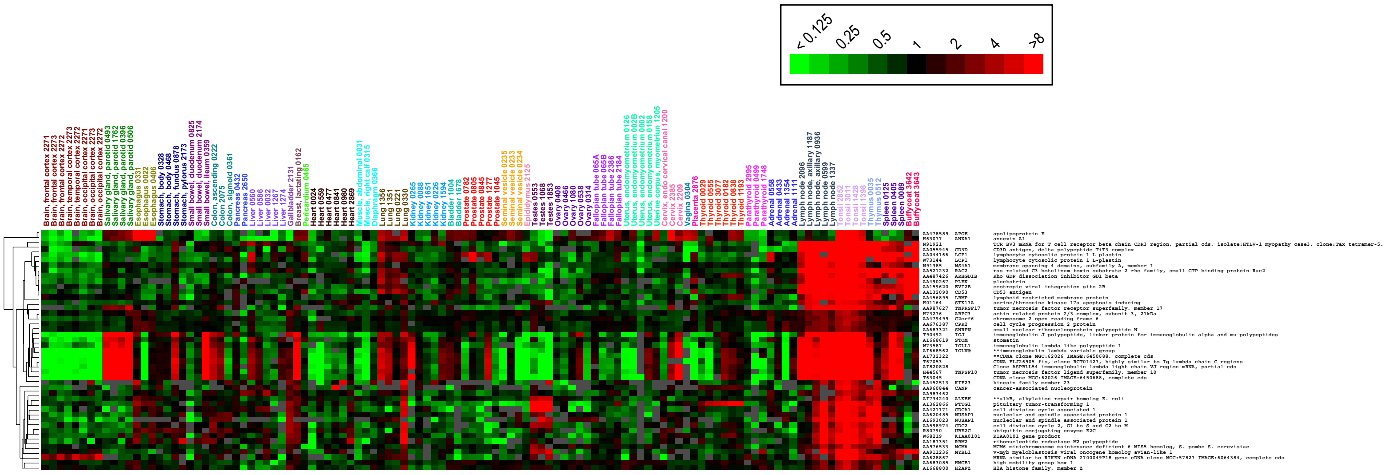

z. Thymus

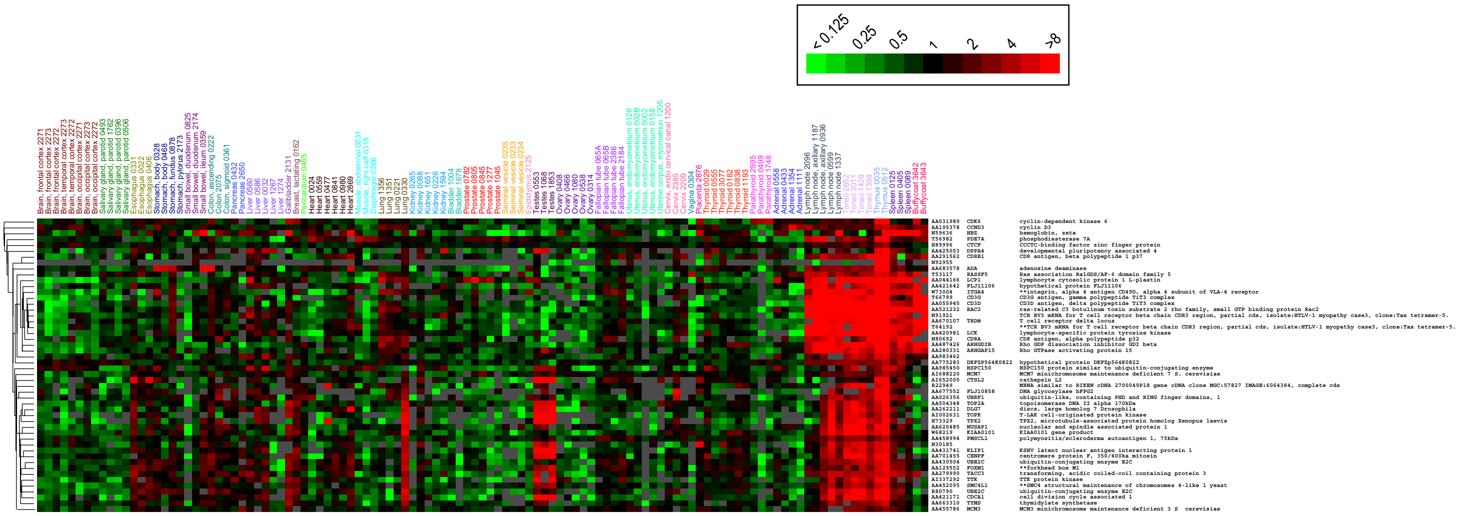

aa. Spleen

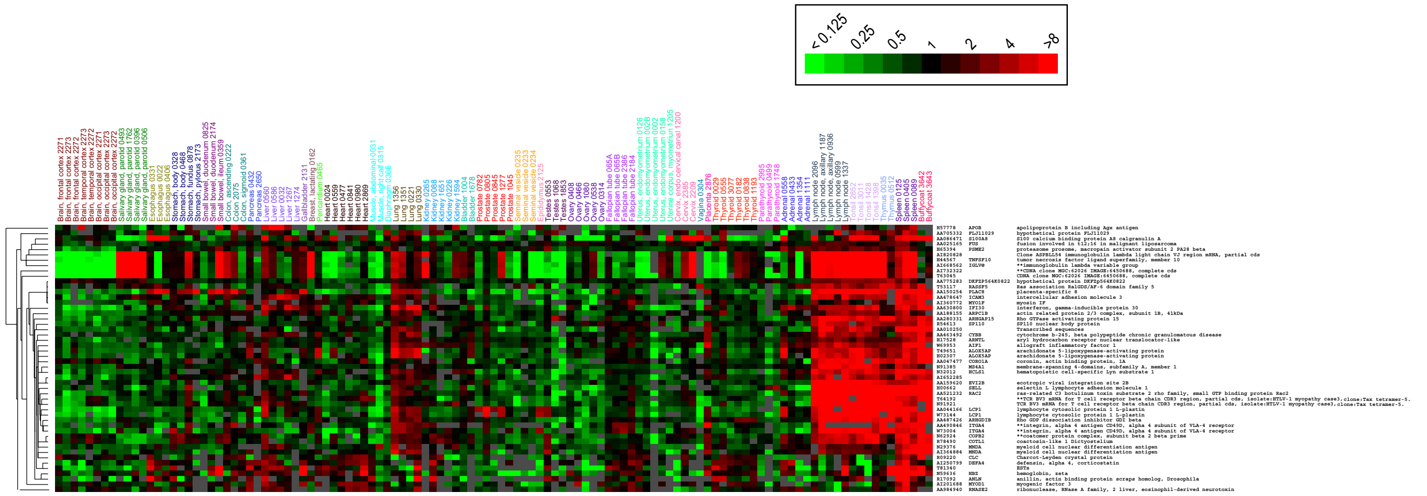

< 0.125
0.25
0.5
1
2
4
>8
H57778 APOB apolipoprotein B including Agx antigen
AA705332 FLJ11029 hypothetical protein FLJ11029
AA086471 S100A8 S100 calcium binding protein A8 calgranulin A
AA025165 FUS fusion involved in t12;16 in malignant liposarcoma
H65394 PSME2 proteasome prosome, macropain activator subunit 2 PA28 beta
AI820828 Clone ASPBLL54 immunoglobulin lambda light chain VJ region mRNA, partial cds
H44567 TNFSF10 tumor necrosis factor ligand superfamily, member 10
AI668562 IGLV@ **immunoglobulin lambda variable group
AI732322 **CDNA clone MGC:62026 IMAGE:6450688, complete cds
T63045 CDNA clone MGC:62026 IMAGE:6450688, complete cds
AA775283 DKFZP564K0822 hypothetical protein DKFZp564K0822
T53117 RASSF5 Ras association RalGDS/AF-6 domain family 5
AA150254 PLAC8 placenta-specific 8
AA478647 ICAM3 intercellular adhesion molecule 3
AI360772 MYO1F myosin IF
AA630800 IFI30 interferon, gamma-inducible protein 30
AA188155 ARPC1B actin related protein 2/3 complex, subunit 1B, 41kDa
AA280331 ARHGAP15 Rho GTPase activating protein 15
R54613 SP110 SP110 nuclear body protein
AA010250 Transcribed sequences
AA463492 CYBB cytochrome b-245, beta polypeptide chronic granulomatous disease
H17528 ARNTL aryl hydrocarbon receptor nuclear translocator-like
W69953 AIF1 allograft inflammatory factor 1
T49651 ALOX5AP arachidonate 5-lipoxygenase-activating protein
H02307 ALOX5AP arachidonate 5-lipoxygenase-activating protein
AA047477 CORO1A coronin, actin binding protein, 1A
N91385 MS4A1 membrane-spanning 4-domains, subfamily A, member 1
N32012 HCLS1 hematopoietic cell-specific Lyn substrate 1
AI652285
AA159620 EVI2B ecotropic viral integration site 2B
H00662 SELL selectin L lymphocyte adhesion molecule 1
AA521232 RAC2 ras-related C3 botulinum toxin substrate 2 rho family, small GTP binding protein Rac2
T64192 **TCR BV3 mRNA for T cell receptor beta chain CDR3 region, partial cds, isolate:HTLV-1 myopathy case3,clone:Tax tetramer-5.
N91921 TCR BV3 mRNA for T cell receptor beta chain CDR3 region, partial cds, isolate:HTLV-1 myopathy case3,clone:Tax tetramer-5.
AA044166 LCP1 lymphocyte cytosolic protein 1 L-plastin
W73144 LCP1 lymphocyte cytosolic protein 1 L-plastin
AA487426 ARHGDIB Rho GDP dissociation inhibitor GDI beta
AA490846 ITGA4 **integrin, alpha 4 antigen CD49D, alpha 4 subunit of VLA-4 receptor
W73004 ITGA4 **integrin, alpha 4 antigen CD49D, alpha 4 subunit of VLA-4 receptor
N62924 COPB2 **coatomer protein complex, subunit beta 2 beta prime
R78490 COTL1 coactosin-like 1 Dictyostelium
N29376 MNDA myeloid cell nuclear differentiation antigen
AI364884 MNDA myeloid cell nuclear differentiation antigen
R09220 CLC Charcot-Leyden crystal protein
AI250799 DEFA4 defensin, alpha 4, corticostatin
T81340 ESTs
N59636 HBZ hemoglobin, zeta
R17092 ANLN anillin, actin binding protein scraps homolog, Drosophila
AI201688 MYOD1 myogenic factor 3
AA984940 RNASE2 ribonuclease, RNase A family, 2 liver, eosinophil-derived neurotoxin

cc. Buffy coat

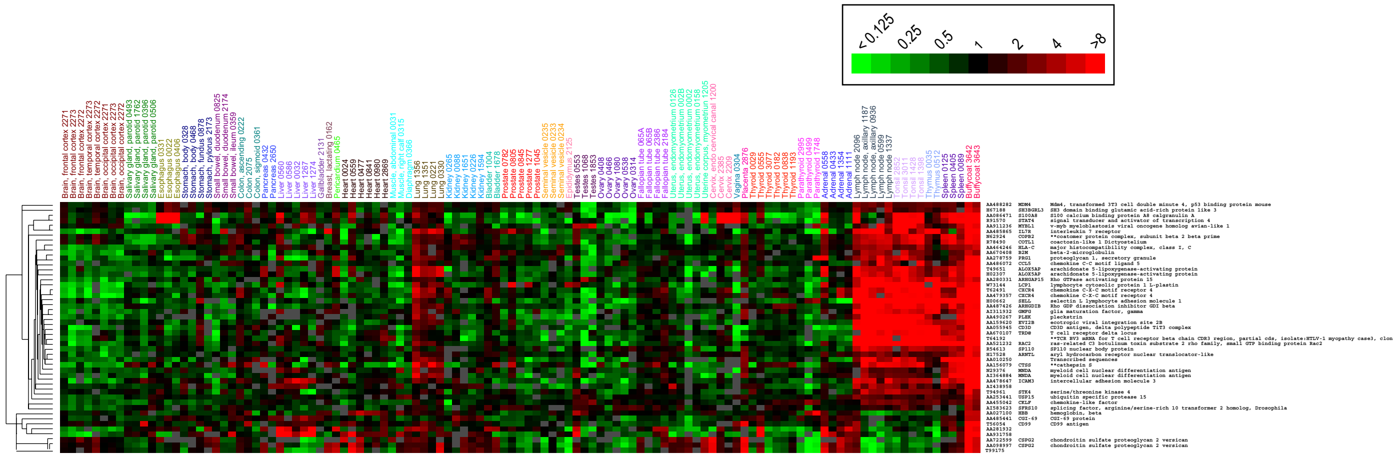

Supplement: Additional File 8 — A figure showing highly abundant tissue-specific gene expression. Highly-abundant tissue specific transcripts were defined for each tissue type as the top (capped at 50 genes) tissue specific transcripts, identified using the SAM method, from the 1000 most abundantly expressed transcripts in the full dataset. a, brain; b, salivary gland; c, esophagus; d, stomach; e, small bowel; f, colon; g, pancreas; h, liver; i, heart; j, skeletal muscle; k, lung; l, kidney; m, bladder;n, prostate; o, seminal vesicle; p, testis; q, ovary; r, fallopian tube; s, uterus; t, cervix, u, thyroid; v, parathyroid; w, adrenal; x, lymph node; y, tonsil; z, thymus; aa, spleen; bb, buffy coat [file gb-2005-6-3-r22-S8.pdf]
